# Elucidation of radical- and oxygenate-driven paths in zeolite-catalysed conversion of methanol and methyl chloride to hydrocarbons

**DOI:** 10.1038/s41929-022-00808-0

**Published:** 2022-06-27

**Authors:** Alessia Cesarini, Sharon Mitchell, Guido Zichittella, Mikhail Agrachev, Stefan P. Schmid, Gunnar Jeschke, Zeyou Pan, Andras Bodi, Patrick Hemberger, Javier Pérez-Ramírez

**Affiliations:** 1 Institute for Chemical and Bioengineering, Department of Chemistry and Applied Biosciences, ETH Zurich, Zurich, Switzerland; 2 Laboratory of Physical Chemistry, Department of Chemistry and Applied Biosciences, ETH Zurich, Zurich, Switzerland; 3 Laboratory of Synchrotron Radiation and Femtochemistry, Paul Scherrer Institute, Villigen, Switzerland

**Keywords:** Catalytic mechanisms, Heterogeneous catalysis, Characterization and analytical techniques

## Abstract

Understanding hydrocarbon generation in the zeolite-catalysed conversions of methanol and methyl chloride requires advanced spectroscopic approaches to distinguish the complex mechanisms governing C–C bond formation, chain growth and the deposition of carbonaceous species. Here operando photoelectron photoion coincidence (PEPICO) spectroscopy enables the isomer-selective identification of pathways to hydrocarbons of up to C_14_ in size, providing direct experimental evidence of methyl radicals in both reactions and ketene in the methanol-to-hydrocarbons reaction. Both routes converge to C_5_ molecules that transform into aromatics. Operando PEPICO highlights distinctions in the prevalence of coke precursors, which is supported by electron paramagnetic resonance measurements, providing evidence of differences in the representative molecular structure, density and distribution of accumulated carbonaceous species. Radical-driven pathways in the methyl chloride-to-hydrocarbons reaction(s) accelerate the formation of extended aromatic systems, leading to fast deactivation. By contrast, the generation of alkylated species through oxygenate-driven pathways in the methanol-to-hydrocarbons reaction extends the catalyst lifetime. The findings demonstrate the potential of the presented methods to provide valuable mechanistic insights into complex reaction networks.

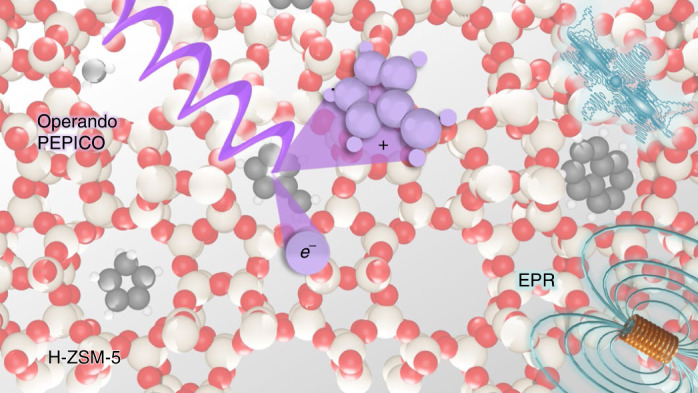

## Abstract (web summary)

The conversions of methanol or methyl chloride over zeolite catalysts are promising processes to produce valuable hydrocarbons, but their mechanisms are still not fully understood. Now these are evaluated using operando photoelectron photoion coincidence spectroscopy, which enables the direct observation of elusive intermediates such as methyl radicals or ketene.

## Main

Monosubstituted methanes, including methanol and methyl chloride (CH_3_X, X = OH and Cl, respectively), are attractive building blocks for sustainable fuels and chemicals^1–7^. These C_1_ molecules are the main products of syngas conversion, carbon dioxide hydrogenation and halogen-mediated alkane functionalization, and can be readily converted into valuable hydrocarbons over zeolite catalysts^8–13^. However, uncontrolled chain growth typically results in broad product distributions and the deposition of heavy carbonaceous species, leading to suboptimal catalyst selectivity and stability. Towards improving productivity, extensive experimental and theoretical efforts have been devoted to understanding the mechanism of C–C bond formation and its propagation to higher hydrocarbons in the commercially applied methanol-to-hydrocarbons (MTH) reaction^1,14–18^. Detailed kinetic studies, often combined with isotopic labelling and spectroscopic experiments, evidenced the autocatalytic nature of the process, provided the basis for the widely accepted dual aromatic–olefin cycles and identified several intermediates expected to play crucial roles in the early stages and steady-state operation of the reaction (Supplementary Note 1)^19–31^. Despite these advances, several questions about the mechanism remain strongly debated, including the existence and role of radical species, the mechanism of the first C–C bond formation and subsequent paths to the early aromatic hydrocarbon pool (HCP) species. Furthermore, archetypical dissolution–extraction experiments and spectroscopic measurements have provided limited insight into the specific structures of the coke precursors and their evolution into carbonaceous species deposited within the zeolite catalyst (Supplementary Note 1).

Comparatively, mechanistic understanding of the methyl chloride-to-hydrocarbons (MCTH) reaction is less developed. Previous studies have focused on kinetic aspects, showing broadly similar product distributions to the MTH process, attributed to similar confinement effects within the zeolite pore networks on HCP development^11,12,32–34^. Nevertheless, the absence of oxygenates and the faster catalyst deactivation reported in the MCTH process suggest that important mechanistic distinctions exist. A systematic comparison of the pathways for the activation and subsequent transformation of CH_3_Cl and CH_3_OH could help decouple direct C–C bond formation routes from oxygenate-driven chain growth. However, the direct observation of short-lived active intermediates, such as ketene and methyl radicals, under operando conditions remains highly challenging because of the difficulty in discriminating them from stable spectator species due to the limited sensitivity of established methods. Advanced time-resolved spectroscopic techniques that can distinguish compounds present in low concentrations and distinct isomeric forms could provide new insights to help resolve some of the long-standing debates.

**Fig. 1:**
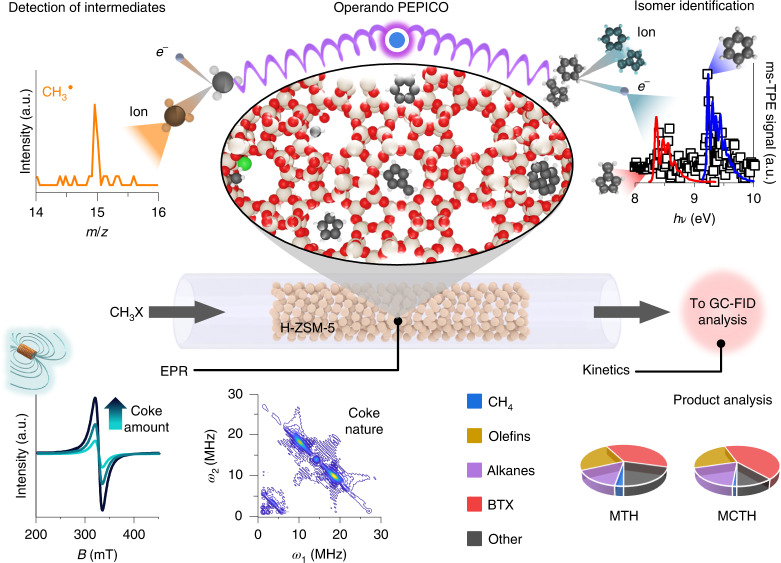
Approach to mapping the catalysed generation and evolution of hydrocarbons. Schematic of the multi-technique strategy used in this study to unravel the growth of the carbon chain, from the formation of the first C–C bond to the generation of coke in MTH and MCTH over H-ZSM-5. The comparative study of these two C_1_ platform molecules enables the main pathways of oxygenate- and hydrocarbon-driven mechanisms to be decoupled and elucidated. Accordingly, operando PEPICO enables the isomer-selective identification of reaction intermediates and coke precursors. This is complemented with EPR measurements that provide insights into the representative molecular structure, density and distribution of deposited carbonaceous species, and kinetic analysis for assessment of the catalytic activity, selectivity and stability. GC-FID, gas chromatography with flame ionization detection.

Herein, we analyse hydrocarbons desorbed from a representative H-ZSM-5 zeolite catalyst during MTH and MCTH processes using operando PEPICO spectroscopy under relevant reaction conditions. This technique has shed light on complex reaction networks^35–37^ and enables the quantitative, isomer-selective identification of all short- and long-lived species up to approximately C_14_ in size. By performing temperature-dependent studies, operando PEPICO enables the discrimination of primary and secondary intermediates and tertiary products. A complementary study of the used catalysts via electron paramagnetic resonance (EPR)^38,39^ revealed distinctions in the representative molecular structure, density and distribution of the carbonaceous species deposited in the zeolite pore network. In combination with kinetic analysis, the adopted approach (Fig. 1) provides high molecular resolution of the species formed, enabling experimental verification of several open questions about the reaction mechanisms.

## Results

### Evaluation of the reaction kinetics

**Fig. 2:**
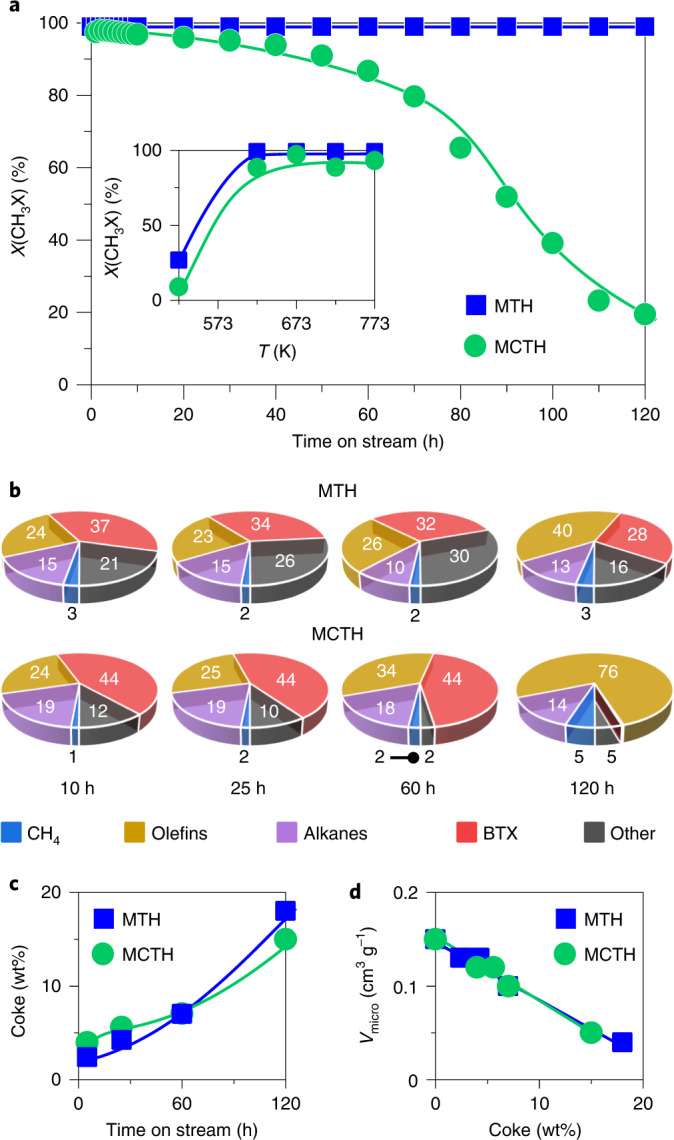
Comparative performance of the zeolite catalyst. **a**, Reactant conversion in the MTH and MCTH processes as a function of the time on stream and temperature (inset) over H-ZSM-5. **b**, Product distribution as a function of time on stream averaged over a period of 1 h. **c**, Amount of coke species deposited measured via TGA as a function of time on stream. **d**, Variation in the micropore volume (*V*_micro_) as a function of the amount of coke. Conditions: CH_3_X:He = 1:1, *F*_T_ = 20 cm^3^ STP min^−1^, *W*_cat_ = 0.6 g, *T* = 673 K, *P* = 1 bar. Source data

Preliminary insights into the similarities and differences between MTH and MCTH processes were gained by studying the steady-state kinetics of the reactions over an H-ZSM-5 zeolite catalyst with a nominal Si/Al ratio of 40 under equivalent conditions (Fig. 2; Supplementary Figs. 1 and 2). The reactivity was assessed by calculating the conversion (*X*(CH_3_X)) based on the concentration of CH_3_X at the reactor inlet and outlet. According to common practice, the reactivity in the MTH process was also evaluated considering both CH_3_OH and dimethyl ether ((CH_3_)_2_O) as reactants since an equilibrium is typically established between the two^1^. Since no (CH_3_)_2_O was observed at 100% conversion, recalculation of the conversion and product distribution does not alter the conclusions below (Fig. 2; Supplementary Figs. 1 and 2). Both reactions showed a strong temperature dependence, and their light-off curves were found to be comparable, particularly when considering (CH_3_)_2_O as a reactant (Fig. 2a; Supplementary Fig. 2a). This points to similar kinetic behaviour of the MTH and MCTH reactions over H-ZSM-5, which contrasts with previous findings obtained over H-SAPO-34 zeolite catalysts^11^. In this study by Olsbye et al., lower conversions for the MCTH reaction were attributed to the rapid rupture of Si–O–Al bonds due to interaction with HCl. The similar conversions shown in our study suggest that the MFI-type framework of H-ZSM-5 offers higher stability^11,33^ and that the leaving group does not severely influence the intrinsic kinetics of MTH and MCTH. The products are reported in five main groups: CH_4_, C_2_–C_4_ olefins, C_2_–C_4_ alkanes, benzene–toluene–xylene (BTX) and other species, including C_5+_ hydrocarbons. At 673 K, while the generation of CH_4_, olefins and alkanes was similar, the formation of BTX was found more favoured in the MCTH process (52% selectivity) compared with MTH (38% selectivity). An opposite trend was observed for C_5+_ hydrocarbons, with selectivities of 4% and 12% for MCTH and MTH, respectively. Contrariwise, a rise in the reaction temperature (*T*) led to an increase in the formation of olefins at the expense of BTX in the MCTH process and especially in the MTH reaction (Supplementary Fig. 1a), which is consistent with other studies^12,32^. A stronger rise in CH_4_ generation was observed in MCTH compared with the MTH process, which suggests that a lower rate of *trans*-hydrogenation reactions in MCTH than in MTH is favoured by the presence of (CH_3_)_2_O (ref. ^40^). In line with the literature^32^, (CH_3_)_2_O, formed by the reversible dehydration of CH_3_OH, was not observed in the MTH reaction at 100% conversion. To extract additional kinetic information, the product distribution was also compared at approximately 65% CH_3_X conversion, which was achieved by adjusting the space velocity (Supplementary Fig. 1b). Under these conditions, a considerable amount of (CH_3_)_2_O was observed in the MTH process (27% selectivity). In addition, the formation of olefins and C_5+_ hydrocarbons was favoured at 65% conversion compared with full CH_3_X conversion at 673 K (olefin selectivity: 45% versus 26% for MTH, 40% versus 24% for MCTH; other selectivity: 14% versus 12% for MTH, 11% versus 4% for MCTH) at the expense of BTX (5% versus 38% for MTH, 35% versus 52% for MCTH). These common trends agree with the expected comparable mechanism of chain growth in the MTH and MCTH processes, in which olefins and C_5+_ hydrocarbons act as precursors for the formation of aromatic compounds.

Comparison of the time-dependent reactivity showed that full CH_3_X conversion could be maintained for 120 h in the MTH process (Fig. 2a,b). The product distribution remained virtually unaffected for the first 60 h on stream, although olefins increased from approximately 26% to 40% after 120 h in the MTH reaction at the expense of BTX (32% versus 28%) and C_5+_ hydrocarbons (30% versus 16%). By contrast, CH_3_Cl conversion gradually decreased to approximately 80% over the first 60 h before dropping more sharply to less than 20% after 120 h on stream. The product distribution also remained stable during the first 60 h, before BTX production decreased, ultimately reaching negligible production at the end of the run, while that of olefins increased. The used catalysts were characterized after selected times on stream in the MTH and MCTH processes to quantify the amount and location of carbonaceous coke species deposited and to confirm the effects of the reaction on the crystallinity and acidic properties of the H-ZSM-5 zeolite. Consistent with previous reports that have shown coking as a deactivation pathway in both reactions^34,41,42^, thermogravimetric analysis (TGA) provided evidence of the accumulation of substantial carbonaceous deposits (up to 15 wt%) (Fig. 2c). Notably, coke accumulation was faster in the early stages of the MCTH process compared with MTH, which is in line with the higher tendency of this C_1_ platform molecule to generate BTX. However, the rate of coke deposition subsequently slowed and, after 120 h on stream, the coke content was slightly lower than in the catalyst used in the MTH reaction. This reduction in coking rate is consistent with the strong decrease in the production of BTX and C_5+_ hydrocarbons observed during the long-term test for the MCTH reaction (Fig. 2a,c), which have been identified as important classes of coke precursors^41,42^. A linear inverse correlation between the micropore volume and coke content was found (Fig. 2d). This indicates that carbonaceous species evolve and accumulate similarly within the pore network, despite the different rate of deposition, which is in line with the results from EPR (vide infra). X-ray diffraction patterns of the used zeolite catalysts after the oxidative removal of coke species showed no alteration of the crystal structure after either reaction (Supplementary Fig. 3). By contrast, the infrared spectroscopy study of adsorbed pyridine highlighted substantial changes in the acidic properties after the MCTH reaction, indicating decreased concentrations of Brønsted acid sites and increased concentrations of Lewis acid sites (Supplementary Table 1). These observations agree with previous reports on the impact of treatment with gaseous HCl-containing streams, a by-product formed in equimolar amounts in the MCTH process, on the zeolite properties^33,34^.

### Paths for hydrocarbon formation

**Fig. 3:**
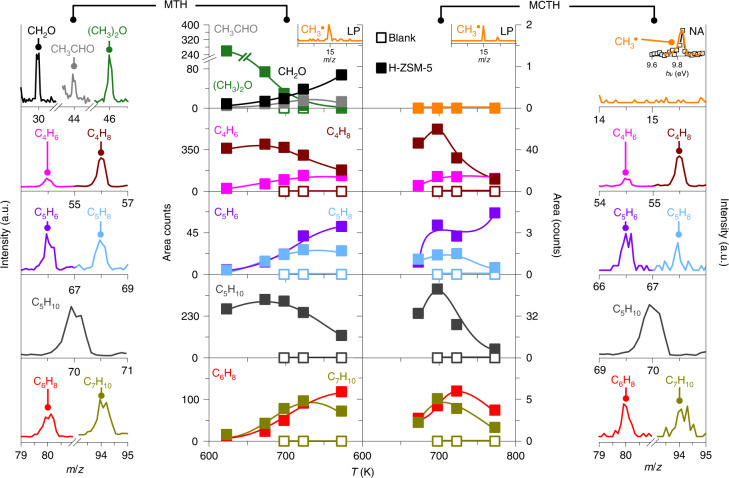
Evolution of reaction intermediates. Peak areas of the main reaction intermediates as a function of temperature in the MTH and MCTH reactions over H-ZSM-5 determined using operando PEPICO. The blank reactor experiments are shown as reference (open symbols). Representative mass spectra of the products detected for MTH and MCTH processes over the zeolite at 698 K. The insets show the mass spectra of the detected CH_3_^•^ radicals during low-pressure (LP) or near-ambient (NA) experiments at 773 K, as detailed in Supplementary Figs. 9 and 11, confirming their presence under relevant conditions. The photon energies at which the signals of each chemical species were recorded in all PEPICO experiments are shown in Supplementary Figs. 5 and 7. Conditions: CH_3_X:Xe:Ar = 1.0:0.1:20.9, *F*_T_ = 22 cm^3^ STP min^−1^, *W*_cat_ = 0.05 g, *P* = 0.5 bar (insets, *P* = 0.05 bar). Source data

**Fig. 4:**
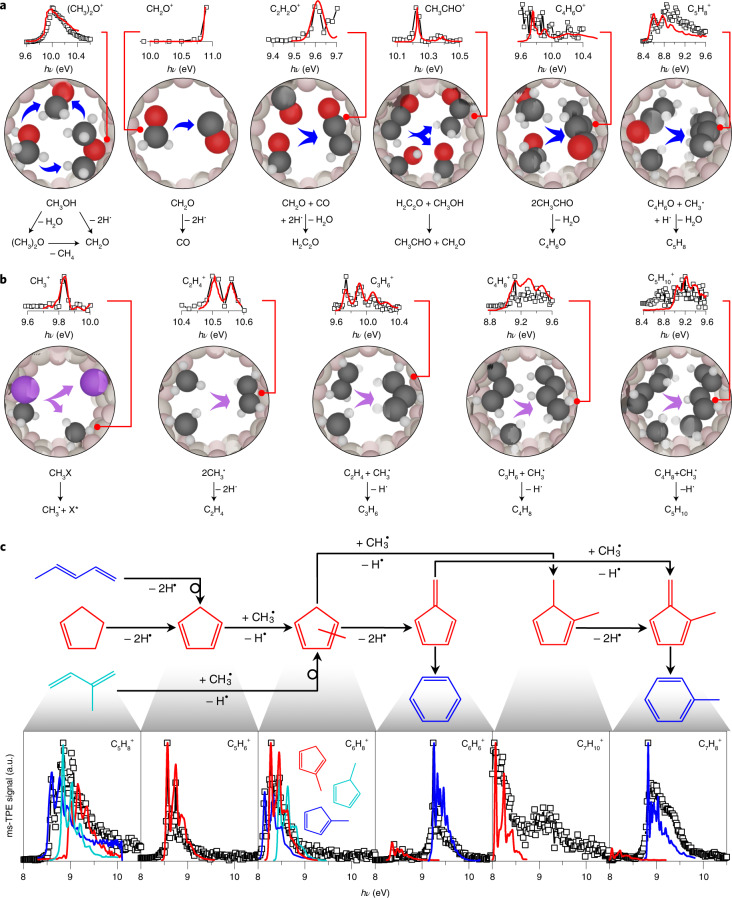
Reaction pathways of catalytic C_1_ coupling. **a**,**b**, Proposed reaction network of the oxygenate-driven reaction in the MTH process (**a**) and of the direct CH_3_^•^ radical-addition pathway in the MTH and MCTH reactions (**b**) to yield C_5_ intermediates in the micropores of H-ZSM-5. The insets on top of the molecular schemes show the ms-TPE (open squares) and reference spectra (solid lines) of the identified species for MTH in **a** and MCTH in **b**. The main identified isomers are represented by the molecular models in **a**,**b**, while other structures are shown in Supplementary Figs. 18–22. Colour code: C (dark grey), O (red), H (light grey), X (X = OH or Cl; violet). **c**, Reaction pathway for the chain-propagation reaction of C_5_H_8_ to generate benzene and toluene in the MTH process. The ms-TPE (open squares) and reference spectra (solid lines) of the identified isomeric products are also shown. Conditions as reported in Fig. 3. Source data

**Fig. 5:**
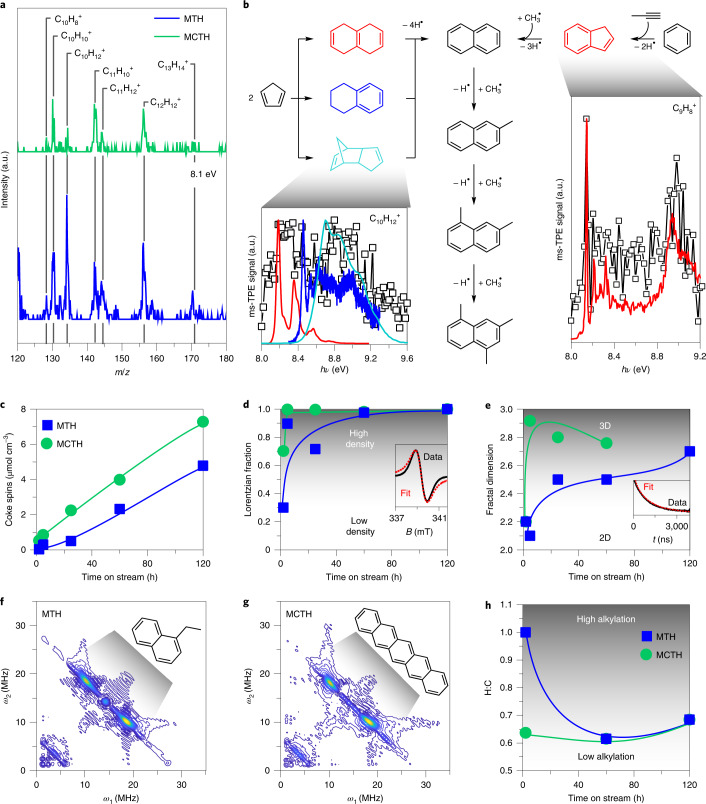
Generation and evolution of condensed carbonaceous species. **a**, Mass spectra of the C_10+_ species detected at the reactor outlet in the MTH and MCTH reactions over H-ZSM-5. **b**, Reaction pathways for the formation of naphthalene and methylated naphthalenes in MTH. The insets show the ms-TPE (open squares) and reference spectra (solid lines) of the identified products. **c**–**e**, EPR-active concentration (**c**), Lorentzian fraction (**d**) and fractal dimension (**e**) of carbonaceous deposits in the used catalysts as a function of time on stream in MTH and MCTH as determined using CW EPR and pulsed EPR, respectively. The insets in **d**,**e**, illustrate how these parameters were extracted. **f**,**g**, Weak interaction quadrant of the 2D HYSCORE spectra of the zeolite after 2 h on stream for MTH (**f**) and MCTH (**g**). The insets depict the representative molecular structures of the carbonaceous species. **h**, H:C molar ratio extracted from the 2D HYSCORE spectra as a function of time on stream in MTH and MCTH over the zeolite. Conditions: **a**,**b**, Conditions as reported in Fig. 3; **c**–**h**, CH_3_X:He = 1:1, *F*_T_ = 20 cm^3^ STP min^−1^, *W*_cat_ = 0.6 g, *T* = 673 K, *P* = 1 bar. Source data

Operando PEPICO analysis enables us to obtain detailed insight into the intermediates and products formed in the reactions over H-ZSM-5 at relevant conditions (*T* ≤ 773 K, *P* ≤ 0.5 bar), showing a wide range of hydrocarbons (C_1_–C_14_) in the MTH and MCTH processes and of oxygenates (C_1_–C_4_) exclusively in the MTH reaction (Figs. 3–5; Supplementary Figs. 4–23). As detailed in the Supplementary Methods, the reactants and intermediates or products desorbed from the catalyst are detected via photoionization with monochromatic vacuum ultraviolet light. The photoions and photoelectrons generated are detected in coincidence, revealing their mass-to-charge ratio (*m*/*z*) and their isomeric identity based on the photoion mass-selected threshold photoelectron (ms-TPE) spectrum. Two types of experiment were conducted for both reactions^37^. The first type used a packed bed of zeolite catalyst that permitted operation under near-ambient conditions (*P* = 0.5 bar) (Figs. 3 and 4; Supplementary Figs. 4–7, 14 and 17), whereas the second experiment used a zeolite-coated microreactor for low-pressure measurements (*P* = 0.05 bar) (Supplementary Figs. 8–11 and 15–17). Conversion estimates were obtained by normalizing the feed signals to a xenon (Xe) internal standard and comparing them with those obtained in blank experiments at the same temperature (90 and 65% for MTH or 50 and 35% for MCTH at near-ambient or low pressure, respectively). They confirmed the similar conditions in the operando PEPICO experiments to the kinetic tests, closing the gap between standard testing and operando measurements. Consistently, no significant deactivation was observed during the measurements. Blank measurements conducted using an empty reactor confirmed the absence of products in both reactions (Supplementary Figs. 12 and 13).

The near-ambient-pressure measurements confirmed the expected formation of major product groups, such as ethylene (C_2_H_4_), propylene (C_3_H_6_), BTX and other C_4+_ hydrocarbons, including C_4_H_8_ (*m*/*z* 56), C_5_H_6_ (*m*/*z* 66), C_5_H_8_ (*m*/*z* 68), C_5_H_10_ (*m*/*z* 70), C_6_H_8_ (*m*/*z* 80) and C_7_H_10_ (*m*/*z* 94) (Fig. 3; Supplementary Figs. 4–11). Extraction of the ms-TPE spectra showed that each of these compounds was present as a complex mixture of isomers, with varying degrees of branching and cyclization (Fig. 4; Supplementary Figs. 19 and 20). Oxygenates, including dimethyl ether ((CH_3_)_2_O; *m*/*z* 46), formaldehyde (CH_2_O; *m*/*z* 30) and acetaldehyde (CH_3_CHO; *m*/*z* 44) were detected in the MTH process (Figs. 3 and 4; Supplementary Figs. 5, 9 and 17). The most notable distinction in low-pressure experiments was the detection of a readily identifiable signal associated with methyl radicals (CH_3_^•^; *m*/*z* 15) and of their characteristic vibrational fingerprints in the corresponding ms-TPE spectra (Fig. 3; Supplementary Figs. 9, 11 and 16). The reduced pressure suppresses the quenching of this highly reactive species, enabling its observation. This experimentally confirms the presence of CH_3_^•^ species in both MTH and MCTH reactions. The detection of CH_3_^•^ radicals also at near-ambient pressure demonstrates that these species are reactive intermediates under realistic conditions rather than just minor desorption by-products from surface methoxy species or mere spectators (Fig. 3; Supplementary Fig. 18).

Signal integration at suitable photon energies enabled quantification of the relative abundance of distinct species as a function of temperature (Fig. 3; Supplementary Figs. 14–17). The increased generation of (CH_3_)_2_O in the MTH reaction and the higher olefin/BTX ratio in both reactions with decreasing conversion agrees well with the kinetic experiments. While BTX increased in the investigated temperature window in the near-ambient pressure experiments, the C_3_H_6_, C_4_H_8_ and C_5_H_10_ signal intensities reached a maximum at approximately 673 K, and the evolution of C_5_H_8_, C_5_H_6_, C_6_H_8_ and C_7_H_10_ peaked at around 723 K in both the MTH and MCTH reactions (Fig. 3; Supplementary Fig. 14). These observations support the presence of a consecutive mechanism in both reactions, where C_5_ compounds are central for chain growth, in line with previous hypotheses^31^. Conversely, the (CH_3_)_2_O signal decreased sharply with increasing temperature in the MTH process, while that of CH_3_CHO followed a volcano behaviour (Fig. 3). These results confirm the generation of (CH_3_)_2_O in the early stages of the MTH process via CH_3_OH dehydration, whereas CH_3_CHO is probably involved consecutively in the formation of the first C–C bond. By contrast, the formation of CH_2_O rose continuously with temperature. The same trend was observed in the low-pressure experiments in the MTH reaction, although the amount of CH_2_O generated was considerably higher (Fig. 3; Supplementary Fig. 17). This suggests that CH_2_O is predominantly involved in the first steps of the hydrocarbon evolution and not in the generation of coke precursors, in agreement with recent studies^19,20^.

On the basis of these findings, two main reaction pathways appear to be responsible for the first C–C bond formation and the subsequent chain growth to C_5_ compounds. The first involves the evolution of oxygenates, such as (CH_3_)_2_O and CH_3_CHO, and is only relevant in the MTH process (Fig. 4a), whereas the second encompasses olefins and occurs in both MTH and MCTH processes (Fig. 4b). In the oxygenate-driven mechanism, CH_3_OH probably initially undergoes dehydration to generate (CH_3_)_2_O. Calculations have shown that both CH_3_OH and (CH_3_)_2_O can react to CH_2_O, releasing H_2_ and CH_4_, respectively^17,43,44^. CH_2_O can be oxidized to carbon monoxide (CO) as demonstrated experimentally and theoretically^15,17^. Consistent with previous literature reports, carbon monoxide can react with adsorbed CH_3_OH, (CH_3_)_2_O or CH_2_O via Koch carbonylation to yield the ketene ethenone (C_2_H_2_O; *m*/*z* 42)^25^, which is assigned by its ionization transition at 9.6 eV in the *m*/*z* 42 ms-TPE spectrum (Fig. 4a). Although this intermediate had been previously predicted^45^, our data provide the experimental evidence of its formation and role in forming the first C–C bond in the MTH reaction. Its detection is highly challenging due to its high reactivity and the low activation barrier (≤17 kJ mol^−1^) for its coordination with Brønsted acid sites, generating readily observable surface-bound acetate or methyl acetate species^1,19,23,45^. In addition, C_2_H_2_O has the same integer mass as C_3_H_6_, which convolutes its identification solely via *m*/*z* analysis. The detection of gas-phase ketene strongly exemplifies the ability of operando PEPICO to discriminate reactive intermediates, either formed or released in the gas phase, from strongly bound species detected using conventional spectroscopic approaches, which are more likely to be spectators in the reaction mechanism. Reproportionation of C_2_H_2_O with CH_3_OH could yield CH_3_CHO and further CH_2_O, whose ionization transitions were detected via photoelectron analysis (Fig. 4a). CH_3_CHO may undergo aldol condensation to form crotonaldehyde (C_4_H_6_O; *m*/*z* 70), which has been discussed in the literature^21^. The unambiguous assignment of the crotonaldehyde vibrational fingerprints was not possible in this case, because it has the same integer mass as C_5_H_10_, an abundant and isomer-complex C_5_ species. Upon methylation and dehydration, this aldehyde may generate 1,3-pentadiene (C_5_H_8_; *m*/*z* 68) (Fig. 4a). Notably, CH_2_O can also react with CH_3_^•^ to form CH_3_CHO. This represents another pathway to C–C bond formation, which is thermodynamically favoured compared with reported methane–formaldehyde (CH_4_–CH_2_O) mechanisms^1^.

In the second pathway, CH_3_X can dissociatively adsorb to form a CH_3_^•^ radical and a coordinated leaving group, X* = Cl* or OH* (Fig. 4b). The creation of the first C–C bond then occurs via the reaction of two CH_3_^•^ radicals to yield C_2_H_4_ upon dehydrogenation, analogous to the proposed direct coupling of surface methoxy species to form this olefin^1^. Chain growth proceeds via the addition of CH_3_^•^ radicals, resulting in a distribution of C_3_H_6_, C_4_H_8_ and C_5_H_10_ isomers (Fig. 4b), explaining the complex ms-TPE spectra observed for C_4_H_8_ and C_5_H_10_ (Supplementary Fig. 19). Interestingly, the signals of isomerized and non-primary olefins were found to be stronger, which is in line with their higher thermodynamic stability compared with primary olefins. CH_4_ is formed via the hydrogenation of CH_3_^•^, whereas the CH_3_^•^ radical–radical reaction results in C_2_H_6_, both products of the MTH and MCTH processes.

The different C_5_H_8_ isomers, which form either via the ketene-driven route in the MTH process or via hydrogen abstraction from C_5_H_10_ in the MTH and MCTH reactions, can undergo further cyclization/dehydrogenation and methylation to generate cyclopentadiene (C_5_H_6_; *m*/*z* 66) and methyl cyclopentadiene (C_6_H_8_; *m*/*z* 80), respectively, in both reactions (Fig. 4c; Supplementary Fig. 20). The detection of (poly)methylated cyclopentadienes corroborates studies that have discussed their involvement in the MTH mechanism and their potential role as further methylating agents^1,46^. Presumably, methyl-radical-driven and regular (that is, based on (poly)methylcyclopentadienes)^46^ methylation paths occur in parallel. Although it is not possible to distinguish the relative contributions of the two routes, due to their high reactivity, it is likely that CH_3_^•^ radical species play an important role in the early stages of the reaction, and that once HCP species form they react indiscriminately in all steps. Photoelectron analysis detects the dehydrogenation product of methylcyclopentadienes (C_6_H_8_), that is, fulvene (C_6_H_6_; *m*/*z* 78), which generates benzene, the first aromatic ring compound, in a subsequent isomerization step (Fig. 4c; Supplementary Fig. 20). Direct methylation of fulvene as well as dehydrogenation–methylation of C_6_H_8_ yields methyl fulvene (C_7_H_8_; *m*/*z* 92), the principal precursor of toluene (Fig. 4c; Supplementary Fig. 20). This mechanism continues to generate other alkylated benzenes, such as isomers of xylene and of trimethylbenzene (Supplementary Fig. 21). Vibrational transitions in the ms-TPE spectra of *m*/*z* 106 could also be assigned to ethylbenzene, which can undergo cracking to yield C_2_H_4_ and C_6_H_6_, representing an additional source of olefins from the autocatalytic HCP pathway^27^. To gain further insight into this route, the following experiment was performed: after running the reaction under identical conditions to those reported in Fig. 3, the flow of reactants was stopped, the reactor flushed with argon (Ar) and the temperature increased while analysing the species evolved. C_2_H_4_ and C_6_H_6_ were observed throughout the tests in both the MTH and MCTH processes, while C_3_H_6_ was found to be almost negligible (Supplementary Fig. 23). This is in line with the occurrence of a second pathway to C_2_H_4_ and C_6_H_6_ that involves the cracking of alkylated benzenes^1^.

### Coke generation and evolution

Further growth of the carbon skeleton leads to the formation of heavy carbonaceous species and ultimately to their deposition as coke in the zeolite pore network^1,10,29,47^. Understanding the generation and evolution of coke is essential to design efficient and robust catalytic technologies for converting C_1_ platform molecules into fine chemicals and fuels. To achieve this, we have combined operando PEPICO with EPR analyses, including continuous wave (CW), pulsed and two-dimensional hyperfine sublevel correlation (2D HYSCORE) measurements. By conducting operando PEPICO, a series of C_10+_ hydrocarbons were observed in both the MTH and MCTH processes at 0.5 bar (Fig. 5a). These species remained virtually unobserved in the low-pressure experiments, indicative of reduced chain-propagation and condensation-reaction rates. We identify two pathways to naphthalene (C_10_H_8_; *m*/*z* 128), which is considered to be the main precursor of heavier carbonaceous species (Fig. 5b). The first one involves a Diels–Alder dimerization of cyclopentadiene into dicyclopentadiene (C_10_H_12_; *m*/*z* 132), which isomerizes to 1,2,3,4- and 1,4,5,8-tetrahydronaphthalene, as observed in the MTH reaction. The latter species can undergo hydrogen abstraction to form dihydronaphthalene and ultimately naphthalene, as identified in both MTH and MCTH processes via ms-TPE spectrum analysis (Fig. 5b; Supplementary Fig. 22). This mechanism was first revealed in detail in the catalytic pyrolysis of benzenediols over H-ZSM-5 (ref. ^48^). In addition, dicyclopentadiene can hydrogenate and isomerize to adamantine, which can form diamantane, a known coke precursor^49^. The second pathway encompasses the reaction of benzene with propyne (C_3_H_4_; *m*/*z* 40) to yield indene (C_9_H_8_; *m*/*z* 116). This agrees with previous studies on ethanol coupling over H-ZSM-5 that have shown the generation of indene-like species using solvent extraction^39^. Further reaction with CH_3_^•^ radicals generates naphthalene (Fig. 5b; Supplementary Fig. 22), following a known mechanism^50^. Interestingly, by comparing the ms-TPE spectra of *m*/*z* 132 photoions, the relative ratio of dicyclopentadiene and tetrahydronaphthalenes was lower in MCTH than in the MTH process (Fig. 5b; Supplementary Fig. 22). A possible explanation for this behaviour is the observed favoured production of aromatics in the MCTH reaction that could promote the indene-driven mechanism. Once naphthalene forms, it can undergo consecutive methylation to yield alkylated naphthalenes (Fig. 5a,b; Supplementary Fig. 22), which has been associated with the generation of heavy polyaromatic compounds^26,49^. This C_1_-addition mechanism agrees with experimental observations and kinetic models^51,52^. When switching off the feed of the MTH and MCTH reactions, the evolution of heavy species was also observed (Supplementary Fig. 23). In particular, photoions associated with naphthalene were detected after both MTH and MCTH processes, while indene and anthracene could also be identified to be released after the MCTH reaction. This is in line with the favoured generation of indene during the MCTH reaction as well as the higher rate of coke accumulation during the first 60 h on stream in the long-term tests.

The evolution of coke species was monitored by studying deposited paramagnetic carbonaceous species via EPR spectroscopy after selected times on stream in MTH and MCTH (Fig. 5c–h). By conducting CW EPR, a signal with an isotropic *g* factor of 2.003 was observed for all investigated samples (Supplementary Fig. 24). By applying a reported method for spin counting^53^, it was possible to quantify the specific amount of radicals formed during the reaction by comparing the double integrals of the EPR signals with a known standard. Accordingly, the average concentration of EPR-active carbonaceous species increased as a function of the time on stream, which is consistent with the coke contents determined via TGA, reaching approximately 4.5 and 7 μmol cm^−3^ after 120 h in the MTH and MCTH reactions, respectively (Fig. 5c). As detailed in Supplementary Note 2, information on the density of these species was obtained by extracting the Gaussian and Lorentzian lineshape contributions via least-squares fitting of the normalized CW EPR spectra (Fig. 5d, inset; Supplementary Figs. 24 and 25). Whereas isolated paramagnetic coke generally results in a Gaussian lineshape, the magnetic interactions of closely packed spins typically yield a Lorentzian-type linewidth. Accordingly, the MCTH reaction resulted in the formation of highly dense carbonaceous compounds within the first 5 h of reaction, whereas the density of these species grew slowly in the MTH reaction (Fig. 5d), consistent with the higher tendency of CH_3_Cl to generate BTX.

Pulsed relaxation EPR measurements, that is, recording the signal decay after microwave pulsing, were performed to further gain insight into the spatial distribution of the detected paramagnetic species (Supplementary Fig. 26). Through least-squares fitting of these decay data with a stretched exponential, the corresponding fractal dimension, *N*, can be extracted (Fig. 5e, inset). Generation of three-dimensional (3D) deposits (*N* ≥ 2.8) was observed after 5 h in the MCTH reaction, whereas *N* increased slowly in the MTH process, reaching 2.7 after 120 h on stream (Fig. 5e). These results indicate that highly packed carbonaceous species form rapidly in the MCTH process and equally along the three dimensions within the zeolite pore network. By contrast, the MTH reaction generates less-dense carbon deposits that grow more slowly, which is in line with the observations gathered using CW EPR, TGA and sorption analysis. Finally, 2D HYSCORE analyses provide further insights into the nature of these deposits (Fig. 5f,g; Supplementary Figs. 27–31). As detailed in Supplementary Note 2, comparison with density functional theory-based simulations and literature data enables the assignment of representative molecular structures to the recorded hyperfine couplings. Marked differences were observed after 2 h on stream in the MTH and MCTH processes, which showed the formation of ethylnaphthalene- and pentacene-like compounds, respectively (Fig. 5f,g). These results are in line with the PEPICO experiments performed by switching off the feed, in which naphthalene in both MTH and MCTH together with indene and anthracene in MCTH were evolved from the H-ZSM-5 catalyst (Supplementary Fig. 23). Notably, ethylnaphthalene was previously observed in ethanol coupling over H-ZSM-5^54^, suggesting that the generation of coke precursors can be strongly influenced by the zeolite framework. Estimation of the corresponding H:C ratio indicates that species with a higher degree of alkylation are formed in the MTH process (Fig. 5h). The presence of the ketene-driven chain-growth route leads to the formation of linear compounds. This, together with the slower initial rate of carbonaceous species generation observed via CW EPR and TGA, can explain the reduced tendency of forming highly aromatic compounds in the MTH process compared with the MCTH reaction. The nature of the formed deposits converges with time on stream (Fig. 5h), and the representative molecular structure corresponds to alkylated coronenes and tribenzocoronenes after 60 and 120 h in both reactions (Supplementary Figs. 28 and 29).

## Discussion

The mechanism of C–C bond formation and chain propagation in the zeolite-catalysed coupling of CH_3_OH and CH_3_Cl was assessed using operando PEPICO spectroscopy, whose unique sensitivity enabled us to quantitatively and isomer-selectively map the generation of intermediates during the early stages of the MTH and MCTH processes. In addition, the strategy of jointly studying the activation of CH_3_OH and CH_3_Cl was crucial for decoupling and discriminating the oxygenate- and hydrocarbon-driven mechanisms. These results provide experimental evidence for the generation of CH_3_^•^ radicals in both reactions and of C_2_H_2_O in the MTH process. Accordingly, two main C–C bond formation and propagation pathways are identified. The first, which is dominant in the MCTH process, involves the reaction of two CH_3_^•^ radicals to generate C_2_H_4_ upon hydrogen transfer, which can undergo further methylation to yield complex isomer mixtures of C_3_–C_5_ hydrocarbons. The second occurs only in the MTH process and encompasses the CH_3_OH- and (CH_3_)_2_O-driven formation of CH_2_O, which, after oxidation into CO, can undergo Koch carbonylation to generate C_2_H_2_O, representing the first C–C bond generation in the MTH process. This ketene can reproportionate in the presence of CH_3_OH to yield CH_2_O and CH_3_CHO. The latter can undergo aldol condensation into crotonaldehyde, which, upon methylation and dehydration, generates linear C_5_H_8_. Interestingly, CH_2_O can react with a CH_3_^•^ radical to generate CH_3_CHO, which is thermodynamically favoured compared with proposed CH_4_–CH_2_O routes and represents an additional subroute for formation of the first C–C bond in the MTH reaction^1^. Both envisioned pathways converge to generate linear and branched C_5_ hydrocarbons, such as C_5_H_10_ and C_5_H_8_. Following a series of methylation, cyclization and hydrogen-transfer reactions, these compounds form fulvene and methyl fulvenes that isomerize to yield benzene and toluene, respectively.

Operando PEPICO spectroscopy also provided insights into the mechanisms for the generation of coke precursors, such as naphthalene and its methylated analogues, in MTH and MCTH. The first involves Diels–Alder dimerization, isomerization and hydrogen abstraction of cyclopentadiene, whereas the second encompasses coupling between propyne and benzene to yield indene, which forms naphthalene upon methylation. These results were further complemented with EPR measurements that shed light on the density, spatial distribution and representative molecular structure of the carbonaceous species deposited in the zeolite micropores during the MTH and MCTH reactions. By combining CW and pulsed EPR with 2D HYSCORE measurements, it could be observed that the MCTH process results in the fast generation of high-density, three-dimensional and low-alkylated carbon deposits, whereas carbonaceous species grow more slowly and form highly alkylated aromatics in the MTH process, particularly after a short time on stream. However, the degree of alkylation of these deposits gradually decreased with time on stream in the MTH reaction until it converged with that obtained in the MCTH reaction after around 60 h.

Determining the different pathways of C–C bond formation and chain propagation that culminate in the generation of carbonaceous species has important practical implications for the design of catalytic and reactor systems for CH_3_OH and CH_3_Cl coupling, and should favour oxygenate-driven routes to hinder the formation of dense polyaromatic species for developing efficient and robust catalytic technologies. On the basis of the map of reactive intermediates obtained via the operando analysis in this work, we have proposed a specific reactivity map for those intermediates; further studies will undoubtedly provide additional insight that will help to refine further our understanding of the actual reaction network. Going forward, these techniques provide a valuable platform, potentially in combination with co-feeding experiments, for gaining insights into other long-standing questions in hydrocarbon transformations, for example, to confirm the effects of promoters or reactant leaving groups, distinguishing mere spectators from real reaction intermediates, and could be applied to virtually all hydrocarbon functionalization processes.

## Methods

### Catalyst synthesis and basic characterization

A commercial ZSM-5 zeolite (CBV 8014 from Zeolyst International, nominal Si/Al = 40, ammonium form) was calcined at 823 K (5 K min^−1^) for 5 h to obtain the protonic form. The crystalline order, porous and acidic properties, and the presence of coke in the fresh and used catalysts were characterized using powder X-ray diffraction, gas sorption at 77 K, Fourier transform infrared spectroscopy of adsorbed pyridine, and TGA, respectively. Full details are provided in the Supplementary Methods.

### Catalytic evaluation

Methanol and methyl chloride coupling reactions were performed at ambient pressure using a continuous-flow fixed-bed reactor setup (Supplementary Fig. 32). In a typical test, the zeolite catalyst (catalyst weight, *W*_cat_ = 0.6 g) was loaded in a quartz microreactor and pre-treated at 673 K for 1 h before switching to the desired reaction temperature. Thereafter, a total gas flow, *F*_T_ = 20 cm^3^ STP min^−1^, containing 50 vol% of CH_3_X (X = OH or Cl) in helium, was fed into the reactor at the desired reaction temperature (*T* = 523–723 K). Quantification of the reactant conversion and product selectivity, as well as the error in the carbon mass balance, was conducted according to the protocols described in the Supplementary Methods.

### Operando photoelectron photoion coincidence spectroscopy

PEPICO experiments were performed using the CRF-PEPICO endstation at the vacuum ultraviolet beamline of the Swiss Light Source of the Paul Scherrer Institute, Switzerland^55–57^. The unique reactor configuration extends the lifetime of elusive reaction intermediates, thanks to the high dilution of the feed, the low pressure inside the reactor (around 0.05–0.5 bar) and the free jet molecular beam expansion, enabling their detection (Supplementary Fig. 33). Full details of the protocol are provided in the Supplementary Methods.

### Electron paramagnetic resonance spectroscopy

The evolution of coke with the time on stream was monitored using ex situ EPR spectroscopy. A set of experiments, including CW, pulsed and 2D HYSCORE measurements, was carried out to investigate the distinctive features of the deposited carbonaceous species, such as the density, spatial distribution and representative molecular structure (Supplementary Fig. 34). Full details of the protocol are provided in the Supplementary Methods.

## Supplementary information

Supplementary Information Supplementary Notes 1 and 2, Methods, Tables 1–3, Figs. 1–34 and references.

## Data Availability

Data presented in the main figures of the manuscript are publicly available through the Zenodo repository (10.5281/zenodo.6547649). Further data supporting the findings of this study are available in the Supplementary Information. All other relevant source data are available from the corresponding author upon request. Source data are provided with this paper.

## Source data

Source Data Fig. 2 Statistical source data.
Source Data Fig. 3 Statistical source data.
Source Data Fig. 4 Statistical source data.
Source Data Fig. 5 Statistical source data.
