# Supplementary material for: Elucidation of radical- and oxygenate-driven paths in zeolite-catalysed conversion of methanol and methyl chloride to hydrocarbons
Source: Nat Catal. 2022 Jun 27;5(7):605–14. doi: 10.1038/s41929-022-00808-0 (PMC7613158; doi:10.1038/s41929-022-00808-0)
Supplement: Supplementary file 1 — Supplementary Notes 1 and 2, Methods, Tables 1–3, Figs. 1–34 and references. [file 41929_2022_808_MOESM1_ESM.pdf]

nature portfolio

https://doi.org/10.1038/s41929-022-00808-0

Supplementary information

Elucidation of radical- and oxygenate-driven paths in zeolite-catalysed conversion of methanol and methyl chloride to hydrocarbons

In the format provided by the authors and unedited

Supplementary Information

Elucidation of radical- and oxygenate-driven paths in zeolite-catalyzed conversion of methanol and methyl chloride to hydrocarbons

Alessia Cesarini,[1†] Sharon Mitchell,[1†] Guido Zichittella,[1†]* Mikhail Agrachev,[2] Stefan P. Schmid,[2] Gunnar Jeschke,[2] Zeyou Pan,[3] Andras Bodi,[3] Patrick Hemberger,[3]* and Javier Pérez-Ramírez[1]*

[1] Institute for Chemical and Bioengineering, Department of Chemistry and Applied Biosciences, ETH Zurich, Vladimir-Prelog-Weg 1, 8093 Zurich, Switzerland,

[2] Laboratory of Physical Chemistry, Department of Chemistry and Applied Biosciences, ETH Zurich, Vladimir-Prelog-Weg 2, 8093 Zurich, Switzerland,

[3] Laboratory of Synchrotron Radiation and Femtochemistry, Paul Scherrer Institute, 5232 Villigen PSI, Switzerland,

[†]These authors contributed equally, listed alphabetically.

*Corresponding author. E-mails: zguido@mit.edu, patrick.hemberger@psi.ch, jpr@chem.ethz.ch.

Table of contents

Supplementary Note 1 2

Supplementary Tables 6

Supplementary Tables 1-3 6

Supplementary Figures 8

Kinetic analysis and basic characterization 8

Supplementary Figures 1-3 8

Near-ambient-pressure operando PEPICO experiments 11

Supplementary Figures 4-7 11

Low-pressure operando PEPICO experiments 15

Supplementary Figures 8-11 15

Blank operando PEPICO experiments 19

Supplementary Figures 12,13 19

Product evolution by operando PEPICO 21

Supplementary Figures 14-17 21

Isomer analysis by operando PEPICO 25

Supplementary Figures 18-22 25

Desorption of chemical species under inert atmosphere 30

Supplementary Figure 23 30

Density of coke deposits by cw EPR 31

Supplementary Figures 24,25 31

Coke fractal dimension by pulsed EPR 33

Supplementary Figure 26 33

Representative molecular structure of carbonaceous deposits derived by 2D HYSCORE 34

Supplementary Figures 27-31 34

Supplementary Note 2 39

Supplementary methods 43

Catalyst characterization 43

Catalyst evaluation 43

Supplementary Scheme 1 45

Operando photoelectron photoion coincidence spectroscopy 46

Supplementary Scheme 2 49

Electron paramagnetic resonance spectroscopy 50

Supplementary Scheme 3 52

Supplementary references 53

Supplementary Note 1

Approaches to understand the mechanism of ZSM-5 catalyzed methanol and methyl chloride conversion and outstanding questions.

Since the introduction of the catalytic process of converting methanol to hydrocarbons over ZSM-5 as a route from natural gas to synthetic gasoline, extensive research has been conducted to understand the mechanism and optimize the product distribution.[1-4] Here, we briefly summarize previously applied approaches to gain insight into the reaction, key findings, and open questions.

Kinetic studies have provided insights about how the product distribution depends on the zeolite pore topology, acidic properties, and operating conditions. Early data demonstrated the importance of autocatalytic reactions between methanol or dimethylether and olefins, identifying the conversion of oxygenates to hydrocarbons as the rate-limiting step of the process.[5,6] Compelling evidence from experimental studies using pure feeds indicated that olefin formation is unlikely to proceed via the direct coupling of C1 species,[7] which was supported by prohibitively high activation barriers predicted by theoretical studies.[8] The results established that an indirect route involving large organic species adsorbed in the zeolite likely prevails under steady-state conditions.[9,10] In this so-called hydrocarbon pool mechanism, C−C bond formation and subsequent olefin evolution upon addition of methanol proceed via a series of steps involving interactions with species present in the hydrocarbon pool, rather than via the direct coupling of C_1 entities. Several studies have attempted to discriminate pathways within the hydrocarbon pool through measurement of rates and activation energies of individual reaction steps, but translating kinetic data into mechanistic insights remains challenging due to the complexity of the reaction network.[11] The relative contributions of the aromatics- and olefin-based catalytic cycles (see below) to the product distribution have been estimated through co-feeding experiments.[12] The presence of aromatic hydrocarbons in the feed was observed to enhance methanol conversion, providing early evidence for the dual cycle model and the predominance of methylbenzenes in the hydrocarbon pool.[13,14]

Isotope labeling experiments have been particularly useful at corroborating various features of the reaction mechanism. ^{13}C labeling experiments involving the co-reaction of labeled methanol with unlabeled hydrocarbon species and transient studies switching from ^{12}C- to ^{13}C-containing methanol have yielded important information regarding potential intermediate species and primary and secondary product formation.[15] Ethylene was found to be formed mainly from methylbenzenes, and its production is mechanistically distinct from that of higher olefins.[16] The results suggested that the alkylation-cracking route is primarily responsible for the production of C_{3+} olefins.[9] These findings provided the basis for the widely accepted dual aromatic-olefin catalytic cycles.[17] H/D exchange reactions have shown that hydrogen transfer can occur via different mechanisms, including through unimolecular (e.g., formation of adsorbed alkoxy species) or bimolecular reactions.[18] They also confirmed the limited reactivity of framework-bound methoxy groups in methanol conversion.[10] While these methods revealed crucial details on the evolution of condensed aromatic species, they have failed to generate insights on the role of highly reactive intermediates, such as aldehydes and ketenes that are believed key in the formation of the first C−C bond.

This is because archetypical labeling experiments are conducted in standard kinetic reactor setups, which possess medium-low time resolutions. Accordingly, highly reactive species are either quenched or never leave the zeolite, thus requiring more advanced and time-resolved methods to detect these compounds during reaction conditions.

To this end, spectroscopic approaches including nuclear magnetic resonance (NMR), infrared (IR), ultraviolet-visible (UV-vis) and UV-Raman spectroscopies, have been extensively used to detect intermediate species present within the zeolite catalysts.[19] These studies have confirmed the formation of various covalently adsorbed species (e.g., surface methoxy groups) as well as fully protonated carbenium ions (e.g., cyclopentenyl).[20] However, the direct observation of intermediates under operando condition remains a challenge, because of the high reactivity of certain species and limitations in the sensitivity and specificity of typical spectroscopic methods. A specific difficulty is to distinguish stable spectator species, which may be more prone to detection through spectroscopic techniques with limited time resolution or detection thresholds, from active intermediates that may be short-lived and present only in low concentrations. An example that demonstrates this point is our detection of ketene. The latter has been hypothesized and theoretically predicted to play an active role in methanol conversion to hydrocarbons,[21] but has never been detected experimentally previously. The difficulty in confirming ketene formation arises because of its ready equilibration over Brønsted acid sites, generating stable acetate species. These have been spectroscopically observed and are likely spectators in the reaction network.[22]

Thus, despite these dedicated efforts to understand the mechanism, several aspects remain debated:

1. The existence and role of radical species in the reaction. The mechanistic involvement of radicals has been speculated by several studies, mainly in relation to the formation of the first C−C bond.[23] The formation of free radicals was detected in the conversion of dimethyl ether over ZSM-5 by electron paramagnetic resonance (EPR) spectroscopy.[24] Although not directly confirmed, the authors postulated the involvement of methoxymethyl radicals. Subsequent experiments co-feeding nitric oxide as a radical scavenger provided evidence for their potentially complex role, particularly in the early stages of the reaction.[23] More recently, an EPR study evidenced the presence of aromatic radicals in acidic ZSM-5, but it was not possible to confirm their identity.[25] To date, direct experimental confirmation of the formation, types, and contributions of radical species to the reaction remains mostly lacking.

2. Early stages of the reaction mechanism. While the relevance of hydrocarbon pool species for olefin formation in steady-state operation has been demonstrated, their development in the early stages of reaction remains debated and methods that enable the in-situ observation of hydrocarbon evolution remain sought after. New methods to systematically detect all gaseous products, including highly reactive species present in low concentrations, may provide valuable insights to corroborate or negate the numerous mechanistic proposals, for example the bridging role of methycyclopentenes,[26] direct coupling of surface adsorbed C_1 species and release to ethylene,[27] the Koch-type carbonylation through dehydrogenation of methanol/formaldehyde leading to methyl acetate,[28] or the formation of the first olefin via condensation of formaldehyde with acetic acid/methyl acetate,[29] and so on.

3. Coke formation mechanism. Several studies have attempted to resolve the mechanism of coke deposition and the propagation of coke species during the conversion of methanol over zeolite catalysts. Dissolution-extraction experiments have shown that deactivated ZSM-5 catalysts contain polyaromatic species of between 16-35 carbon atoms in size.[30,31] Although these experiments can effectively determine the molecular identity of residual carbonaceous species, it is unclear how the composition of coke species changes from under reaction conditions and they cannot account for volatile species trapped within the pore network. Spectroscopic approaches, e.g., UV-vis,[32] IR, UV-Raman,[33,34] solid-state NMR[35], and EPR[36-39] provide insights on the general chemical nature of coke species (e.g., methylated aromatic compounds or graphite-related systems) but most previous studies have focused on qualitative analysis. Another challenge is to verify how external coke differs from internal coke, polyaromatic hydrocarbons of chain-like rather than sheet-like topology has been reported to form in ZSM-5 zeolites, reflecting the pore architecture.[40,41] Methods that can provide more precise information on the nature of large aromatic species thought to be coke precursors and bridge the gap to graphitized "hard" coke species are of great interest.

4. Similarity to methyl chloride coupling. Compared to methanol-to-hydrocarbons, the mechanism of converting methyl chloride to hydrocarbons (MCTH) has received little attention. Based on the broadly similar product distribution, most previous studies have assumed that the two reactions occur via similar paths.[42] Although there are clear parallels, there are also notable distinctions since intermediates thought to be relevant in the conversion of methanol (e.g., dimethyl ether, formaldehyde, oxygenated coke species, etc.) are not accessible with methyl chloride.[43] An early ^{13}C MAS-NMR study of methyl chloride conversion over Cs-X zeolite suggested that the first C−C bond forms via the coupling of surface methoxy groups, but this has not been confirmed for proton-exchanged zeolite catalysts.[44] Furthermore, the potential involvement of chlorinated species is unclear. A comparative study of the product evolution in the two reactions would be instructive to clarify similarities and differences between the two processes.

As described above, there are still open questions about the reaction mechanism of both MTH and MCTH that could not be verified experimentally. It was precisely the limitations of established techniques that prompted us to explore new approaches to shed new light on the chemistry of these reactions. In particular, operando photoion photoelectron (PEPICO) spectroscopy provides a means to quantify not only the reactant consumption but also enables the isomer-selective detection of intermediates and products desorbed from the zeolite (up to C_{14}). As detailed in the Supplementary Methods, this is possible due to the particular configuration of the PEPICO microreactor and gas sampling setup. While operating at near-ambient pressure (ca. 0.5 bar) inside the reactor allows to gain realistic mechanist insights, the low pressure at the outlet of the reactor and inside the detection chamber (ca. 2×10^{-9} bar, mean free path ca. 10 m) dramatically reduces quenching of reactive intermediates and preserves the chemical identity of the desorbed species. Operando PEPICO can discriminate reactive intermediates, formed or released in the gas phase, from strongly bound species detected by conventional spectroscopic approaches, which remain inside the zeolite pores and are more likely to be spectators in the reaction mechanism. The sensitivity is clearly illustrated by the detection of ketene, as detailed in the main manuscript, which we report here for

the first time. By performing temperature-dependent studies, it is possible to distinguish between primary or secondary intermediates or tertiary products. Operando PEPICO also captures the generation and evolution of coke precursors, such as naphthalenes, which is hard to follow using archetypical dissolution-extraction techniques. EPR provides pivotal complementary insights into the development and chemical nature of carbonaceous species deposited in the zeolite framework, which leads to the unavoidable deactivation of the catalyst at long time-on-streams. Specifically, by combining continuous wave, pulsed, and 2D hyperfine sublevel correlation EPR analysis, distinctive features of the deposited paramagnetic carbonaceous species, such as average concentration, spatial distribution, and representative molecular structure, could be derived. Although previous studies have observed these species, which give rise to a signal with an isotropic g factor of 2.003,[36-39] in hydrocarbon transformations over zeolite catalysts and have identified changes in the linewidth with time on stream, this effect was mostly attributed to the size increase of coke-forming molecules, and a detailed quantitative analysis has not been undertaken.[45-48]

Supplementary Table 1. Acidic properties of the H-ZSM-5 zeolite catalysts in fresh form and after use in MTH and MCTH.

Sample	Time-on-stream (h)	$c_{\text{Brønsted}}$ (μmol g^{-1})[a]	c_{Lewis} (μmol g^{-1})[a]
Fresh	-	221	46
MTH[b]	120	203	50
MCTH[b]	120	81	118

[a] Determined by IR spectroscopy of adsorbed pyridine. [b] Catalysts were calcined at 773 K before measurement to remove carbonaceous species.

Supplementary Table 2. Measurement parameters used during the cw EPR experiments.

Parameters	Value
Microwave frequency (GHz)	9.165
Centre field (mT)	330
Sweep width (mT)	20
Modulation amplitude (mT)	0.05
Modulation frequency (kHz)	100
Microwave power (mW)	0.7
Power attenuation (dB)	20
Conversion time (ms)	327.68
Time constant (ms)	5.12

Supplementary Table 3. Hyperfine interactions of the modeled cationic molecular structures.

Structure	^{1}H nuclei[a]	Isotropic Fermi contact coupling	Anisotropic spin dipole coupling		
		A (MHz)	T_x (MHz)	T_y (MHz)	T_z (MHz)
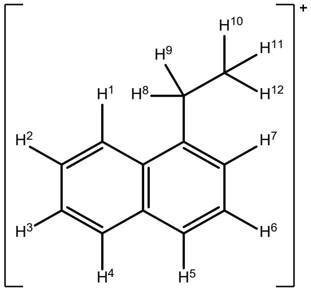	H^{1}	−14.5	−6.5	−1.9	8.4
	H^{2}	−4.8	−3.5	−1.9	5.3
	H^{3}	−3.5	−2.9	−1.9	4.8
	H^{4}	−14.9	−6.7	−2.2	8.9
	H^{5}	−19.3	−9.5	−2.3	11.8
	H^{6}	−4.5	−3.5	−2.3	5.9
	H^{7}	−6.8	−4.5	−2.2	6.7
	H^{8}, H^{9}	27.8	−2.2	−0.5	2.7
	H^{10}	−0.9	−0.7	−0.7	1.4
	H^{11}, H^{12}	−0.7	−1.4	−1.1	2.5
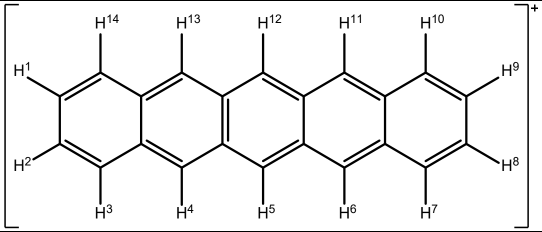	H^{1}, H^{2}, H^{8}, H^{9}	−1.9	−0.5	−0.2	0.7
	H^{3}, H^{7}, H^{10}, H^{14}	−2.8	−1.0	−0.5	1.5
	H^{4}, H^{6}, H^{11}, H^{13}	−12.0	−5.6	−1.2	6.9
	H^{5}, H^{12}	−13.5	−6.0	−1.6	7.6
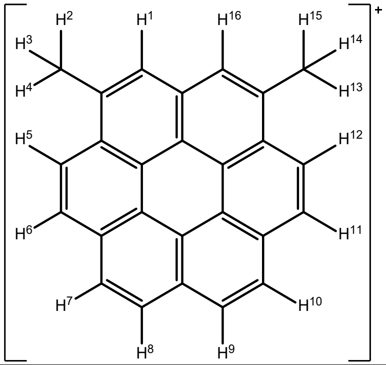	H^{1}, H^{8}, H^{9}, H^{16}	3.3	−1.0	−0.6	1.6
	H^{2}, H^{15}	0.2	−0.9	−0.8	1.6
	H^{3}, H^{4}, H^{13}, H^{14}	13.0	−1.3	−0.5	1.8
	H^{5}, H^{12}	−5.5	−2.7	−1.7	4.4
	H^{6}, H^{11}	−7.0	−3.6	−1.7	5.4
	H^{7}, H^{10}	−8.1	−4.2	−1.3	5.5

[a] The right superscript identifies the ^{1}H nuclei that originate the calculated hyperfine couplings.

Supplementary Figures – Kinetic analysis and basic characterization

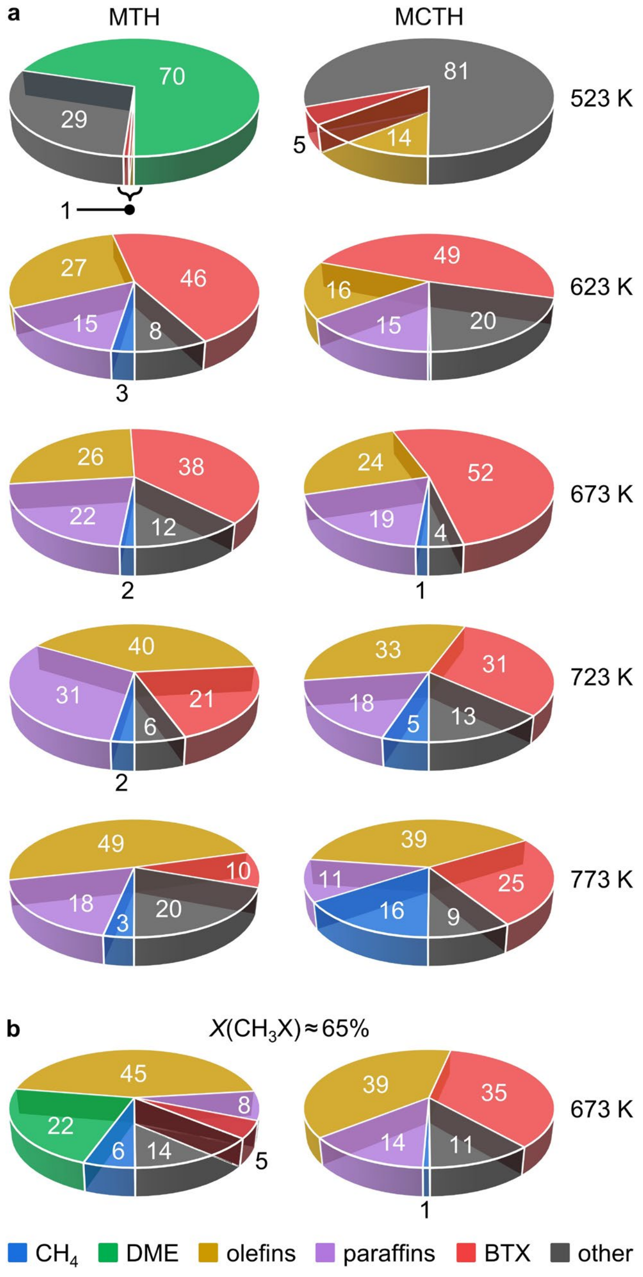

Supplementary Figure 1. Product distribution (in percent) obtained after 5 h on stream in MTH and MCTH over H-ZSM-5 at **a,** different reaction temperatures and at **b,** equivalent conversion. Conditions: **a,** F_T = 20 cm^3 STP min^{-1}, W_{cat} = 0.6 g, T = 523-773 K; **b,** F_T = 400 cm^3 STP min^{-1}, W_{cat} = 0.2 g (MTH); F_T = 20 cm^3 STP min^{-1}, W_{cat} = 0.135 g (MCTH); T = 673 K; **a,b,** CH_3X:He = 1:1, P = 1 bar.

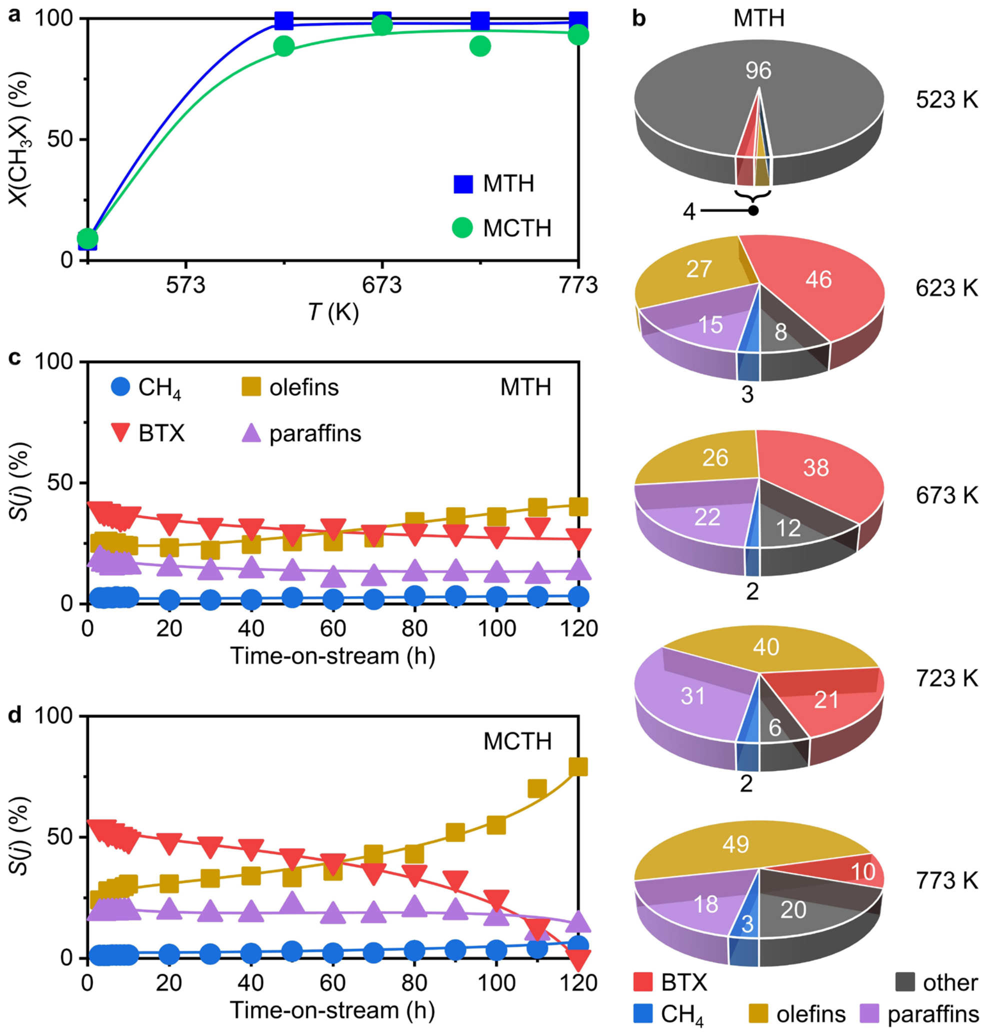

Supplementary Figure 2. a, Reactant conversion and **b,** product distribution (in percent) as a function of temperature in MTH and MCTH over H-ZSM-5. Here, the conversion and selectivity in MTH were determined considering DME as a reactant. However, since no DME was observed when methanol conversion was 100% ($T < 623$ K, see **Fig. 2** of the main manuscript), equivalent product distributions are observed. The most significant difference is the conversion at 523 K drops to a similar value as evidenced in MCTH. Selectivity as a function of time-on-stream in **c,** MTH and **d,** MCTH. Conditions: $F_T = 20$ cm^3 STP min^{-1}, $W_{cat} = 0.6$ g, $T = 523$-773 K CH_3X:He = 1:1, $P = 1$ bar.

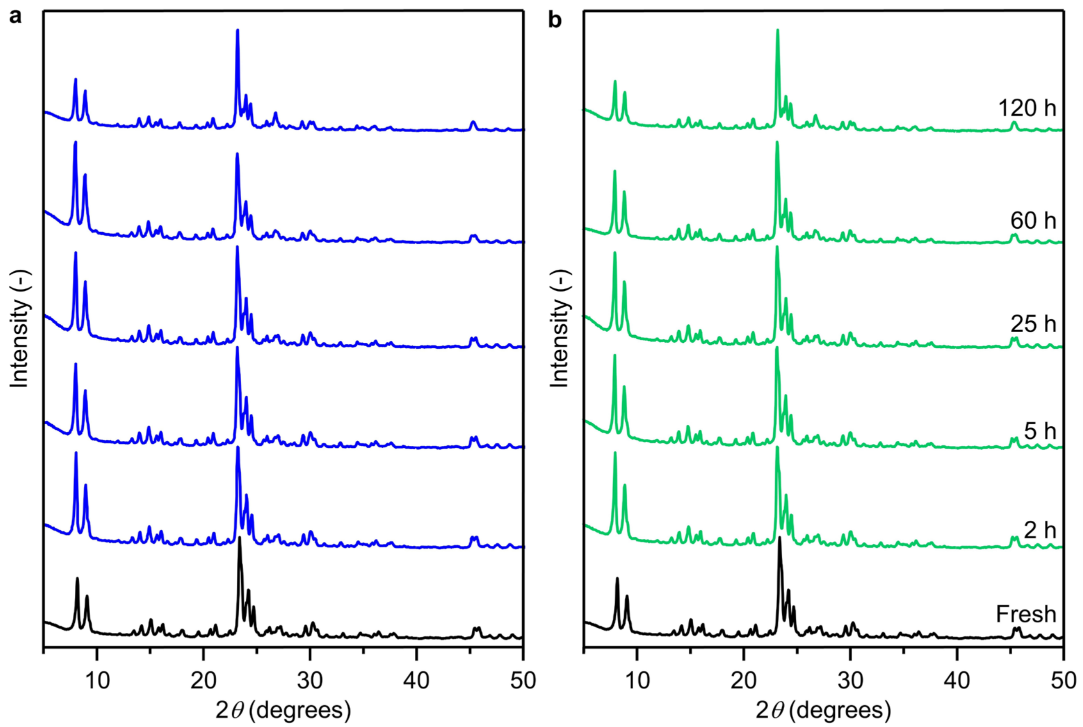

Supplementary Figure 3. X-ray diffraction patterns of the H-ZSM-5 zeolite catalysts in fresh form and after different time-on-stream in **a,** MTH and **b,** MCTH. All used catalysts were calcined before measurement to remove carbonaceous deposits, allowing to compare the crystallinity of the zeolite phase. Conditions: CH_3X:He = 1:1, F_T = 20 cm^3 STP min^{-1}, W_{cat} = 0.6 g, T = 673 K, P = 1 bar.

Supplementary Figures – Near-ambient-pressure operando PEPICO experiments

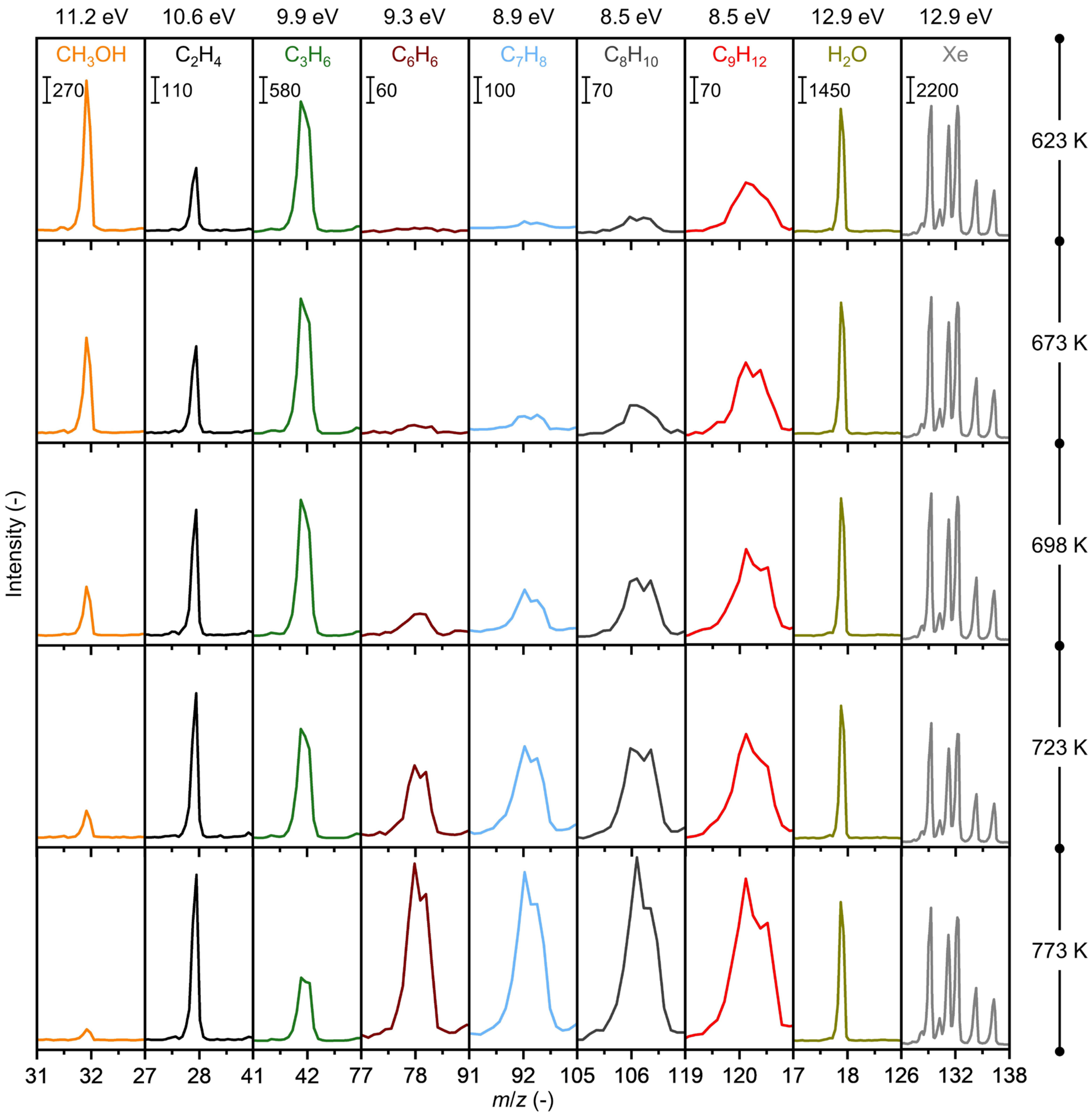

Supplementary Figure 4. Mass spectra of reactants, products, and inert standard at the reactor outlet in MTH over H-ZSM-5 at different temperatures detected by operando PEPICO spectroscopy. The photon energies at which the signals of each chemical species were recorded are given above the plots. The scales of the *y* axes indicated in the top row apply to all spectra in the same column. Conditions: CH_3OH:Xe:Ar = 1:0.1:20.9, F_T = 22 cm^3 STP min^{-1}, W_{cat} = 0.05 g, P = 0.5 bar.

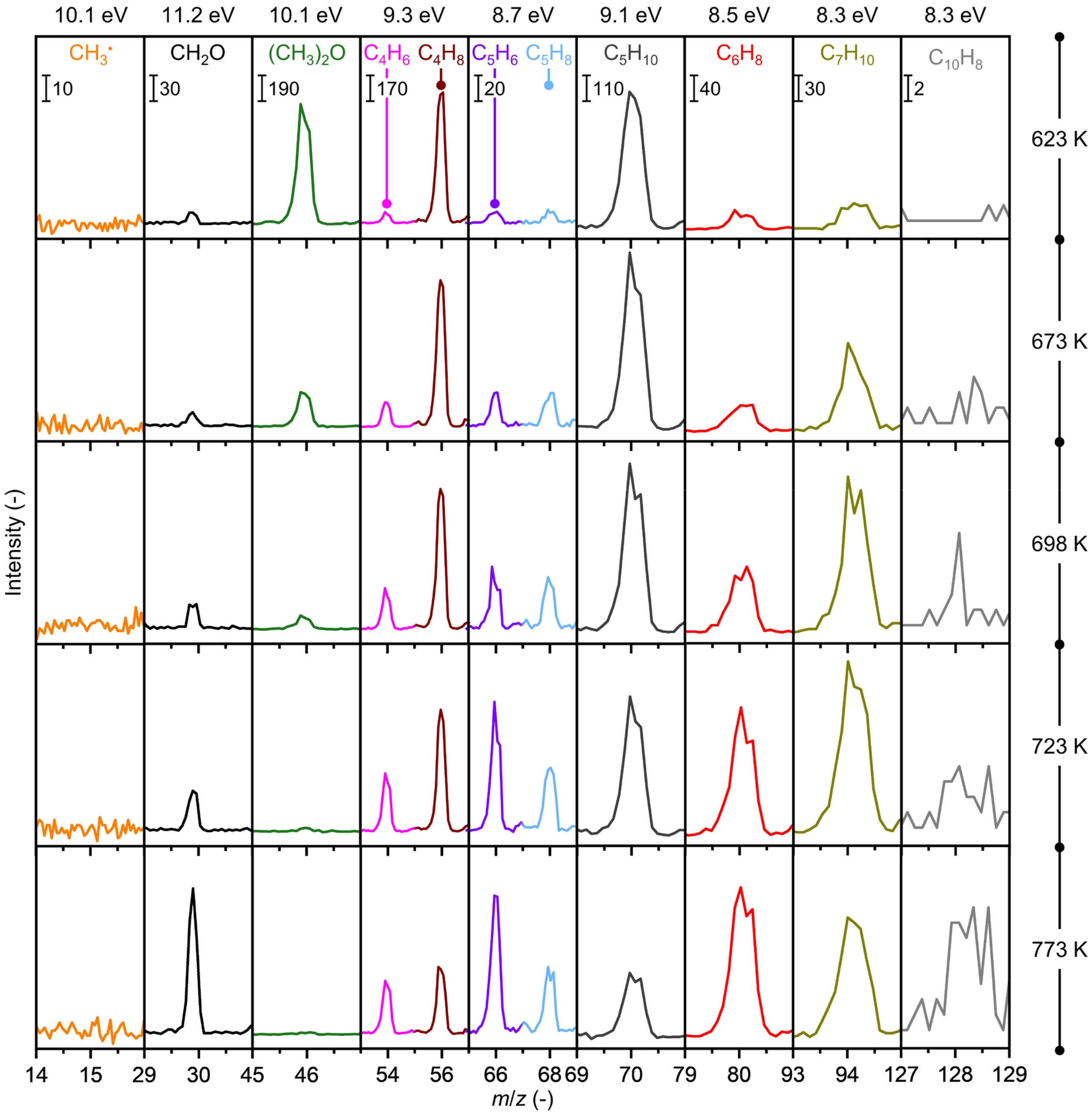

Supplementary Figure 5. Mass spectra of intermediate species at the reactor outlet in MTH over H-ZSM-5 at different temperatures detected by operando PEPICO spectroscopy. The photon energies at which the signals of each chemical species were recorded are given above the plots. The scales of the *y* axes indicated in the top row apply to all spectra in the same column. Conditions: CH_3OH:Xe:Ar = 1:0.1:20.9, F_T = 22 cm^3 STP min^{-1}, W_{cat} = 0.05 g, P = 0.5 bar.

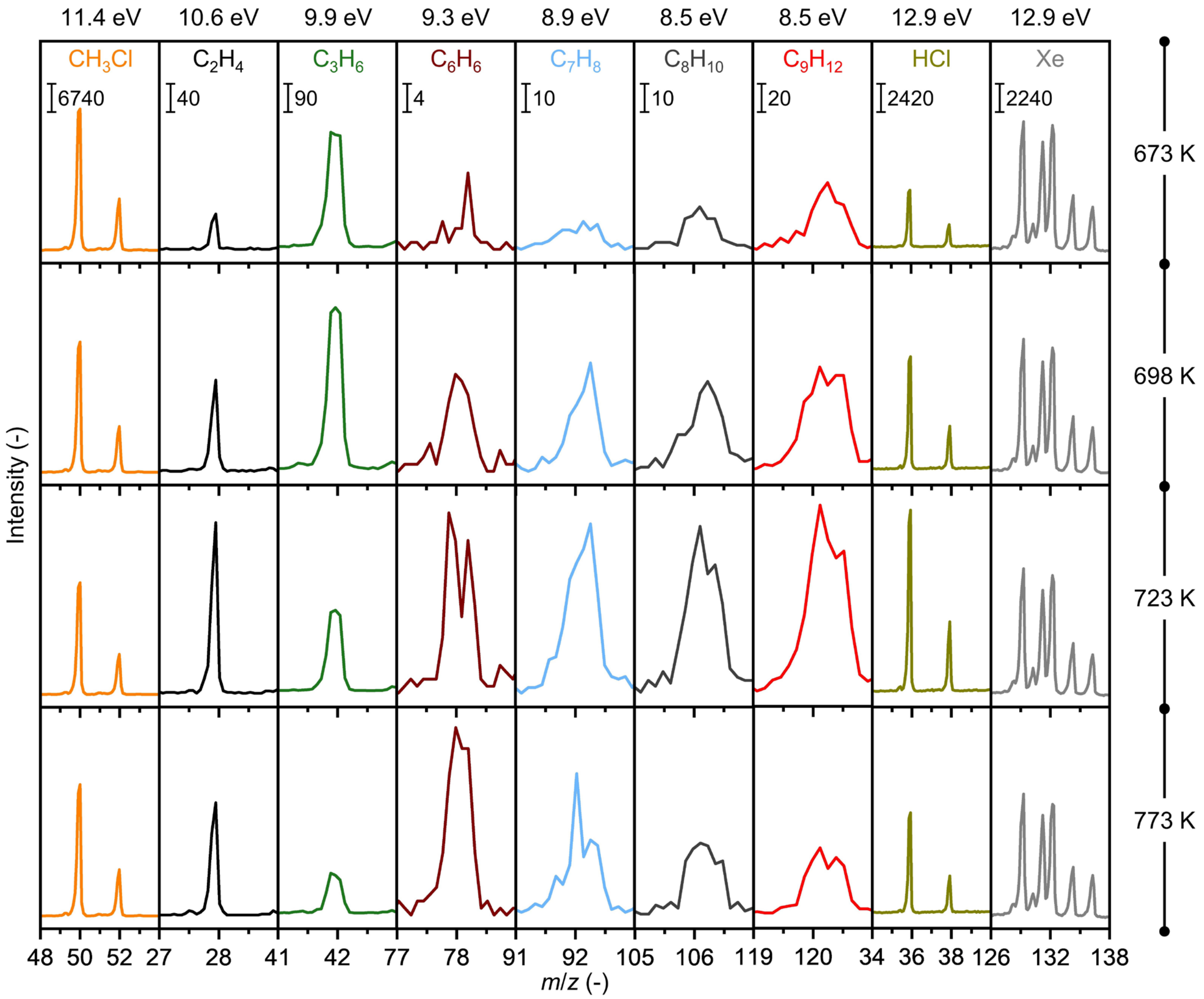

Supplementary Figure 6. Mass spectra of reactants, products, and inert standard at the reactor outlet in MCTH over H-ZSM-5 at different temperatures detected by operando PEPICO spectroscopy. The photon energies at which the signals of each chemical species were recorded are given above the plots. The scales of the *y* axes indicated in the top row apply to all spectra in the same column. Conditions: CH_3Cl:Xe:Ar = 1:0.1:20.9, F_T = 22 cm^3 STP min^{-1}, W_{cat} = 0.05 g, P = 0.5 bar.

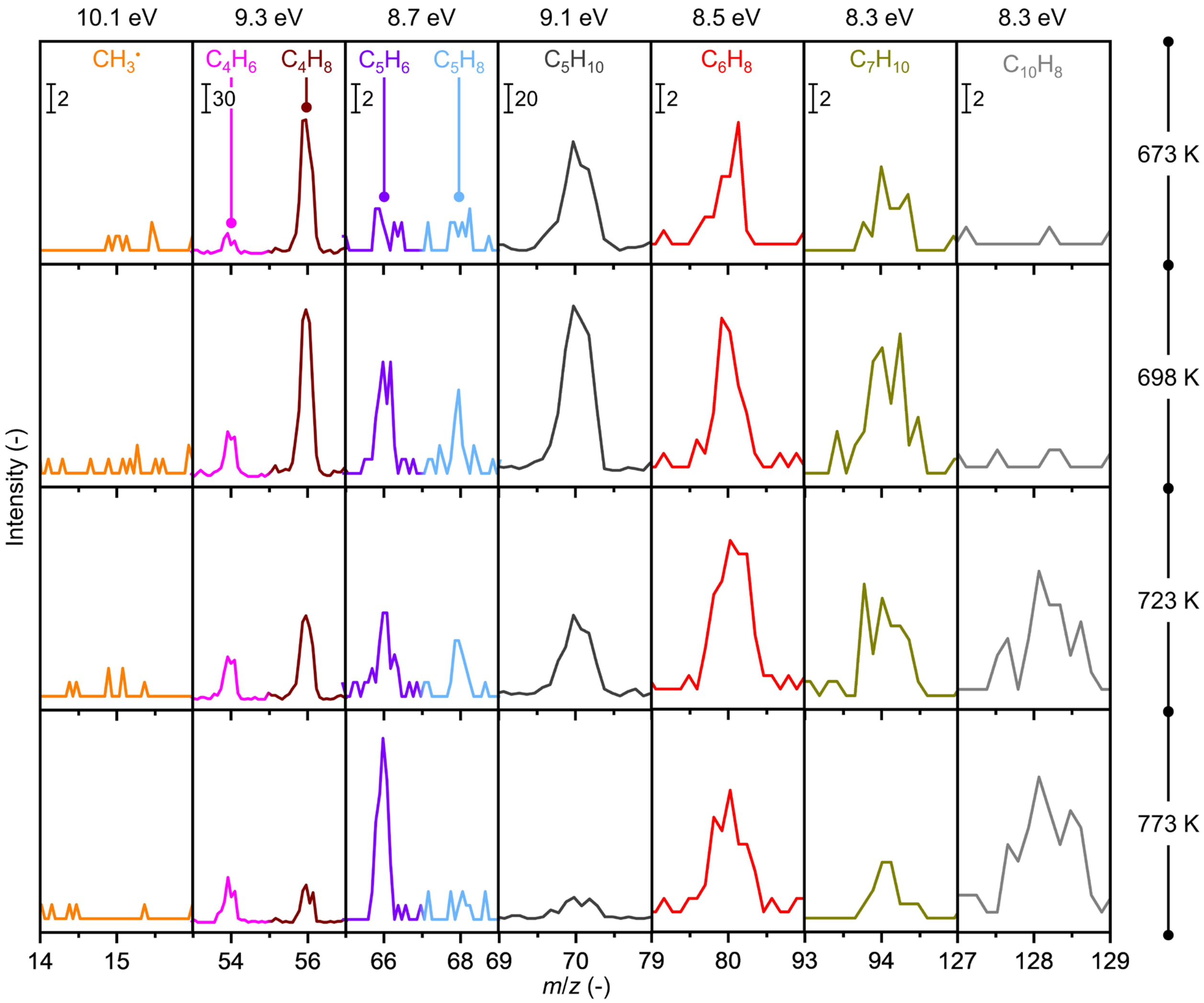

Supplementary Figure 7. Mass spectra of intermediate species at the reactor outlet in MCTH over H-ZSM-5 at different temperatures detected by operando PEPICO spectroscopy. The photon energies at which the signals of each chemical species were recorded are given above the plots. The scales of the *y* axes indicated in the top row apply to all spectra in the same column. Conditions: CH_3Cl:Xe:Ar = 1:0.1:20.9, F_T = 22 cm^3 STP min^{-1}, W_{cat} = 0.05 g, P = 0.5 bar.

Supplementary Figures – Low-pressure operando PEPICO experiments

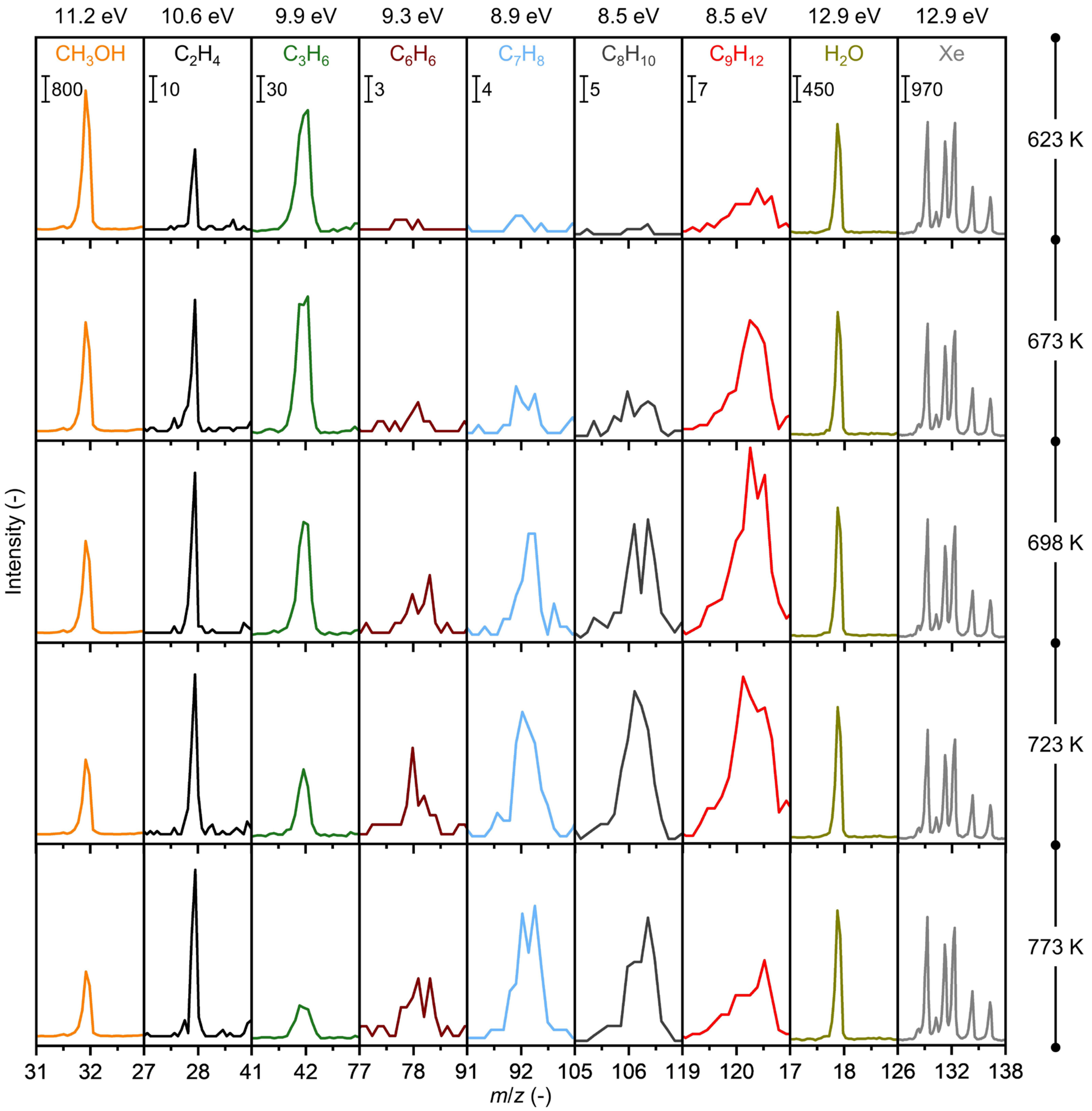

Supplementary Figure 8. Mass spectra of reactants, products, and inert standard at the reactor outlet in MTH over H-ZSM-5 at different temperatures detected by operando PEPICO spectroscopy. The photon energies at which the signals of each chemical species were recorded are given above the plots. The scales of the *y* axes indicated in the top row apply to all spectra in the same column. Conditions: CH_3OH:Xe:Ar = 1:0.1:20.9, F_T = 22 cm^3 STP min^{-1}, W_{cat} = 0.01 g, P = 0.05 bar.

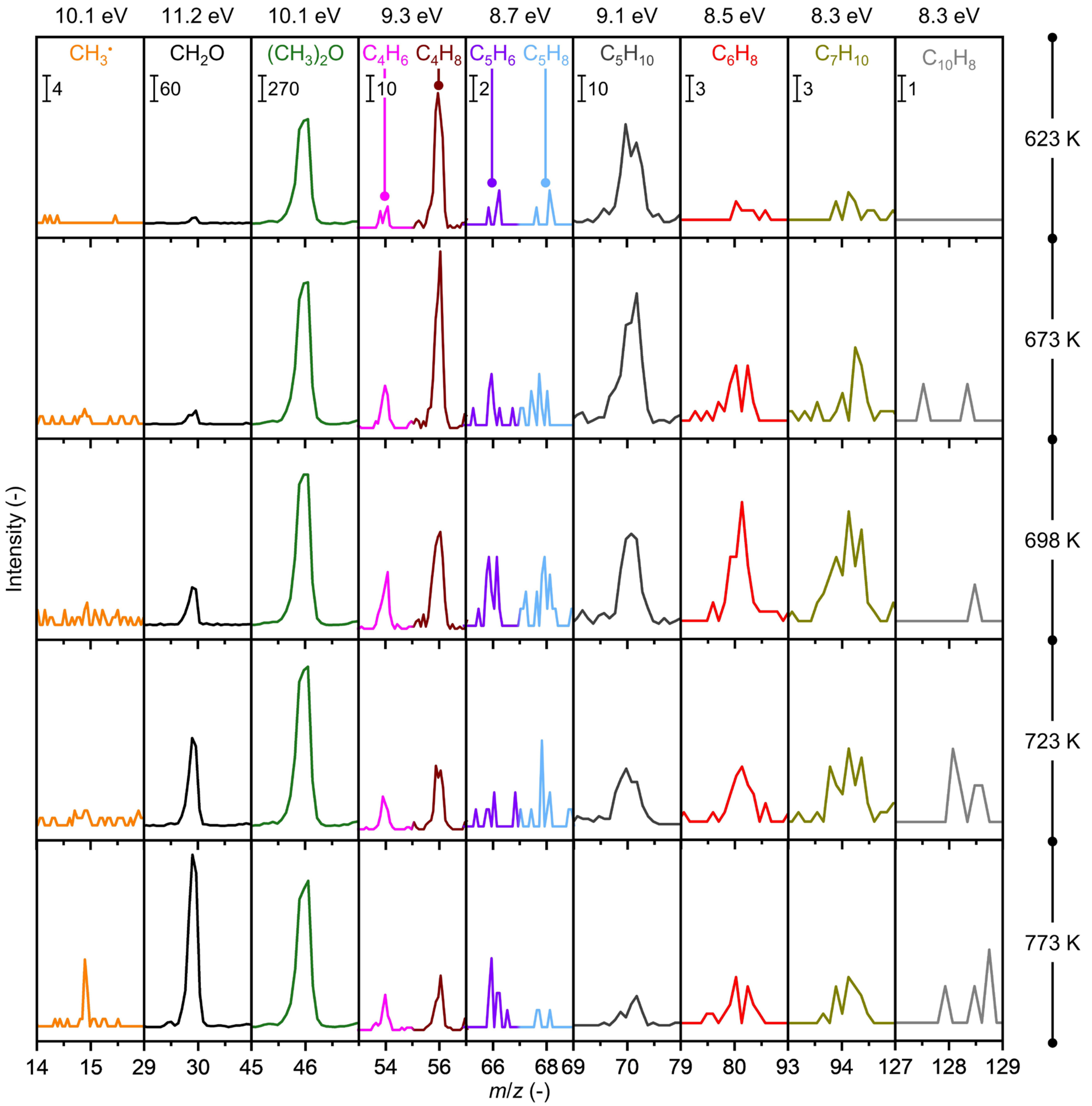

Supplementary Figure 9. Mass spectra of intermediate species at the reactor outlet in MTH over H-ZSM-5 at different temperatures detected by operando PEPICO spectroscopy. The photon energies at which the signals of each chemical species were recorded are given above the plots. The scales of the y axes indicated in the top row apply to all spectra in the same column. Conditions: CH_3OH:Xe:Ar = 1:0.1:20.9, F_T = 22 cm^3 STP min^{-1}, W_{cat} = 0.01 g, P = 0.05 bar.

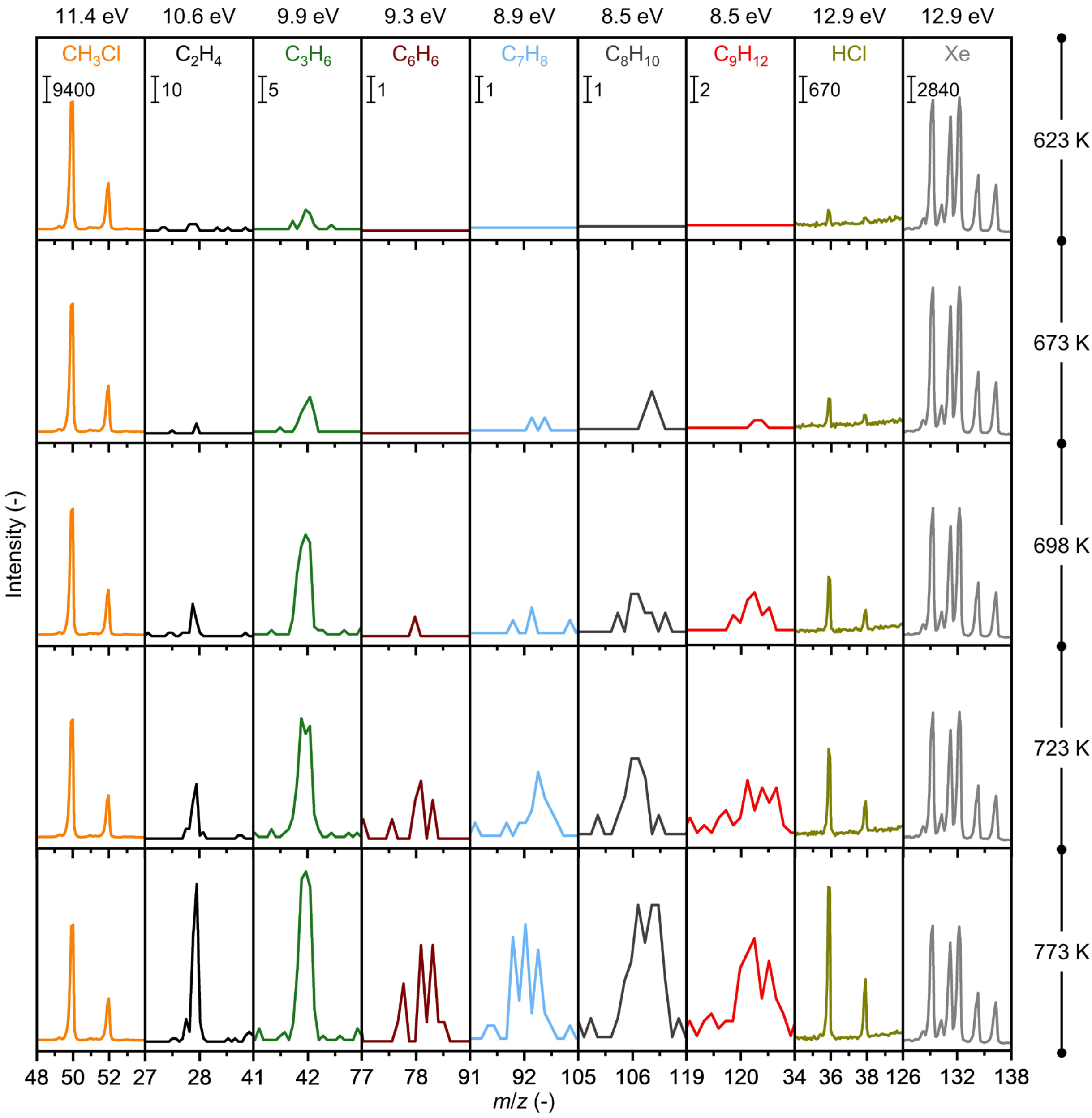

Supplementary Figure 10. Mass spectra of reactants, products, and inert standard at the reactor outlet in MCTH over H-ZSM-5 at different temperatures detected by operando PEPICO spectroscopy. The photon energies at which the signals of each chemical species were recorded are given above the plots. The scales of the *y* axes indicated in the top row apply to all spectra in the same column. Conditions: CH_3Cl:Xe:Ar = 1:0.1:20.9, F_T = 22 cm^3 STP min^{-1}, W_{cat} = 0.01 g, P = 0.05 bar.

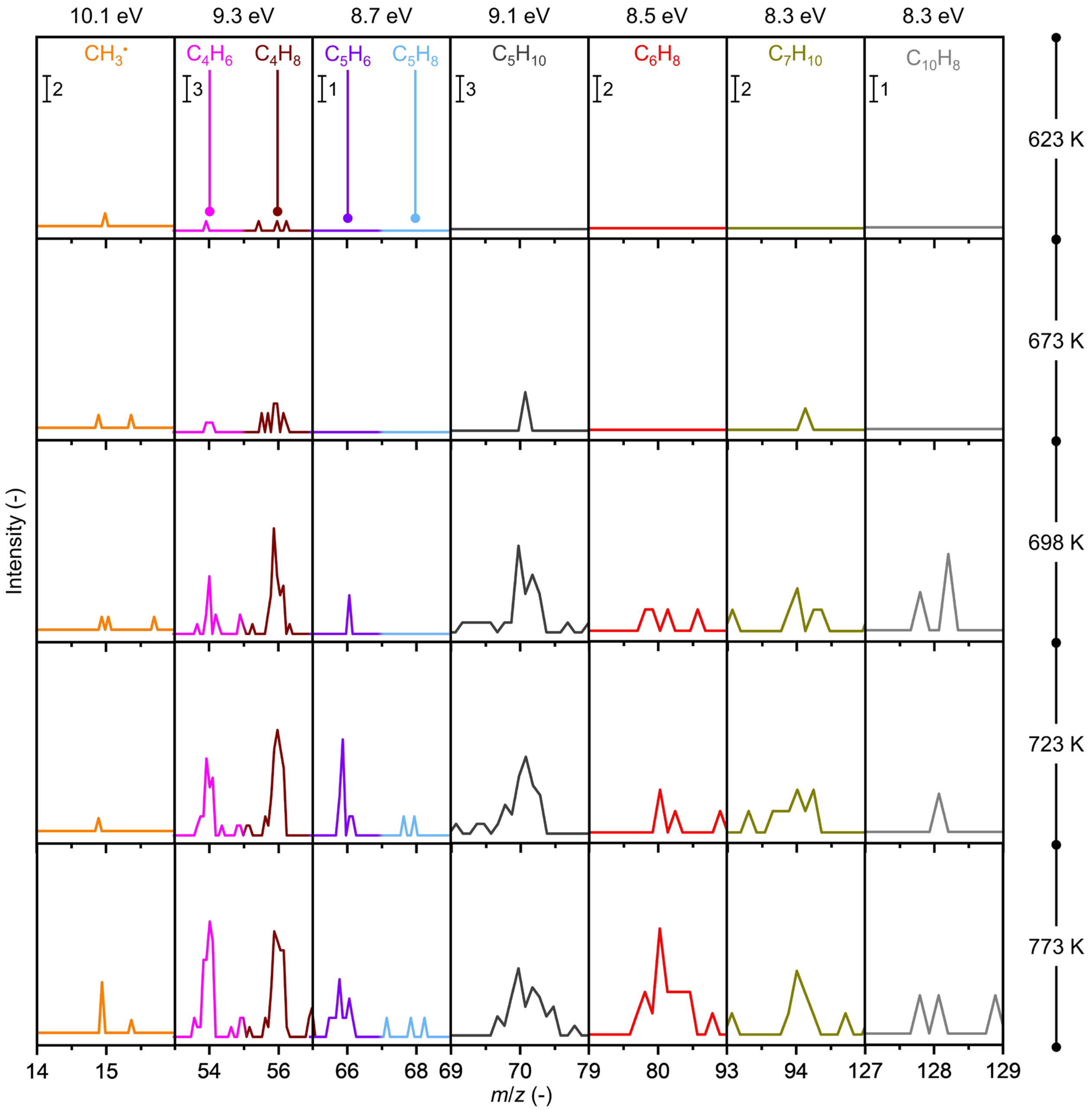

Supplementary Figure 11. Mass spectra of intermediate species at the reactor outlet in MCTH over H-ZSM-5 at different temperatures detected by operando PEPICO spectroscopy. The photon energies at which the signals of each chemical species were recorded are given above the plots. The scales of the *y* axes indicated in the top row apply to all spectra in the same column. Conditions: CH_3Cl:Xe:Ar = 1:0.1:20.9, F_T = 22 cm^3 STP min^{-1}, W_{cat} = 0.01 g, P = 0.05 bar.

Supplementary Figures – Blank operando PEPICO experiments

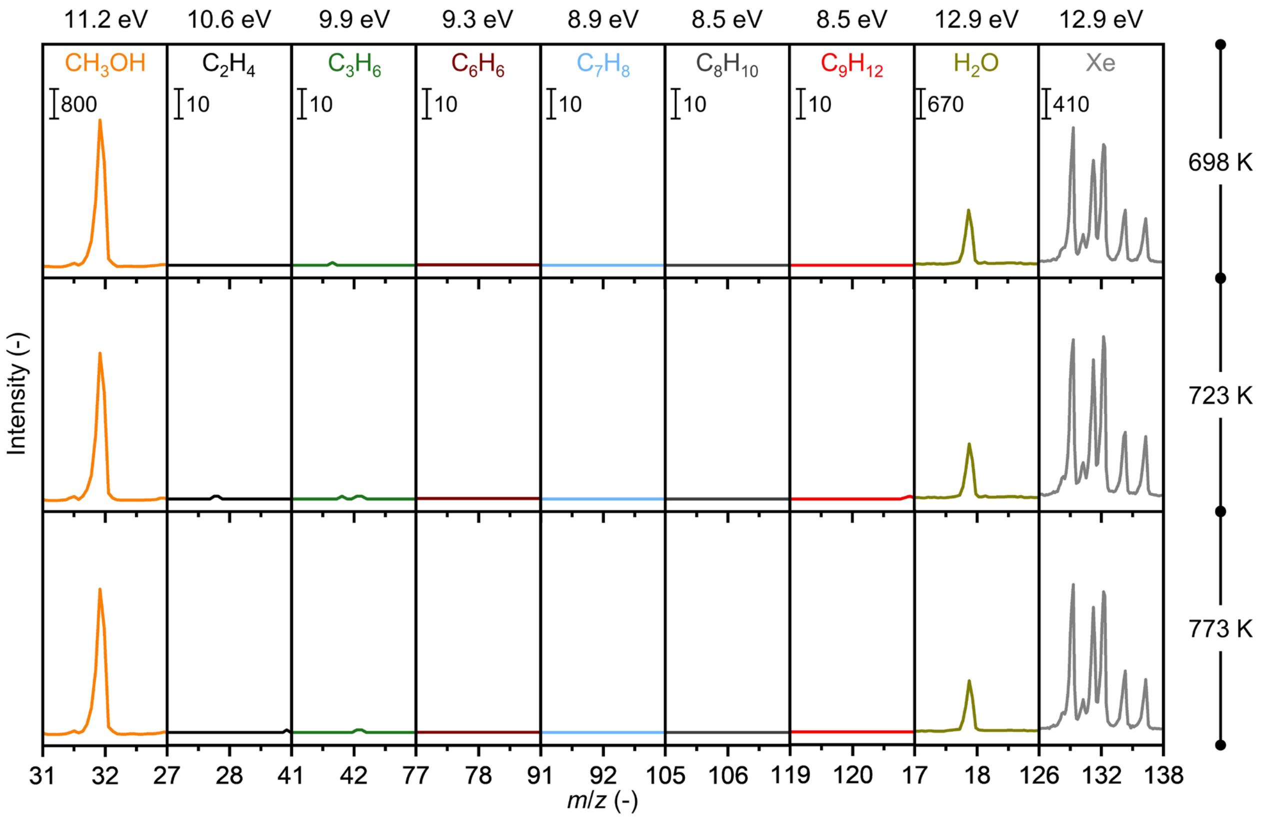

Supplementary Figure 12. Mass spectra of reactants, products, and inert standard at the reactor outlet in MTH performed using an empty reactor at different temperatures detected by operando PEPICO spectroscopy. The photon energies at which the signals of each chemical species were recorded are given above the plots. The scales of the y axes indicated in the top row apply to all spectra in the same column. Conditions: CH_3OH:Xe:Ar = 1:0.1:20.9, F_T = 22 cm^3 STP min^{-1}, P = 0.03 bar.

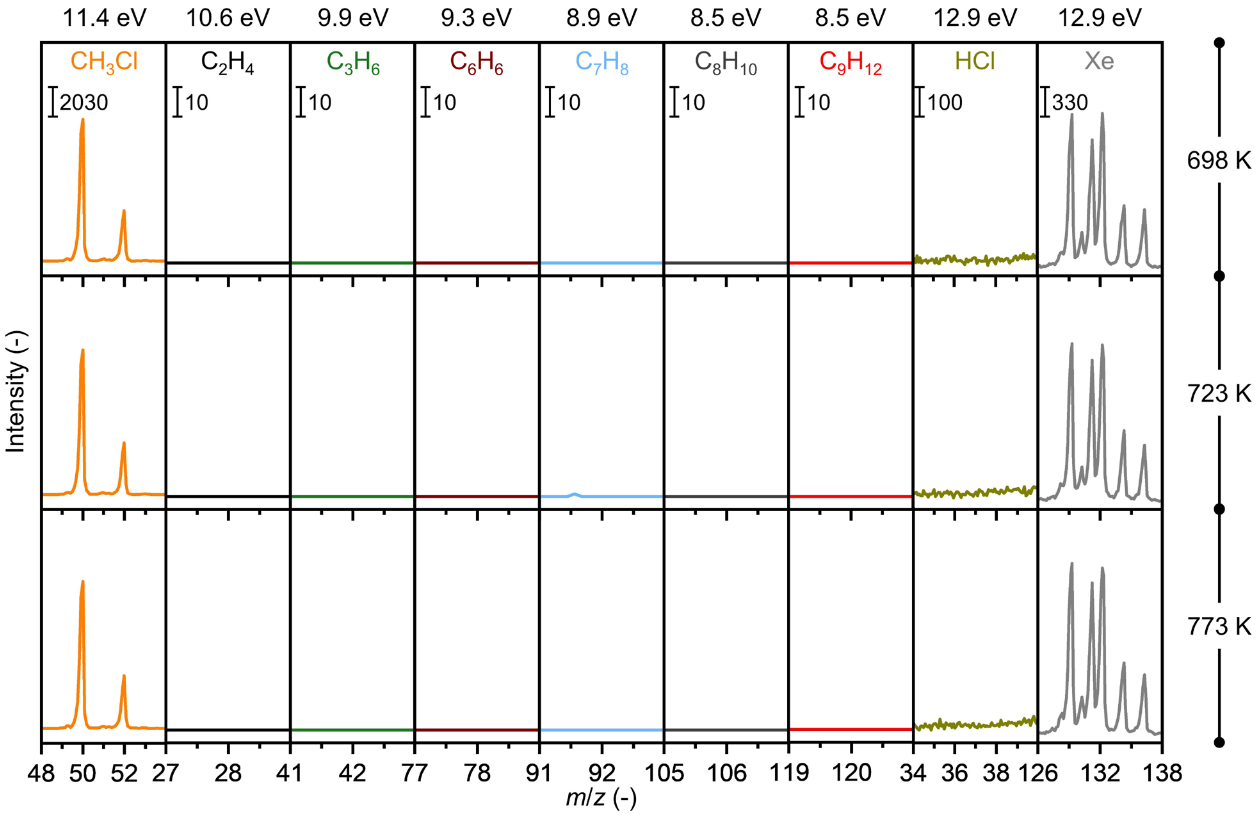

Supplementary Figure 13. Mass spectra of reactants, products, and inert standard at the reactor outlet in MCTH performed using an empty reactor at different temperatures detected by operando PEPICO spectroscopy. The photon energies at which the signals of each chemical species were recorded are given above the plots. The scales of the *y* axes are indicated on the top panels. Conditions: CH_3Cl:Xe:Ar = 1:0.1:20.9, F_T = 22 cm^3 STP min^{-1}, P = 0.03 bar.

Supplementary Figures – Product evolution by operando PEPICO

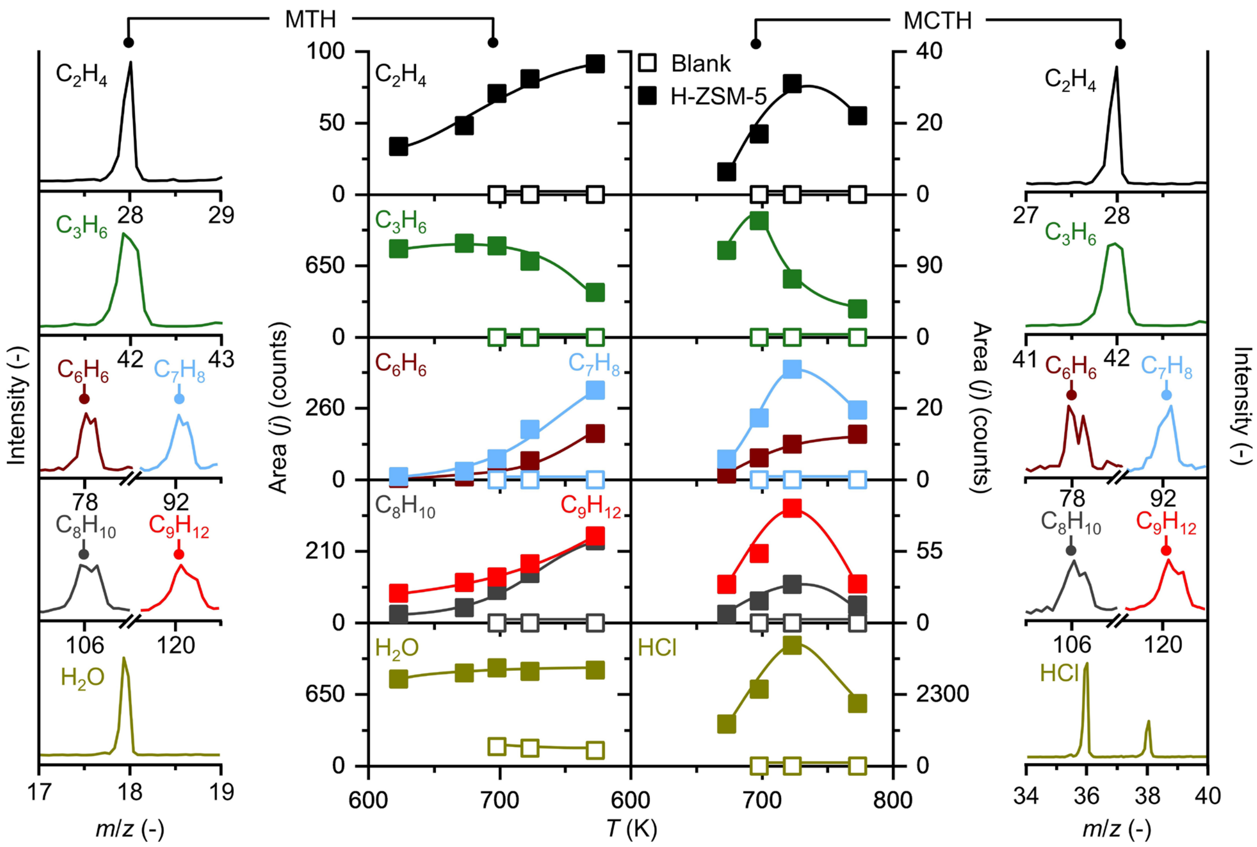

Supplementary Figure 14. Peak areas of the main products as a function of temperature in MTH and MCTH over H-ZSM-5 determined by operando PEPICO. The blank reactor experiments are shown as reference (open symbols). Representative mass spectra of the products detected in MTH and MCTH over the zeolite at 698 K. The photon energies at which the signals of each chemical species were recorded in all PEPICO experiments are shown in **Supplementary Figs. 4** and **6**. Conditions: CH_3X:Xe:Ar = 1:0.1:20.9, F_T = 22 cm^3 STP min^{-1}, W_{cat} = 0.05 g, P = 0.5 bar.

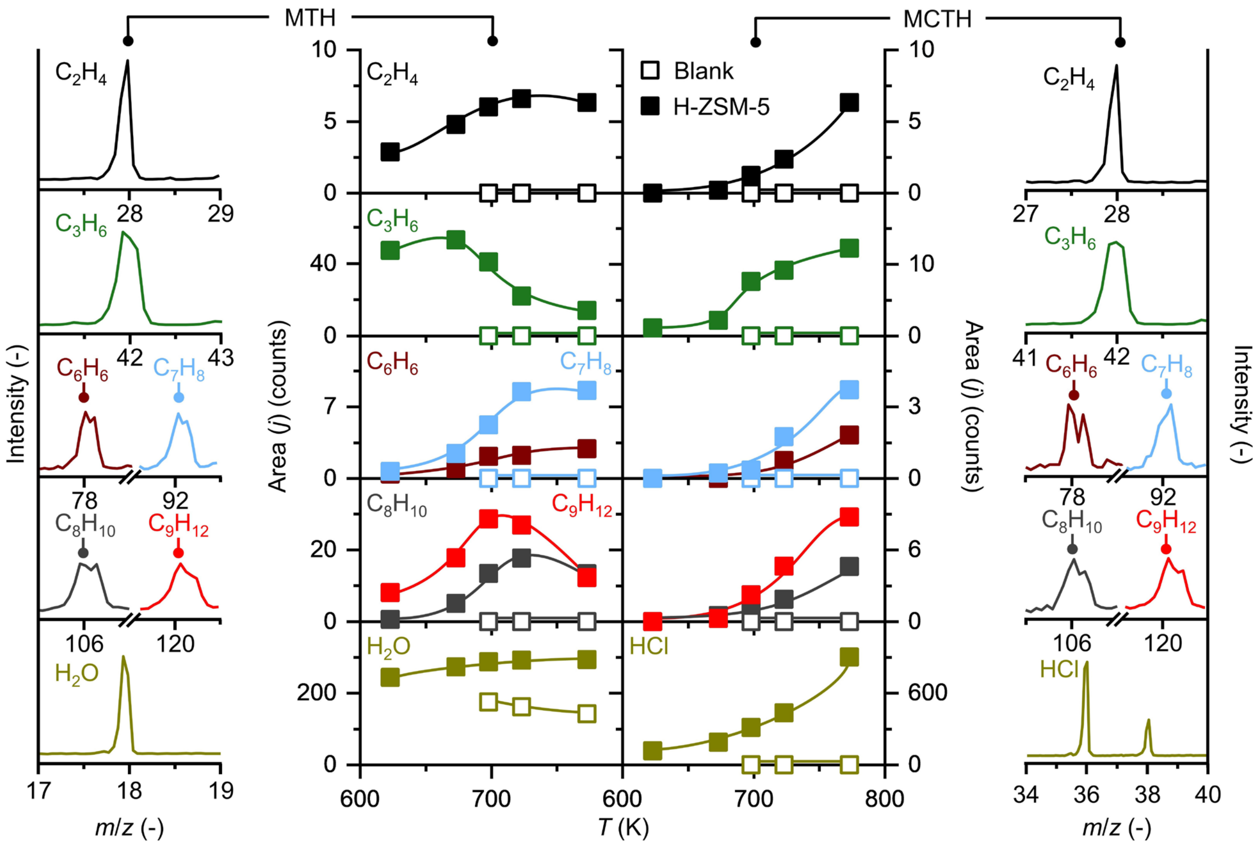

Supplementary Figure 15. Peak areas of the main products as a function of temperature in MTH and MCTH over H-ZSM-5 determined by operando PEPICO. The blank reactor experiments are shown as reference (open symbols). Representative mass spectra of the products detected in MTH and MCTH over the zeolite at 698 K. The photon energies at which the signals of each chemical species were recorded in all PEPICO experiments are shown in **Supplementary Figs. 8** and **10**. Conditions: CH_3X:Xe:Ar = 1:0.1:20.9, F_T = 22 cm^3 STP min^{-1}, W_{cat} = 0.01 g, P = 0.05 bar.

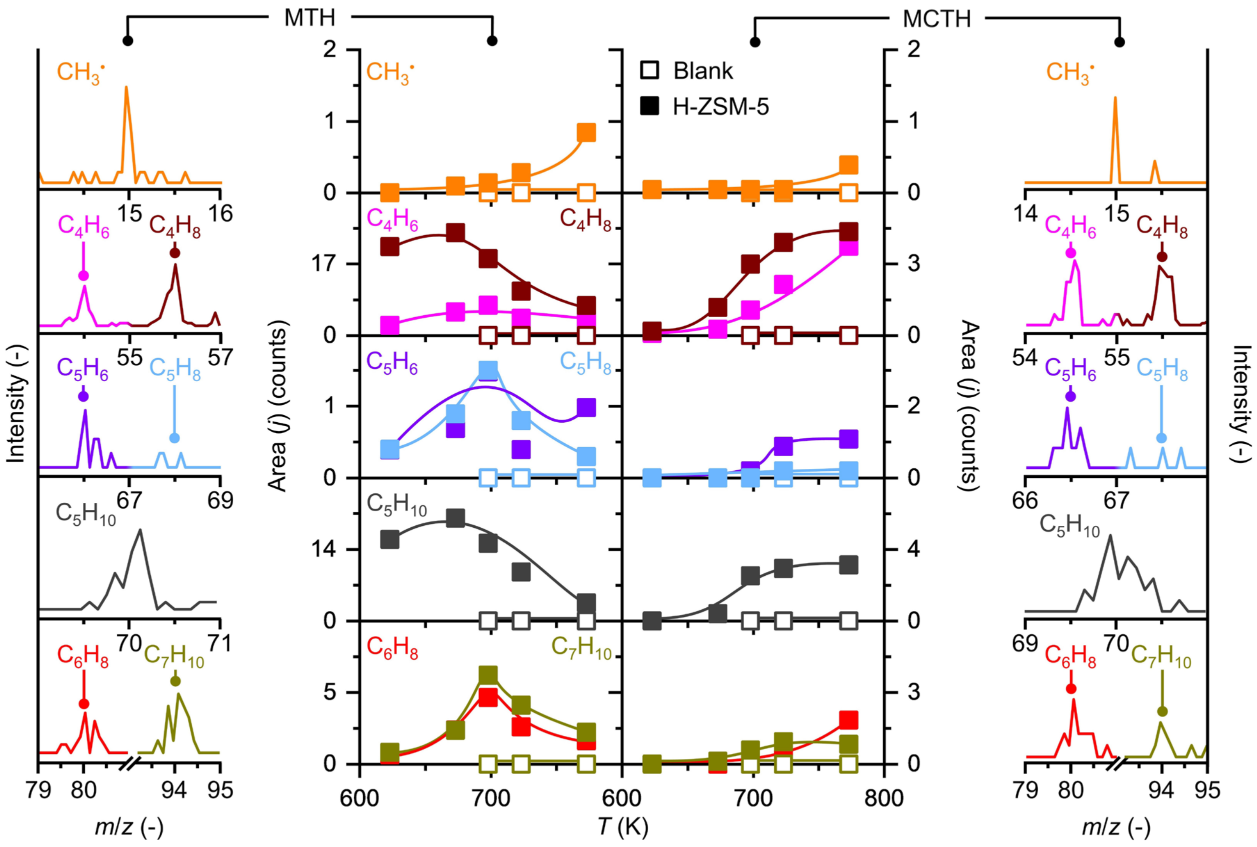

Supplementary Figure 16. Peak areas of the main intermediates as a function of temperature in MTH and MCTH over H-ZSM-5 determined by operando PEPICO. The blank reactor experiments are shown as reference (open symbols). Representative mass spectra of the products detected in MTH and MCTH over the zeolite at 773 K. The photon energies at which the signals of each chemical species were recorded in all PEPICO experiments are shown in **Supplementary Figs. 9** and **11**. Conditions: CH_3X:Xe:Ar = 1:0.1:20.9, F_T = 22 cm^3 STP min^{-1}, W_{cat} = 0.01 g, P = 0.05 bar.

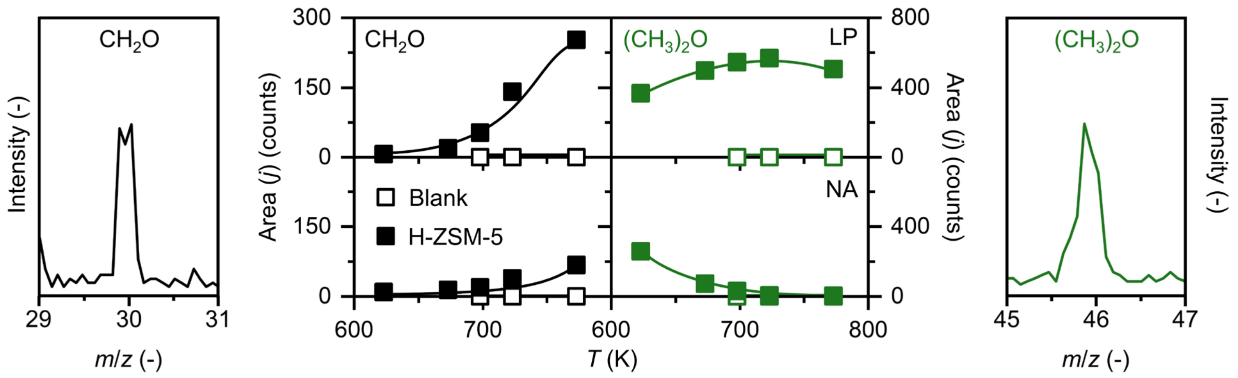

Supplementary Figure 17. Peak areas of formaldehyde and dimethyl ether as a function of temperature in MTH over H-ZSM-5 determined by operando PEPICO. The blank reactor experiments are shown as reference (open symbols). Representative mass spectra of formaldehyde and dimethyl ether detected in MTH over the zeolite at 698 K. The photon energies at which the signals of each chemical species were recorded in all PEPICO experiments are shown in **Supplementary Figs. 5** and **9**. Conditions: CH_3OH:Xe:Ar = 1:0.1:20.9, F_T = 22 cm^3 STP min^{-1}, W_{cat} = 0.01 g, P = 0.05 bar (low pressure, LP) or W_{cat} = 0.05 g, P = 0.5 bar (near ambient, NA).

Supplementary Figures – Isomer analysis by operando PEPICO

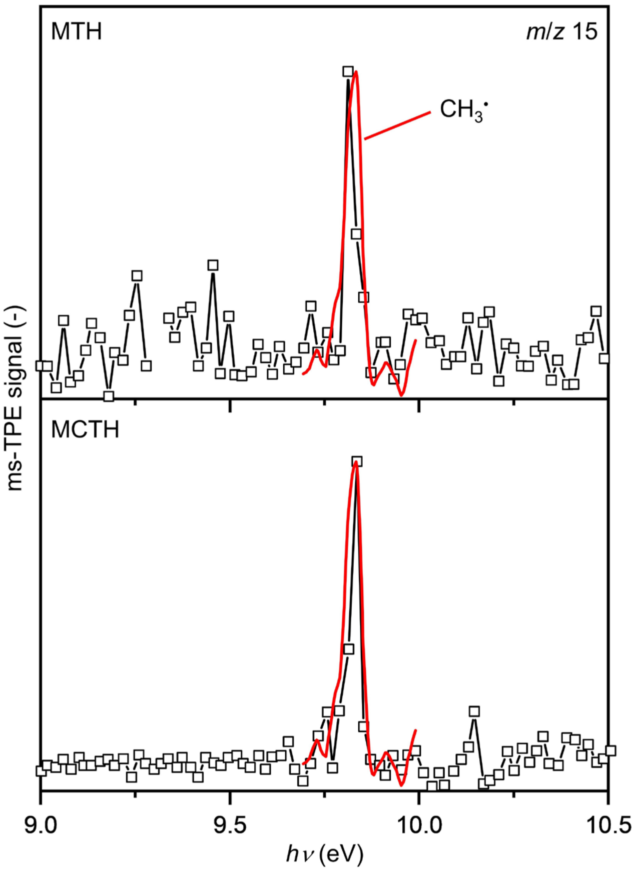

Supplementary Figure 18. Photoion mass-selected threshold photoelectron (ms-TPE; symbols) spectra of *m*/*z* 15 photoions detected in MTH and MCTH over H-ZSM-5 as determined by operando PEPICO. The colored lines denote the reference ms-TPE spectrum. Conditions: CH_3X:Xe:Ar = 1:0.1:20.9, F_T = 22 cm^3 STP min^{-1}, W_{cat} = 0.05 g, P = 0.5 bar.

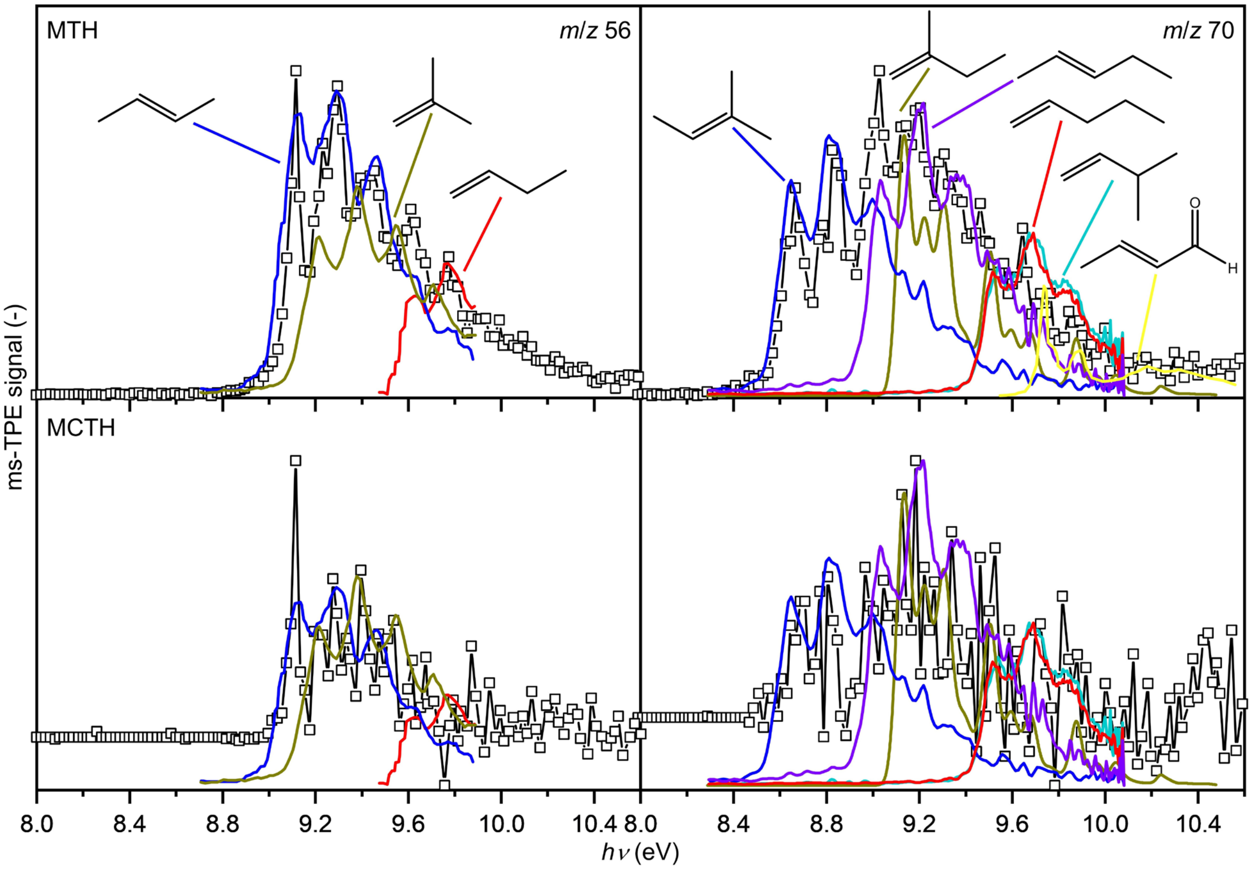

Supplementary Figure 19. Photoion mass-selected threshold photoelectron (ms-TPE; symbols) spectra of *m*/*z* 56 and *m*/*z* 70 photoions detected in MTH and MCTH over H-ZSM-5 as determined by operando PEPICO. The colored lines denote reference ms-TPE spectra of different isomers of *m*/*z* 56 and *m*/*z* 70, whose structures are indicated as insets. Conditions: CH_3X:Xe:Ar = 1:0.1:20.9, F_T = 22 cm^3 STP min^{-1}, W_{cat} = 0.05 g, P = 0.5 bar.

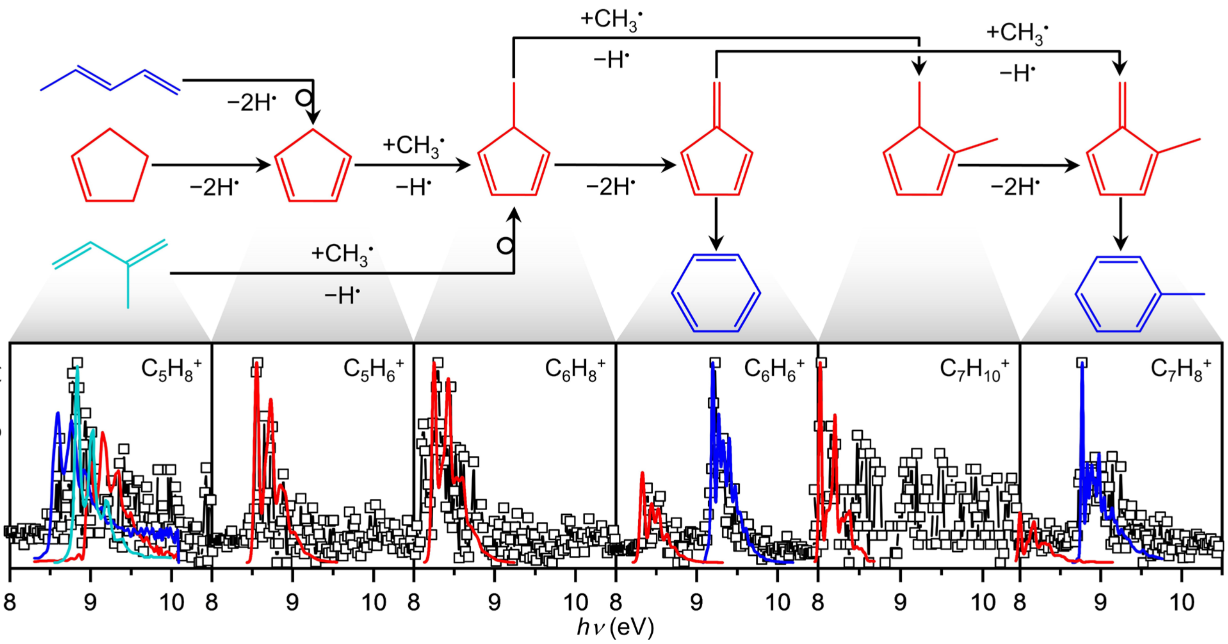

Supplementary Figure 20. Reaction pathway for the chain propagation reaction of C_5H_8 to generate benzene and toluene in MCTH. The ms-TPE (open squares) and reference spectra (solid lines) of the identified products are shown. Conditions: CH_3Cl:Xe:Ar = 1:0.1:20.9, F_T = 22 cm^3 STP min^{-1}, W_{cat} = 0.05 g, P = 0.5 bar.

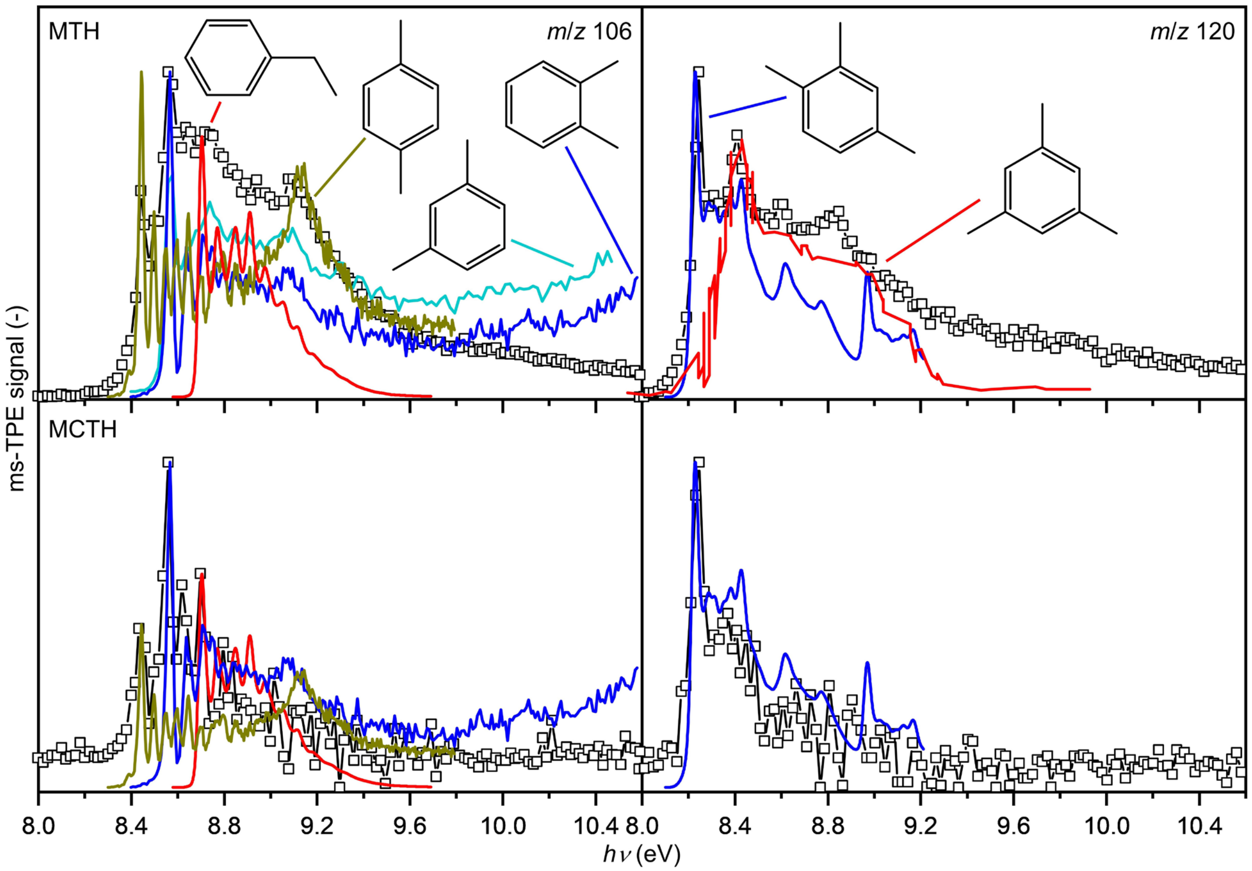

Supplementary Figure 21. Photoion mass-selected threshold photoelectron (ms-TPE; symbols) spectra of *m*/*z* 106 and *m*/*z* 120 photoions detected in MTH and MCTH over H-ZSM-5 as determined by operando PEPICO. The colored lines denote reference ms-TPE spectra of different isomers of *m*/*z* 106 and *m*/*z* 120, whose structures are indicated as insets. Conditions: CH_3X:Xe:Ar = 1:0.1:20.9, F_T = 22 cm^3 STP min^{-1}, W_{cat} = 0.05 g, P = 0.5 bar.

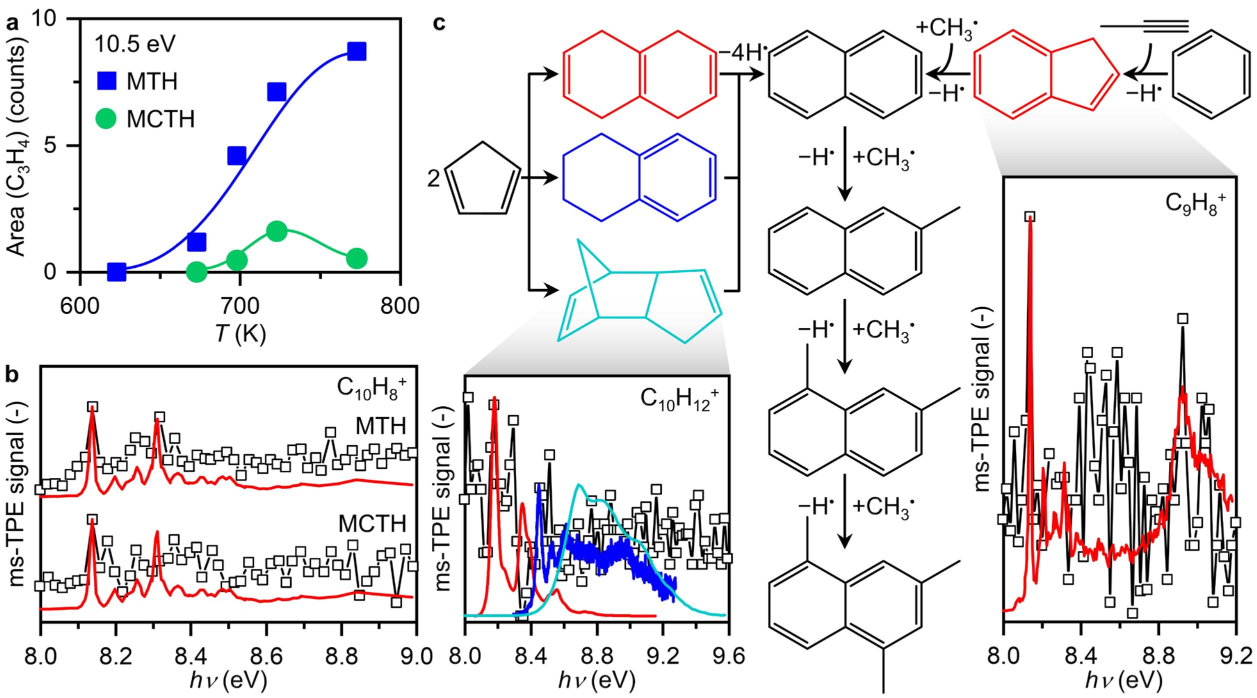

Supplementary Figure 22. a, Extracted peak area of propyne as a function of temperature and **b,** ms-TPE spectra of *m*/*z* 128 photoion detected at the reactor outlet in MTH and MCHT over H-ZSM-5. **c,** Reaction pathway for the formation of naphthalene and methylated naphthalenes in MTH. The insets show the ms-TPE (open squares) and reference spectra (solid lines) of the identified intermediates. Conditions: **a,** T = 623-773 K; **b,c,** T = 773 K; **a-c,** CH_3X:Xe:Ar = 1:0.1:20.9, F_T = 22 cm^3 STP min^{-1}, W_{cat} = 0.05 g, P = 0.5 bar.

Supplementary Figures – Desorption of chemical species under inert atmosphere

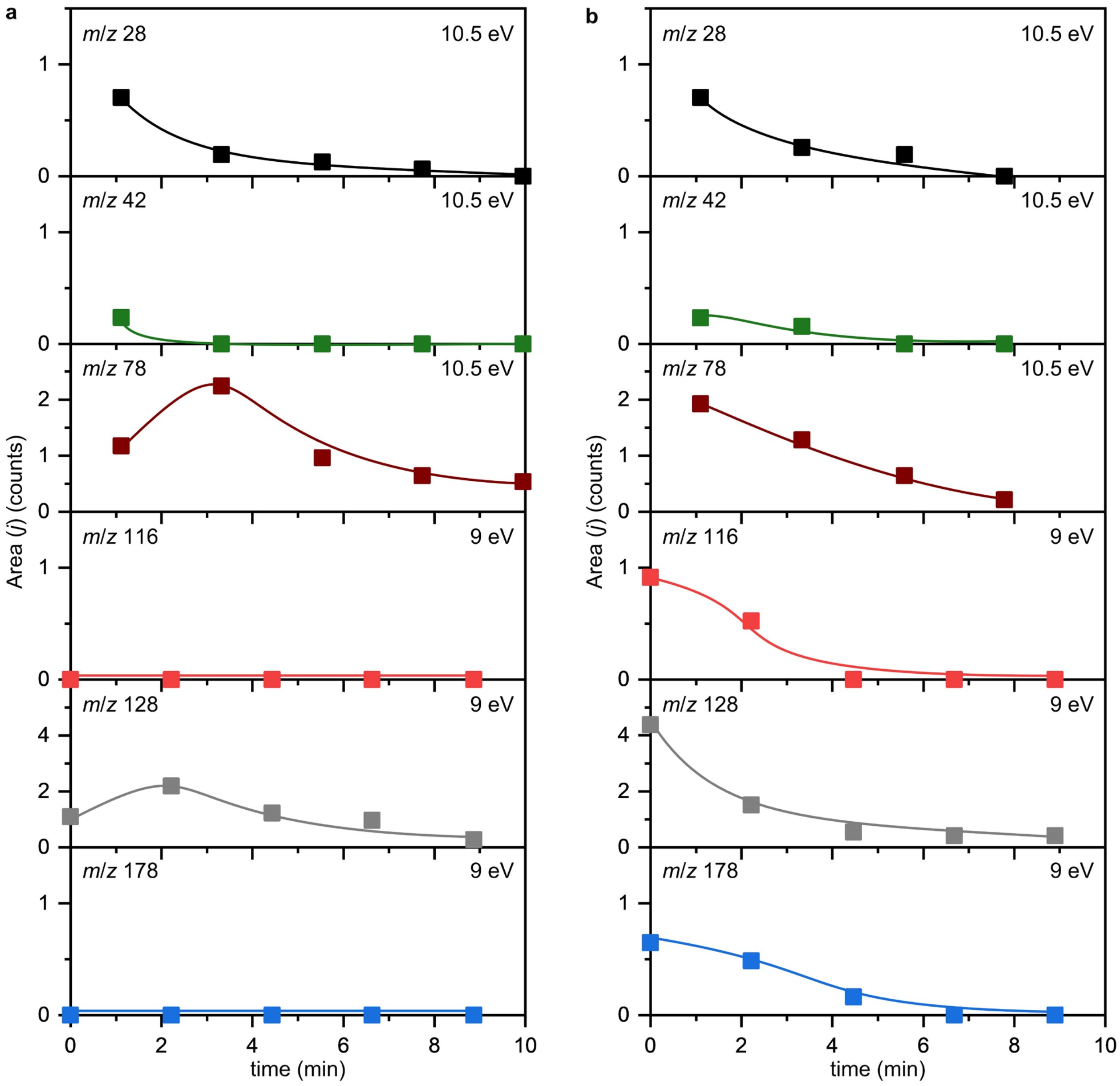

Supplementary Figure 23. Extracted peak area of product *j* as a function of time, which are desorbed from the H-ZSM-5 catalyst after switching off the feed of **a,** CH_3OH or **b,** CH_3Cl (time zero), as determined by operando PEPICO. Conditions: CH_3X:Xe:Ar = 0:0.1:21.9, F_T = 22 cm^3 STP min^{-1}, W_{cat} = 0.05 g, P = 0.5 bar.

Supplementary Figures – Density of coke deposits by cw EPR

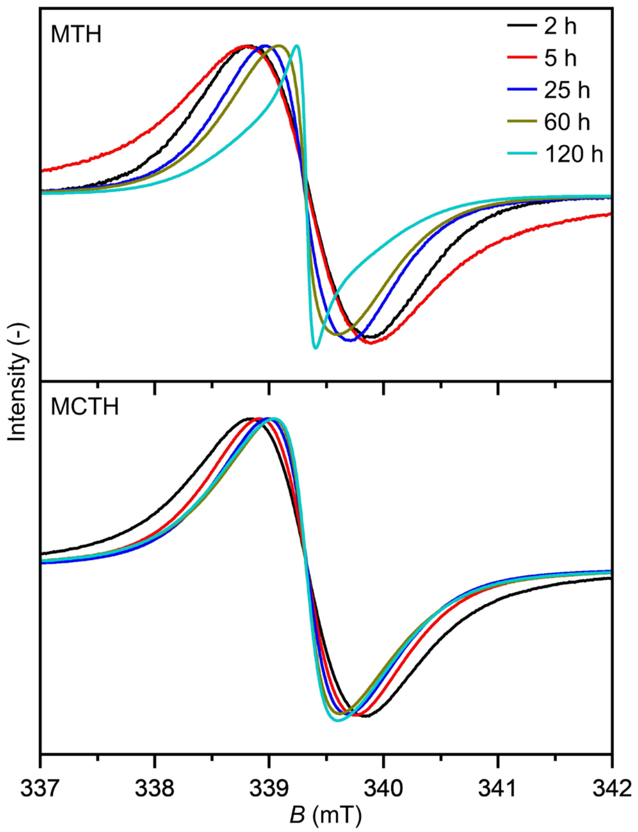

Supplementary Figure 24. Normalized cw EPR spectra of H-ZSM-5 after different time-on-stream in MTH and MCTH. Conditions: CH_3X:He = 1:1, F_T = 20 cm^3 STP min^{-1}, W_{cat} = 0.6 g, T = 673 K, P = 1 bar.

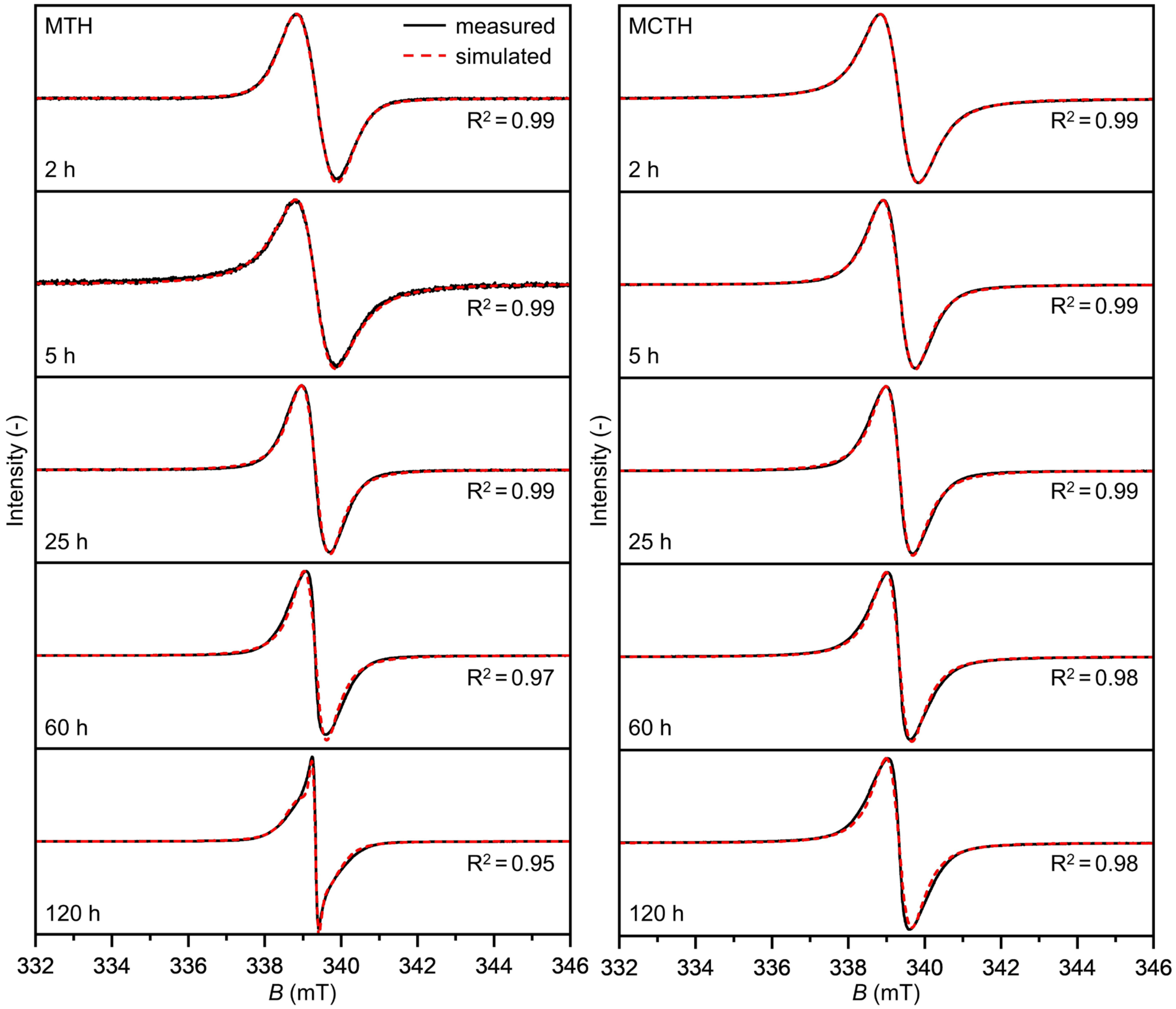

Supplementary Figure 25. Measured and simulated cw EPR spectra of H-ZSM-5 after different time-on-stream in MTH and MCTH. The legend applies to both plots. Conditions: CH_3X:He = 1:1, F_T = 20 cm^3 STP min^{-1}, W_{cat} = 0.6 g, T = 673 K, P = 1 bar.

Supplementary Figures – Coke fractal dimension by pulsed EPR

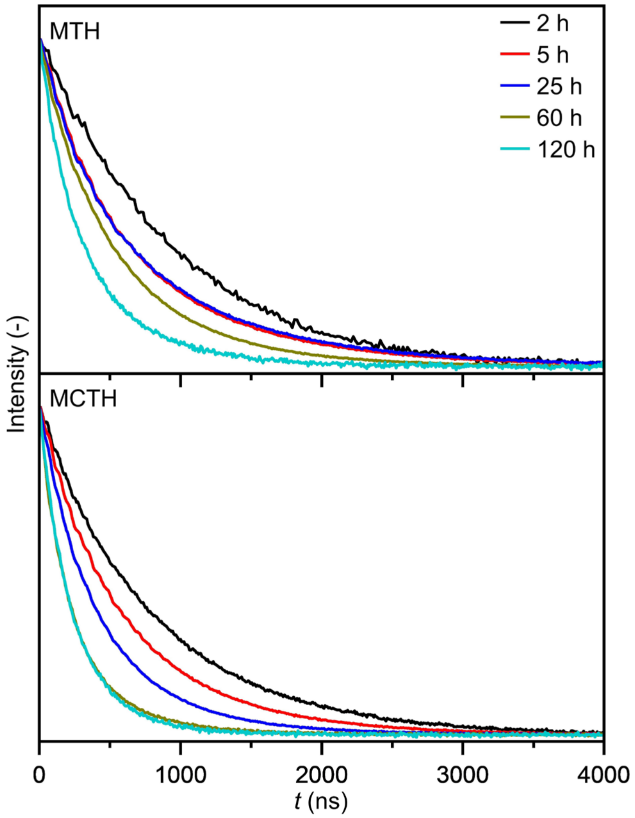

Supplementary Figure 26. Normalized two-pulse electron spin echo decays of H-ZSM-5 after different time-on-stream in MTH and MCTH. Conditions: CH_3X:He = 1:1, F_T = 20 cm^3 STP min^{-1}, W_{cat} = 0.6 g, T = 673 K, P = 1 bar.

Supplementary Figures – Representative molecular structure of carbonaceous deposits derived by 2D HYSCORE

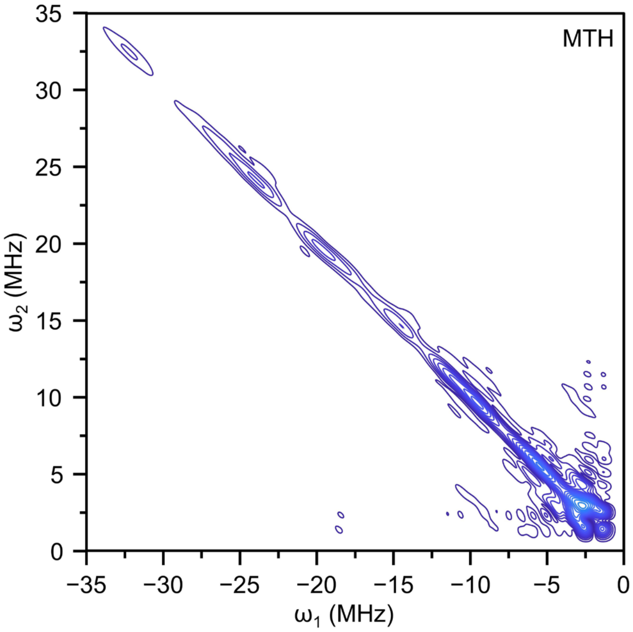

Supplementary Figure 27. Strong interaction quadrant of the 2D HYSCORE spectra of H-ZSM-5 after 2 h on stream in MTH and MCTH. Conditions: CH_3X:He = 1:1, F_T = 20 cm^3 STP min^{-1}, W_{cat} = 0.6 g, T = 673 K, P = 1 bar, *tos* =2 h. The signals on the anti-diagonal after use in MTH are artifacts from non-ideal phase cycling.

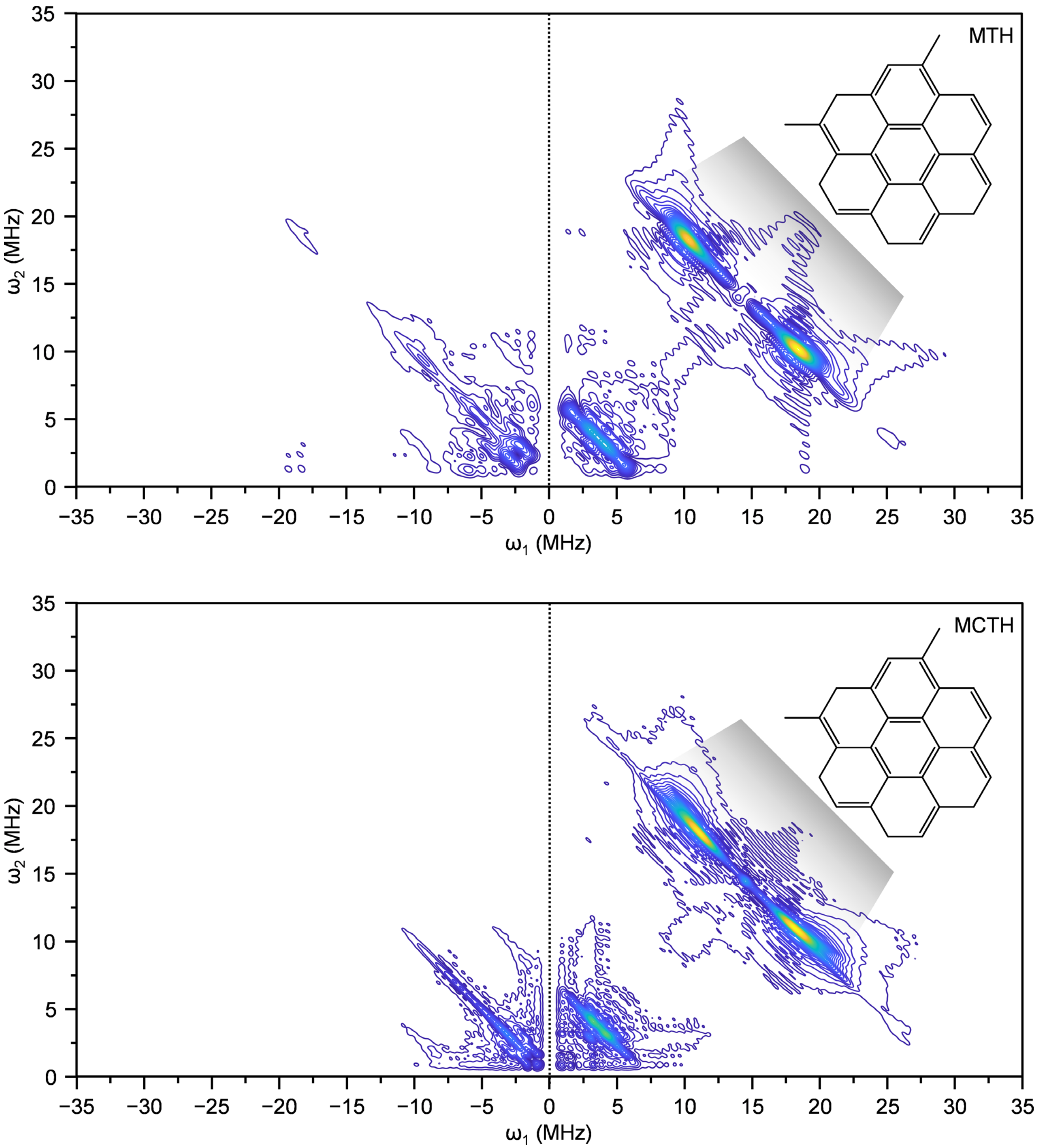

Supplementary Figure 28. Strong (left) and weak (right) interaction quadrants of the 2D HYSCORE spectra of H-ZSM-5 after 60 h on stream in MTH and MCTH. The insets depict a representative molecular structure of coke under these conditions, whose assignment is detailed in Supplementary Note 2. Conditions: CH_3X:He = 1:1, F_T = 20 cm^3 STP min^{-1}, W_{cat} = 0.6 g, T = 673 K, P = 1 bar, tos = 60 h.

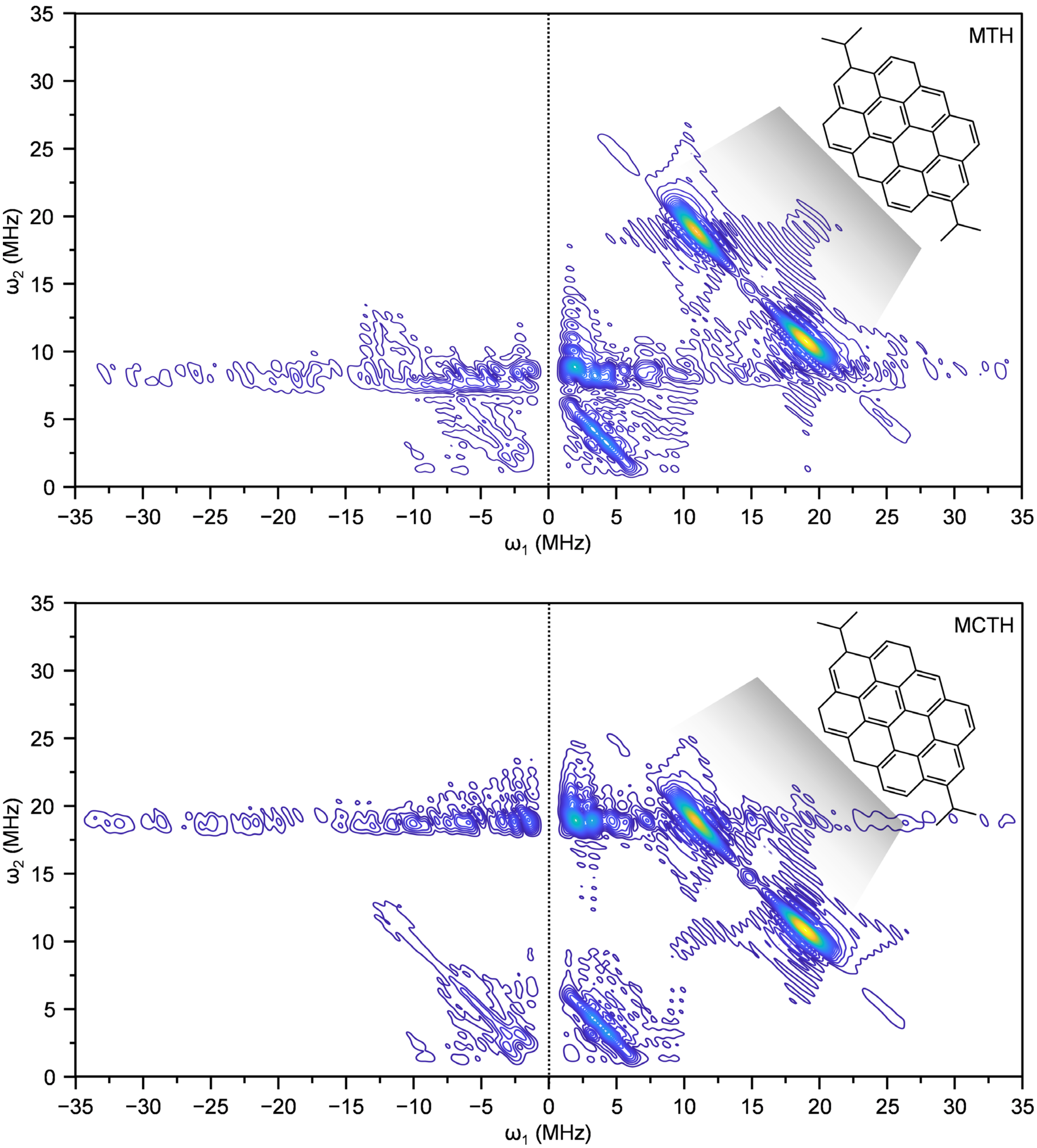

Supplementary Figure 29. Strong (left) and weak (right) interaction quadrants of the 2D HYSCORE spectra of H-ZSM-5 after 120 h on stream in MTH and MCTH. The insets depict a representative molecular structure of coke under these conditions, whose assignment is detailed in Supplementary Note 2. Conditions: CH_3X:He = 1:1, F_T = 20 cm^3 STP min^{-1}, W_{cat} = 0.6 g, T = 673 K, P = 1 bar, *tos* =120 h. The signals on a horizontal frequency of 8 MHz after the MTH reaction and 18 MHz after the MCTH reaction stem from electronic artifacts.

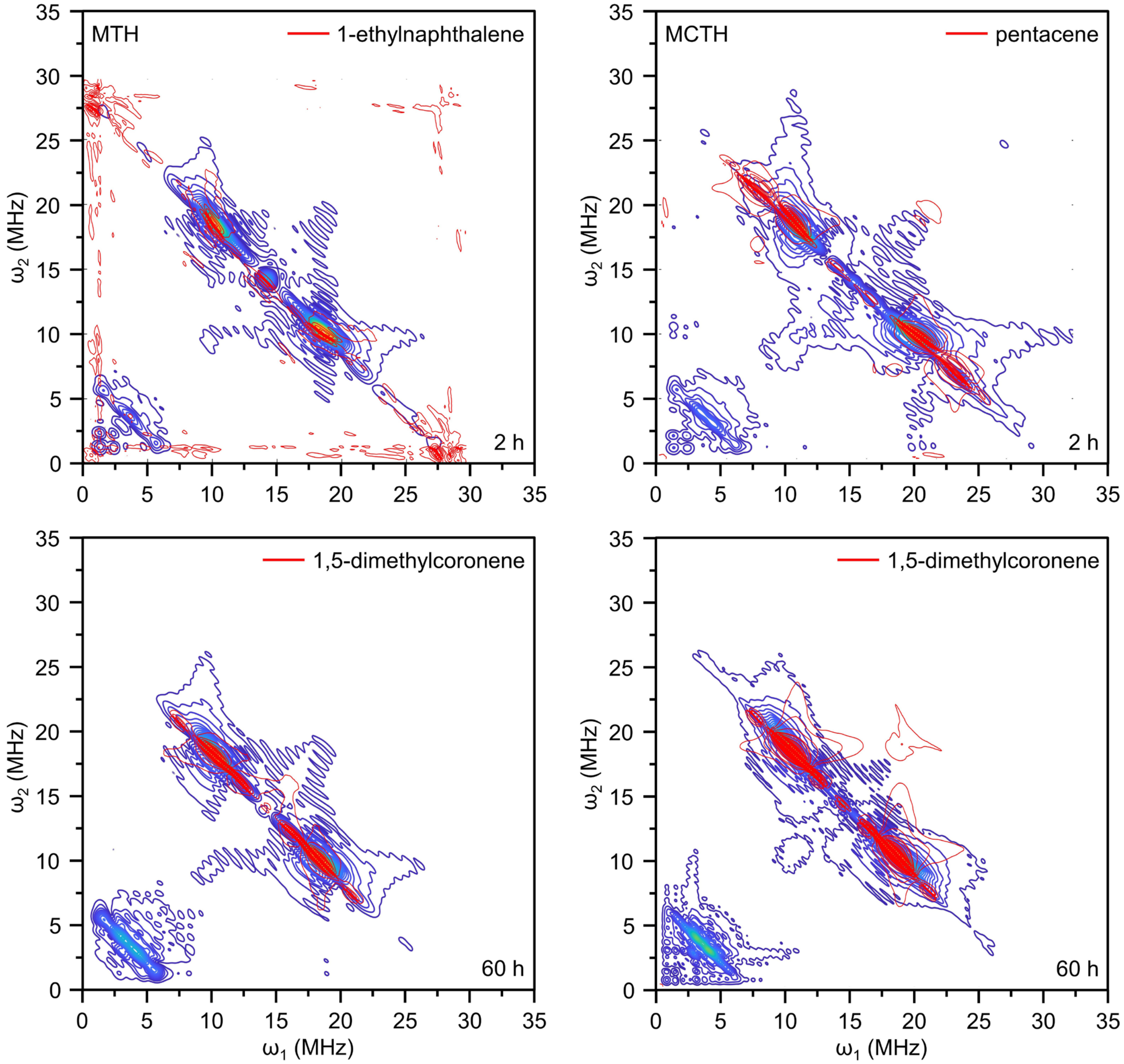

Supplementary Figure 30. Weak interaction quadrant of the 2D HYSCORE spectra of H-ZSM-5 after 2 h and 60 h on stream in MTH and MCTH. The red lines denote the DFT simulated hyperfine couplings of different molecular structures as detailed in Supplementary Note 2. Conditions: CH_3X:He = 1:1, F_T = 20 cm^3 STP min^{-1}, W_{cat} = 0.6 g, T = 673 K, P = 1 bar, *tos* =2, 60 h.

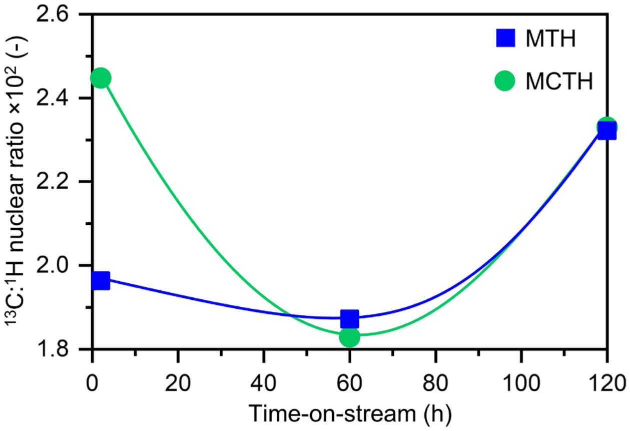

Supplementary Figure 31. ^{13}C:^{1}H nuclear ratio as a function of time-on-stream as determined by 2D HYSCORE measurements. Conditions: CH_3X:He = 1:1, F_T = 20 cm^3 STP min^{-1}, W_{cat} = 0.6 g, T = 673 K, P = 1 bar.

Supplementary Note 2

Density of coke deposits by cw EPR

The normalized cw EPR spectra of H-ZSM-5 within the first 25 h in MTH and MCTH consisted of a single symmetric line with the same isotropic *g* factor ($g = 2.003$). The contribution of a second component was observed in the signals after 60 h and especially 120 h on stream, while maintaining the same *g* factor. Consequently, the lineshape changed considerably as a function of time-on-stream (*tos*), as shown in **Supplementary Fig. 24**. The changes in linewidth depended on the reaction; in MCTH it decreased steadily, while in MTH it first increased from 2 h to 5 h on stream and subsequently diminished until 120 h in the reaction. To gain insight into the density of coke deposits, these spectra were least-squares fitted to extract their Gaussian and Lorentzian contributions. While dense deposits result in local magnetic interactions producing a Lorentzian-type EPR signal, virtually isolated paramagnetic species generate an EPR spectrum with Gaussian lineshape. Accordingly, the cw EPR spectra were linearly baseline-corrected and subsequently simulated *via* EasySpin routines. A one component function, $f(B)$, was used in the simulations with isotropic *g* tensor and spin quantum number, $S = ½$, according to Eq. 5,

$$f(B) = \omega_G \times \frac{dG(B)}{dB} + \omega_L \times \frac{dL(B)}{dB} \qquad \text{Eq. 5}$$

where $G(B)$ and $L(B)$ are Gaussian and Lorentzian lineshape functionals, respectively, and ω_G and ω_L are their corresponding weighted fractions. The observed spectral characteristics are expected for a broad distribution of aromatic cation radicals, forming the EPR-active part of coke deposited inside the zeolite micropores. The spectra of H-ZSM-5 after MCTH could be well reproduced with these assumptions, except the one after 120 h on stream that showed a slight asymmetry. In the case of H-ZSM-5 after 120 h in MTH, significant discrepancies between the simulated and the measured spectra were observed. These signals could be well described by using a two-component function, which is a weighted superposition independent Gaussian and Lorentzian contributions. The comparison between the experimental and simulated spectra is plotted in **Supplementary Fig. 25**. Accordingly, the relative Gaussian and Lorentzian contribution to the lineshape of the EPR signals as a function of time-on-stream could be extracted, as shown in **Fig. 5d** of the main manuscript. While after 2 h in MCTH the linewidth of the EPR spectra evidenced a Gaussian contribution, longer reaction times resulted almost exclusively in a Lorentzian-type signal. On the other hand, the H-ZSM-5 after 2 h in MTH yields an EPR signal with a dominant Gaussian contribution, while the Lorentzian contribution became dominant after 60 h on stream. Furthermore, it is noteworthy that the linewidth of the recorded spectra decreased as a function of time-on-stream in both MTH and MCTH, as shown in **Supplementary Fig. 24**. Previous studies in zeolite catalysis suggested that this narrowing is related to the growth of polyaromatic molecules,[46,47] leading to an increased electron spin density delocalization over the aromatic systems. For this hypothesis to hold, it is implicitly assumed that the spectral lineshape is mainly determined by hyperfine splitting. The isotropic Fermi contact hyperfine coupling, a_{iso}, with the ^{1}H nuclei interacting with the electron spin is proportional to the electron spin density

at these nuclei. In a cw EPR spectrum, this is manifested as a splitting of the main signal into a series of lines, whose number is expressed by Eq. 6,

$$\prod_{i}^{m}(n_i+1) \quad \text{Eq. 6}$$

for m groups of n_i equivalent protons that separated by a_{iso} and with intensity ratios corresponding to the binomial coefficients. For a large number of protons, this leads to a vast number of signals, which cannot be resolved by a cw EPR spectrum, resulting in a single line with Gaussian lineshape. An overall signal narrowing is expected with an increasing number of protons, which proportionally lessens the magnitude of hyperfine couplings due to spin density delocalization.

The fitting of our experimental spectra showed that virtually all samples exhibited a predominantly Lorentzian lineshape. Moreover, the comparison between the experimental and the simulated spectra, which were calculated by using a_{iso} hyperfine constants obtained from preliminary Hückel calculations, showed that the experimental linewidth is generally lower than expected for any typical aromatic and polyaromatic molecule. This suggests that the observed lineshape is dominated by exchange interactions between nearby radicals and that the narrowing is due to an increase in local concentration. This phenomenon is called exchange narrowing, and it is observed when such interplay is so intense that the exchange-correlation frequency is higher than the individual inhomogeneous linewidth. Accordingly, a narrowing of the lineshape can be interpreted as an increment in the density of paramagnetic coke species. Furthermore, the amount of EPR-active coke was higher during MCTH, generating signals with Lorentzian lineshape indicating that exchange interactions are dominant. These phenomena are lessened in MTH as it is reflected in the evolution of the lineshape with time-on-stream. After 2 h in MTH, the shape of the EPR spectra is determined by hyperfine broadening, resulting in the observed Gaussian line. As the local density of paramagnetic coke increases, a Lorentzian lineshape is observed after 5 h in MTH, which is due to dipolar broadening at low local spin concentrations.[49] Notably, after 25 h in MTH, weak exchange coupling becomes significant, resulting in an increment of the Gaussian contribution increased (**Fig. 5d** of the main manuscript), due to exchange broadening. For longer times-on-stream, exchange interactions prevail as the local density increases, yielding a Lorentzian-type line with decreasing width. Accordingly, it can be concluded that the phenomena observed during MTH correspond to the initial stages of coking, while in the case of MCTH this process is significantly faster, in agreement with the kinetic analysis and the spin-echo decay measurements presented in the main manuscript.

As anticipated, an additional feature was observed in the EPR spectra of the zeolite treated for 120 h in MTH, which might be also present after 120 h in MCTH. These two signals could be partially decoupled with echo-detected field-sweep EPR at the maximum echo obtained at different microwave powers. Thus, the secondary signal could be assigned to thermally-accessible triplet states. These can be observed for large aromatic systems with at least two sufficiently distant alkylic groups, where the two unpaired electrons are mainly localized. Previous density functional theory (DFT) calculations estimated that species, which stabilize such states, contain at least 10 aromatic rings,[50] as shown in **Supplementary Fig. 29**.

Representative molecular structure of coke deposits by 2D HYSCORE

Due to a large amount of coupled protons, the hyperfine structure in cw EPR cannot be resolved. Therefore, we performed 2D HYSCORE experiments to distinguish the hyperfine couplings shown in **Fig. 5f,g** of the main manuscript and **Supplementary Figs. 27-29**. These measurements evidenced several signals due to hyperfine interactions with different magnetic nuclei. A typical 2D HYSCORE spectrum is divided into two quadrants: the weak coupling quadrant, designated as (+,+), in which hyperfine couplings that have hyperfine coupling constant, A, below two times the Larmor frequency of a nucleius, ν_I, can be distinguished; and the strong coupling quadrant, designated as (−,+), which shows couplings with $A > 2\times \nu_I$. For highly isotropic interactions, a coupling with a group of equivalent nuclei gives rise to two correlating peaks along the antidiagonal. In the (+,+) quadrant, they are centered at ν_I and separated by a_{iso}, while in the (−,+) quadrant, they are centered at $a_{iso}/2$ and separated by $2\times\nu_I$. Still, anisotropic couplings are present, and the complete hyperfine interaction can be described as a tensor, according to Eq. 7,

$$\mathbf{A} = a_{\text{iso}} \times \mathbf{I} + \mathbf{T} \qquad \text{Eq. 7}$$

where **A** is the matrix of hyperfine coupling constants, **I** is a unity matrix, **T** is a 3×3 matrix, quantifying the anisotropic dipolar interaction between electron and nuclear spins. The anisotropic hyperfine couplings broaden the 2D HYSCORE signals and lead to typical curved ridges.

The spectra of all the analyzed samples showed a series of signals in the (+,+) quadrant due to couplings with (*i*) ^{1}H at ca. 14 MHz, (*ii*) ^{29}Si at ca. 3 MHz, as well as (*iii*) ^{13}C and (*iv*) ^{27}Al nuclei at ca. 3.6 MHz. In addition, signals due to interactions with ^{13}C and possibly ^{35}Cl nuclei, in the case of zeolite after MCTH, are shown in the (−,+) quadrant. In particular, the signals associated with ^{1}H nuclei are highly structured and well resolved, which allows deriving information on the composition of the carbonaceous species deposited within the zeolite micropores. While the 2D HYSCORE spectra evidenced complex mixtures of aromatic species in all samples studied, it was possible to extract a representative molecular structure by comparison with spectral simulations based on DFT (**Supplementary Fig. 30**), reported data, and cw EPR signals. While after 60 h and 120 h in MTH and MCTH the 2D spectra looked virtually identical (**Supplementary Figs. 28,29**), strong differences were observed in the first 2 h of reaction (**Fig. 5f,g** of the main manuscript). These conditions are closer to the on-set of coke formation, where distinctions between the two reactions are maximized, while with increasing time-on-stream the distribution of coke species broadens and can no longer be resolved into components. Notably, a higher resolution of low-frequency signals was observed after 2 h in MCTH compared to MTH. This indicates that species with a high symmetry are predominantly formed in MCTH, while a large number of symmetry-inequivalent nuclei led to broader signals in MTH. To assign the observed peaks to representative molecular structures, Hückel calculations were firstly performed on a large number of linearly and non-linearly condensed polyaromatic molecules of different sizes, resulting in good approximations of isotropic Fermi coupling constants. On this basis, DFT calculations were performed for different molecular structures in order to more accurately

estimate the a_{iso} values and the anisotropic dipole-dipole hyperfine couplings. The 2D spectrum obtained after 2 h in MCTH could be described by a distribution of polyacenes, such as tetracene, pentacene, and hexacene. By comparing the modeled structures with the experimental data, the carbonaceous species formed after 2 h in MCTH can be represented by pentacene (**Fig. 5g** of the main manuscript, **Supplementary Fig. 30**, **Supplementary Table 3**). Hyperfine couplings corresponding to shorter linear (naphthalene, anthracene) and non-linear polyacenes (e.g., phenanthrene, coronene), are not able to reproduce the observed 2D spectra, although their presence cannot be excluded. This is an agreement with reported 2D HYSCORE spectra of linear paramagnetic polyacene, such as pentacene.[51] Furthermore, high-frequency signals at 21 MHz and 26 MHz as well as a diagonal peak corresponding to weakly coupled protons, were observed in the 2D spectrum after 2 h in MTH. These contributions were absent after MCTH. In agreement with literature,[52] these couplings could be assigned to paramagnetic alkyl chains. By comparison with the calculated structures (**Supplementary Fig. 30**, **Supplementary Table 3**), the 2D spectra obtained after 2 h in MTH could be represented by 1-ethylnaphthalene (**Fig. 5f** of the main manuscript). Although other compounds are likely present, shorter polyaromatic chains with a higher alkylation degree are formed in the initial stages of MTH, while MCTH tends to form highly symmetric and longer aromatic systems. This interpretation also agrees with the lower peak resolution observed in MTH, which is given by alkylic groups that break the D_{2h} symmetry of linear polyacenes, inducing a large number and a broad distribution of hyperfine coupling constants for different protons. This is further corroborated by estimating the ^{13}C:^{1}H nuclei ratio from the 2D HYSCORE maximum intensities, which was considerably higher after 2 h in MCTH than in MTH (**Supplementary Fig. 31**). Although this estimation does not correspond to the C:H molar ratio obtained from the representative molecular structures, it is consistent with higher alkylation and lower condensation degrees of the carbonaceous species formed during MTH.

The spectral features and ^{13}C:^{1}H nuclei ratios obtained after 60 h and 120 h in MTH and MCTH were very similar, pointing towards a broad and comparable coke composition. By comparison with literature[51] and the simulated structures, the 2D spectra after 60 h on stream could be described by large extended aromatic species with moderate alkylation, such as 1,5-dimethylcoronene (**Supplementary Fig. 28**, **Supplementary Table 3**). The evolution from linear polyacenes to 2D-extended polyaromatic systems is further evidenced by the signals attributed to ^{13}C nuclei at ca. 1 MHz (**Supplementary Fig. 28**). While these hyperfine splitting were unresolved during the initial stages of the reaction, these signals were stronger and resolved after 60 h in MTH and MCTH. Finally, in agreement with reported DFT calculations[50] and with the results from cw and pulse EPR experiments, 1,12-diisopropyltribenzocoronene was assigned as a representative molecular structure of the carbonaceous species formed after 120 h on stream in MTH and MCTH (**Supplementary Fig. 29**). Notably, an electronic artifact was observed in these spectra, which does not originate from any paramagnetic species. However, its presence does not influence the conclusions drawn.

Supplementary methods

Catalyst characterization

The content of Si and Al in the H-ZSM-5 zeolite was confirmed by inductively coupled plasma optical emission spectroscopy (ICP-OES) using a Horiba Ultima 2 instrument equipped with a photomultiplier tube detector. Ar sorption at 77 K was measured in a Micromeritics TriStar II analyzer. Samples (0.15 g) were degassed to 50 mbar at 423 K for 48 h prior to the measurement. The *t*-plot method was used to estimate the micropore volume and the mesopore (external) surface area. The mesopore volume was estimated by subtraction of the micropore volume from the pore volume determined from the total amount of argon adsorbed at $p/p_0 = 0.98$. Powder X-ray diffraction (XRD) was measured using a PANalytical X'Pert PRO-MPD diffractometer with Cu-Kα radiation (λ = 1.54060 Å). The data was recorded in the 10-70° 2θ range with an angular step size of 0.017° and a counting time of 0.51 s per step. Fourier transform infrared spectroscopy of adsorbed pyridine (Pyridine-FTIR) was conducted in a Bruker Vertex 70 spectrometer. Samples were pressed into self-supporting wafers (5 ton m^{-2}, ca. 1 cm^2), degassed under vacuum (10^{-3} mbar) at 423 K for 2 h, and exposed to pyridine vapour at room temperature. Gaseous and weakly adsorbed molecules were removed by evacuation at room temperature for 15 min, and then at 423 K for 30 min. Spectra were acquired in the 4,000−650 cm^{-1} range with a resolution of 4.0 cm^{-1} and by co-addition of 64 scans. The total concentration of Brønsted and Lewis acid sites were calculated from the areas of adsorbed pyridine bands at 1545 and 1454 cm^{-1}, using extinction coefficients of $\varepsilon_{Brønsted}$ = 1.67 cm $mmol^{-1}$ and ε_{Lewis} = 2.94 cm $mmol^{-1}$ reported by Emeis.[53] Prior to the XRD and IR measurements, the samples were calcined in static air at 773 K (5 K min^{-1}) for 5 h to remove carbonaceous deposits. This step ensured that an equivalent content of zeolite was measured, enabling direct comparison with the fresh catalyst. Thermogravimetric analysis (TGA) was performed using a Linseis TG-DSC analyser (STA PT 1600). TGA of the catalysts after use in methanol or methyl chloride to hydrocarbon reaction was carried out in diluted oxygen (20 vol.% O_2/Ar, 100 cm^3 min^{-1}), heating the samples (amount fixed to 30 mg) from 298 to 1273 K at 10 K min^{-1} to quantify the amount of coke deposits.

Catalyst evaluation

The reactions of methanol (MTH) and methyl chloride (MCTH) coupling to hydrocarbons were performed at ambient pressure in a continuous-flow fixed-bed reactor setup, depicted in **Supplementary Fig. 32**. The purity of all gases used in this study was graded according to the nines purity scale, wherein 5.0 indicates a purity of 99.999%. CH_3Cl (PanGas; purity, 3.0), and He (carrier gas; PanGas; purity, 5.0), were dosed by a set of digital mass flow controllers (Bronkhorst), while liquid CH_3OH (Sigma-Aldrich, >99.9%) was supplied by a syringe pump (Nexus 6000, Chemyx) equipped with a vaporizer unit operated at 393 K. A quartz reactor (internal diameter, d_i = 8 mm) was loaded with the zeolite (catalyst weight, W_{cat} = 0.135-0.6 g; particle size, d_p = 0.2-0.4 mm). The catalyst bed was pre-treated at 673 K for 1 h under He flow in an electrical oven before switching to the desired reaction temperature (T = 523-723 K). The

reaction mixture was fed at a total volumetric flow (F_T) of 20-400 cm^3 STP min^{-1}, resulting in a contact time (τ) of 0.06-3.75 s. Downstream linings were heated at 473 K to prevent condensation of unconverted reactants and/or products. A liquid trap, using 1-octadecene (Acros, 90%) as solvent and operated at 393 K, was used to separate the C_{5+} hydrocarbon fraction. The reactor-outlet gas stream, containing the reactants and C_1-C_4 products, was quantified online *via* a gas chromatograph equipped with a GS-Carbon PLOT column and a flame ionization detector (GC-FID, Agilent GC 7890A). After GC-FID analysis, the gas stream was passed through an impinging bottle containing an aqueous solution of NaOH (1 M) for neutralization prior to its release in the ventilation system. On the other hand, the liquids collected in the trap were quantified offline *via* a high-performance liquid chromatograph equipped with an Agilent Zorbax Plus C18 column, a diode array detector (235 nm), and a water-acetonitrile gradient (0.5 cm^3 min^{-1}) at 313 K (HPLC, Agilent 1260 Infinity).

The conversion of methanol and methyl chloride in the CH_3X (X = OH, Cl) coupling to hydrocarbons, $X(i)$ (i: CH_3Cl, CH_3OH), was calculated using Eq. 1,

$$X(i) = \frac{n(i)_{\text{in}} - n(i)_{\text{out}}}{n(i)_{\text{in}}} \times 100, \% \qquad \text{Eq. 1}$$

where $n(i)_{\text{in}}$ and $n(i)_{\text{out}}$ are the molar flows of the reactant i at the reactor inlet and outlet, respectively. The selectivity, $S(j)$, to product j (j: CH_4, $(CH_3)_2O$, olefins, paraffins, BTX, other) was calculated according to Eq. 2,

$$S(j) = \frac{n(j)_{\text{out}} \times N_{\text{C}}(j)}{n(i)_{\text{in}} - n(i)_{\text{out}}} \times 100, \% \qquad \text{Eq. 2}$$

where $n(j)_{\text{out}}$ and $N_C(j)$ are the molar flow and the number of carbon atoms of compound j at the reactor outlet. Olefins and paraffins both encompass the C_2-C_4 fraction, BTX represents aromatics such as benzene, toluenes, and xylenes, and other comprises the remaining C_{5+} hydrocarbons.

The error of the carbon balance, ε_C, in all catalytic tests was determined using Eq. 3,

$$\varepsilon_{\text{C}} = \frac{n(i)_{\text{in}} - \left(n(i)_{\text{out}} + \sum n(i)_{\text{out}} \times N_{\text{C}}(j)\right)}{n(i)_{\text{in}}} \times 100, \% \qquad \text{Eq. 3}$$

After the tests, the reactor was quenched to room temperature in He flow, and the catalyst was retrieved for further characterization.

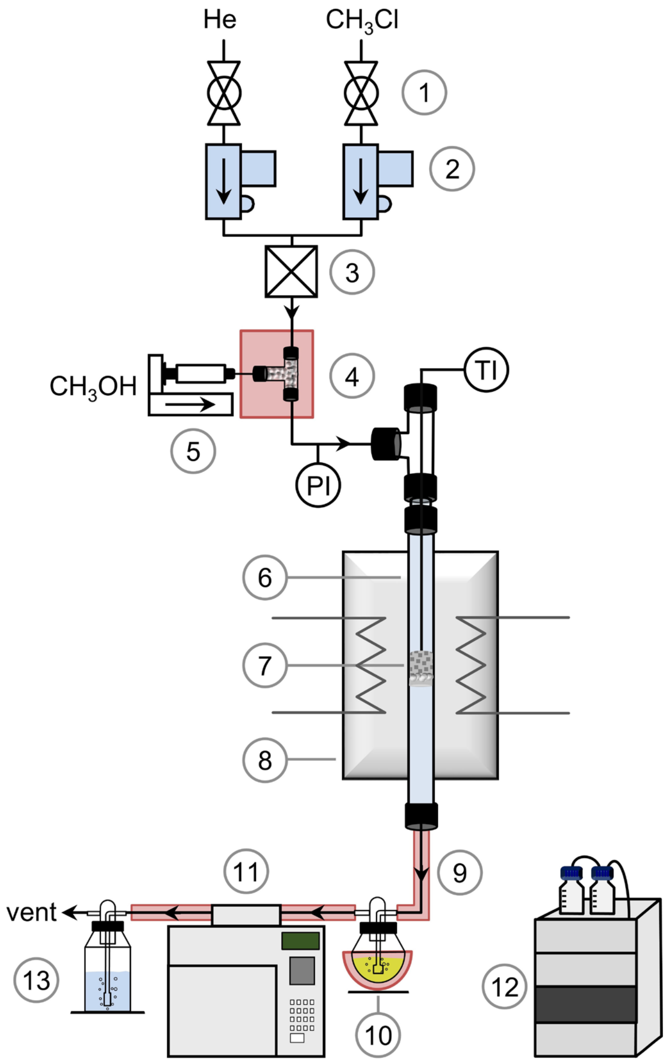

Supplementary Figure 32. Scheme of the customized continuous-flow set-up for methanol (MTH) and methyl chloride (MCTH) to hydrocarbons. 1: two-way on-off valves, 2: mass flow controllers, 3: mixer, 4: vaporizer, 5: syringe pump, 6: quartz reactor, 7: catalyst bed, 8: oven, 9: heat tracing, 10: liquid trap, 11: GC-FID, 12: HPLC, 13: NaOH scrubbers, PI: pressure indicator, and TI: temperature indicator.

Operando photoelectron photoion coincidence spectroscopy

Operando photoelectron photoion coincidence spectroscopy (PEPICO) experiments were performed using the CRF-PEPICO endstation at the vacuum ultraviolet beamline of the Swiss Light Source of the Paul Scherrer Institute, Switzerland. The detection of elusive intermediates during reaction conditions is enabled by coupling a heated microreactor and the gas sampling setup to the detection chamber, as depicted in **Supplementary Fig. 33**. This allows us to minimize the quenching of short-lived species that are desorbed from the catalyst surface or generated in the gas phase above the catalyst by high dilution of the feed and low pressure inside the source chamber (ca. 2×10^{-8} bar) surrounding the reactor outlet. In addition, this configuration enables us to conduct the reaction at near-ambient pressures ($(1\text{-}50)\times10^{-2}$ bar) to extract mechanistic insights at relevant reaction conditions. Reaction products, intermediates, and unconverted reactants form a molecular beam at the reactor outlet. The beam passes through a skimmer, and monochromatic vacuum ultraviolet (VUV) radiation ionizes the constituent molecules. A constant electric field accelerates the resulting photoelectrons and photoions in opposite directions, which are then detected in delayed coincidence. Photoions are analyzed by time-of-flight (TOF) mass spectrometry, which reveals their mass to charge (m/z) ratio, thus indicating the identity of the species (**Supplementary Fig. 33**). In addition, photoion momentum imaging enables us to distinguish background gas contributions from the molecular beam exiting the reactor, as the latter exhibits high speeds along the beam axis and low speeds perpendicular to it. The ionized background gas instead features a room temperature velocity distribution in the x and y directions, resulting in an almost circular spot (**Supplementary Fig. 33**). Photoion momentum imaging enables the discrimination of direct and dissociative photoionization events because the release of kinetic energy in the latter broadens the speed distribution perpendicular to the molecular beam axis. Electrons are also velocity map imaged (**Supplementary Fig. 33**) and act as an ionization time stamp for the ion TOF mass analysis. Accordingly, photoion mass-selected threshold photoelectron (ms-TPE) spectra (**Supplementary Fig. 33**) are derived by integration of electrons with low kinetic energy ($(5\text{-}10)\times10^{-3}$ eV). Vibrational fingerprints in the ms-TPE spectra are characteristic of the molecular structure and enable isomer-selective detection by comparison with reference data or Franck-Condon simulations.

CH_3Cl, Xe (internal standard), and Ar (carrier gas) were dosed by a set of digital mass flow controllers into a heated tubular quartz reactor (2 mm internal diameter) mounted on a molecular beam source in the source chamber. CH_3OH was vaporized at 298 K and 2 bar under Ar atmosphere and dosed by a digital mass flow controller. For each C_1 compound, two main operando PEPICO experiments were conducted at reactor inlet pressures of 5×10^{-2} and 5×10^{-1} bar, respectively. Accordingly, two distinct catalyst loading procedures were used to reach the desired inlet pressures. In the low-pressure experiments, the catalyst (W_{cat} = 0.01 g) was deposited on the inner walls of the reactor by wash-coating, using a catalyst suspension in a mixture of ethanol (Merck, absolute) and deionized water in a volumetric ratio of 50:50. In the near-ambient-pressure experiments, the reactor was packed with the catalyst (W_{cat} = 0.05 g) and a plug of quartz wool was used to maintain the sample in position during testing. Prior to the operando PEPICO experiments, the catalyst was dried in vacuum at 773 K for 1 h. An empty quartz reactor was used for reference experiments.

The quartz reactor was heated by a thermocoax heating wire mounted on a heating sleeve and was connected to a DC power supply (Voltcraft). Its temperature was monitored by a type K thermocouple attached to the outside reactor wall at the midpoint of the heater assembly. The temperature of the flowing gas was calibrated against the measured outside temperature in an independent experiment, which showed that these deviations are in the range of ±30 K. This calibration was used to recalculate the gas temperature during the C_1 coupling experiments. The pressure in the source chamber surrounding the reactor outlet was 2×10^{-8} bar. The central part of the molecular beam leaving the reactor was skimmed (2 mm orifice) upon entering the analysis chamber, operating at a pressure of 2×10^{-9} bar. The synchrotron vacuum ultraviolet (VUV) radiation was collimated and dispersed by a 150 mm^{-1} grating, and focused onto the exit slit (200 μm) located in the gas filter. In the 7-14 eV photon energy range, higher order radiation was suppressed by using an Ar/Ne/Kr mixture (30 vol.% Ar, 10 % Kr, in Ne 5.0) at 1×10^{-2} bar over an optical length of 10 cm. Below 10.6 eV photon energy, a MgF_2 window was used to absorb higher-order light. The photon beam crossed the skimmed molecular beam from the reactor in the ionization region. Photoions and photoelectrons produced upon ionization were accelerated vertically in opposite directions by a constant electrical field of 230 V cm^{-1} and were velocity map imaged onto two delay-line anode detectors (Roentdek, DLD40), and detected in delayed coincidence.[54] Since the electrons have a much shorter flight time (ca. 100 ns) than the ions, they are used as start signal for the ion time-of-flight (TOF) detection in a multi-start multi-stop coincidence scheme. The photon energy was scanned in 0.02 to 0.5 eV steps and averaged for 60-300 s in the photoion mass-selected threshold photoelectron (ms-TPE) spectrum scans. The kinetic energy resolution of the electrons was set to $(5\text{-}10)\times10^{-3}$ eV and faster electrons with a negligible off-axis momentum component were subtracted using the approach by Szátray and Baer.[55] The false coincidence background baseline was also subtracted from the threshold ionisation mass spectra. The species of interest are isomer-selectively assigned by comparison with either reference ms-TPE or PE spectral data,[56-65] or Franck-Condon simulations. Gaussian16 was utilized to calculate ionization energies, equilibrium structures and vibrational modes.[66] Adiabatic ionization energies were computed by optimizing the neutral and ion state of the desired molecule at CBS-QB3 level of theory. ms-TPE spectra were analyzed in the double harmonic approximation using the Franck-Condon approach as implemented in ezSpectrum or Gaussian16.[67] Stick spectra were convoluted by Gaussian function ($fwhm = (2\text{-}5)\times10^{-2}$ eV) to account for the rotational envelope and shifted to the ionisation energy to fit the experimental spectrum. Ions generated from direct ionization of the molecular beam have a characteristic momentum component along the molecular beam axis and are imaged off-centre on the detector. This enables separation from ionization of the background-scattered gas in the ionization chamber as well as helps to distinguish dissociative ionization.[68] The photon energies for the mass spectra were chosen to be higher than the ionization energy of species of interest, but below any dissociative photoionization threshold of larger species that may yield the same mass-to-charge ratio (m/z) fragment ions. Therefore, the ion TOF always corresponds to the mass of the neutral species, while fragmentation is supressed quantitatively. All electrons and ions are correlated to obtain TOF mass spectra used for the kinetic experiments. The signals of neutral and radical species were

recorded at the following *m*/*z* ratios and photon energies ($h\nu$): $CH_3^{\bullet}$ (*m*/*z* 15; $h\nu$ = 10.1 eV), CH_3Cl (*m*/*z* 50/52; $h\nu$ = 11.2 eV), CH_3OH (*m*/*z* 32; $h\nu$ = 11.2 eV), CH_2O (*m*/*z* 30; $h\nu$ = 10.9 eV), $(CH_3)_2O$ (*m*/*z* 46; $h\nu$ = 10.1 eV), C_2H_4 (*m*/*z* 28; $h\nu$ = 10.6 eV), CH_3CHO (*m*/*z* 44; $h\nu$ = 10.5 eV), C_3H_6 (*m*/*z* 42; $h\nu$ = 9.9 eV), C_4H_6 (*m*/*z* 54; $h\nu$ = 9.3 eV), C_4H_8 (*m*/*z* 56; $h\nu$ = 9.3 eV), C_5H_6 (*m*/*z* 66; $h\nu$ = 8.7 eV), C_5H_8 (*m*/*z* 68; $h\nu$ = 8.7 eV), C_5H_{10} (*m*/*z* 70; $h\nu$ = 9.1 eV), C_6H_6 (*m*/*z* 78; $h\nu$ = 9.3 eV), C_6H_8 (*m*/*z* 80; $h\nu$ = 8.5 eV), C_7H_8 (*m*/*z* 92; $h\nu$ = 8.9 eV), C_7H_{10} (*m*/*z* 94; $h\nu$ = 8.3 eV), C_8H_{10} (*m*/*z* 106; $h\nu$ = 8.5 eV), C_9H_{12} (*m*/*z* 120; $h\nu$ = 8.5 eV), $C_{10}H_8$ (*m*/*z* 128; $h\nu$ = 8.3 eV), HCl (*m*/*z* 36/38; $h\nu$ = 12.9 eV), H_2O (*m*/*z* 18, $h\nu$ = 12.9 eV), Xe (*m*/*z* 128/129/130/131/132/134/136; $h\nu$ = 12.9 eV).

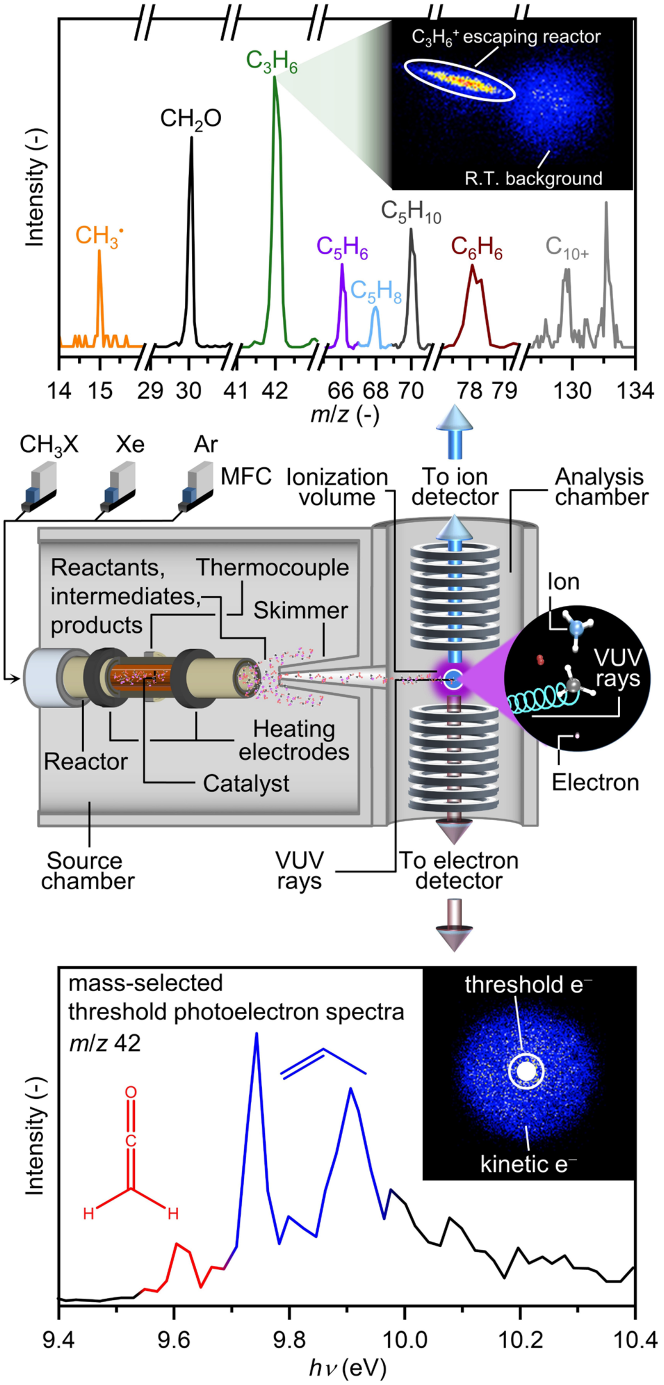

Supplementary Figure 33. Schematic representation of the PEPICO reactor setup for operando isomer-selective detection of short-lived intermediates used in this study. The gases are fed through mass flow controllers (MFC) in a heated quartz reactor coated or packed with H-ZSM-5. The central part of the molecular beam escaping the reactor is photoionized by monochromatic vacuum ultraviolet (VUV) synchrotron radiation, generating ions and electrons. The ions are detected by time-of-flight spectrometry and are velocity imaged to distinguish species formed in the reactor from background or dissociative ionization contributions (shown inset for C_3H_6). Electrons are similarly velocity imaged, allowing us to measure photoion mass-selected threshold photoelectron (ms-TPE) spectra. This analysis provides information on the structure of the ions that generated them, thus enabling isomer-selective detection, as

illustrated for the case of ethenone and propylene. The scope of the depicted mass spectrum is solely to illustrate different intermediates and products of CH_3X (X = OH, Cl) to hydrocarbons that can be detected by PEPICO spectroscopy.

Electron paramagnetic resonance spectroscopy

A comprehensive approach involving electron paramagnetic resonance (EPR) techniques was used in this work to study the density, spatial distribution, and representative molecular structure of the coke deposited in the pores of H-ZSM-5 during conversions of CH_3X to hydrocarbons, as illustrated in **Supplementary Fig. 34**. The paramagnetic nature of these deposits, which allows detection by EPR spectroscopy, originates from electron transfer to the zeolite framework, generating radical species. These are stabilized by electron delocalization within the aromatic rings of the coke species. These electron spins can be probed by varying magnetic and/or microwave fields with distinct EPR techniques, enabling the extraction of different information. First, continuous wave (cw) EPR was conducted, which entails the variation of the magnetic field at constant microwave frequency. The intensity of the corresponding signal is directly proportional to the amount of the contributing paramagnetic species, which can be quantified as detailed below. In addition, by fitting this signal, it is possible to extract the Gaussian and Lorentzian character of its lineshape, which gives information on the packing and local concentration of paramagnetic species. Complementary pulsed EPR techniques, involving short high-power microwave pulses, can provide valuable insights on the spatial distribution and nature of coke. In particular, by analyzing the Hahn echo decay, it is possible to extract the fractal dimension of the carbon deposits. This enables us to elucidate if these grow preferentially in one or two or equally in three dimensions. Finally, 2D hyperfine sublevel correlation (HYSCORE) spectroscopy was conducted to shed light on the chemical nature of coke. This technique entails a series of microwave pulses with different lengths and phases that are separated by fixed or varying delays. This allows to probe the interaction of an unpaired electron with surrounding nuclear spins. The resulting 2D spectra can be modeled to match the detected hyperfine coupling, allowing the assignment of a representative molecular structure.

We performed **cw EPR** spectroscopy experiments on a Bruker EMX spectrometer operating at X-band frequencies (~ 9.2 GHz) using a standard rectangular Bruker EPR cavity (ER4102ST). All cw EPR spectra were acquired with the spectrometer settings given in **Supplementary Table 2**, while all measured *g*-factors were offset-corrected against a known standard, i.e., free radical 1,1-diphenyl-2-picrylhydrazyl (DPPH). These experiments allowed us to quantify the concentration of EPR-active coke spins. This was achieved by following a reported method, which permits accurate estimations by comparing the spin system to an external standard under the same measurement parameters, including *Q*-value, receiver gain, microwave power, sample position, among others as well as appropriate double integral corrections.[69] Solutions of 2,2,6,6-tetramethylpiperidine 1-oxyl (TEMPO) in toluene with known concentrations (10 μM, 100 μM, 1 mM, 10 mM) were used as external spin-counting standards. The broad range of concentrations

was chosen to properly quantify the wide variety of samples. Finally, the standard concentration was further fitted against its corrected double integral to ensure its linear behavior.

Pulsed EPR measurements were performed at room temperature on a Bruker EleXsys E580 spectrometer using a standard dielectric resonator (ER4118X-MD5-W1) and an Oxford helium (CF 935P) cryostat, operating at a frequency of approximately 9.6 GHz. The microwave pulses were amplified using a 1 kW pulsed traveling wave tube (TWT) amplifier. The echo-detected EPR spectra were recorded by applying a $\pi/2$-τ-π echo pulse sequence with $\pi/2$ pulse length of 16 ns and a time delay between the two pulses, τ, of 248 ns. The echo was integrated for a variable static magnetic field, B_0, which was swept in a 10 mT range around the signal maximum. For the transverse relaxation measurements, the amplitude of the Hahn echo as a function of τ was recorded at a magnetic field corresponding to the maximum intensity of the field swept spectrum, where τ was incremented in steps of 4 ns at constant $\pi/2$ pulse length. To gain insights into the spatial distribution of coke radicals, the two-pulse echo-decay measurements were performed at different microwave powers, corresponding to different oscillatory magnetic fields B_1. Then, the decay contribution governed by the instantaneous diffusion, I_{ID}, was extracted by dividing the decay trace with the maximum initial echo intensity, corresponding to a π turning angle of the second pulse by the decay trace at very low microwave field. Since I_{ID} does not noticeably contribute to the echo decay at the latter conditions, the decay rate for the second trace is dominated by all other mechanisms, whose contribution is thus removed by the division. Accordingly, I_{ID} could be expressed by Eq. 4,

$$\ln I_{\mathrm{ID}} = \left\langle \cos\theta - 1 \right\rangle \times k \times \tau^{\frac{N}{3}} \qquad \text{Eq. 4}$$

where θ is the average flip angle of the second microwave pulse, k is a proportionality constant that is proportional to the fractal local concentration of spins coupled by dipolar interactions that are affected by the microwave pulses, and N is a fractional dimension that varies between 1 and 3. This fractional dimension indicates the topology of the coke spins distribution, with $N = 1$ corresponding to a one-dimensional (linear chain) and $N = 3$ to a homogeneous three-dimensional distribution.

2D HYSCORE spectroscopy was performed at 50 K on a Bruker EleXsys E580 spectrometer using a standard dielectric resonator (ER4118X-MD5-W1) and an Oxford helium (CF 935P) cryostat and operating at a frequency of approximately 9.6 GHz. The measurements were conducted by applying a $\pi/2$-τ-$\pi/2$-t_1-π-t_2-$\pi/2$-τ echo pulse sequence with $\pi/2$ pulse length of 24 ns and an inversion π pulse length of 16 ns with time delays, τ, of 96, 128, and 224 ns to avoid blind-spot artifacts. The 2D HYSCORE time-domain data were recorded by measuring the echo amplitude as a function of dimensions t_1 and t_2, incremented in steps of 8 ns. The pulse sequence was repeated in an 8-step phase cycling procedure to avoid undesired echoes. The obtained spectra were then processed by (*i*) subtracting the background decay using a polynomial function, (*ii*) zero filling to 1024 points, (*iii*) tapering using a Hamming window, and by (*iv*) Fourier transforming the data in both t_1 and t_2 dimensions. The simulations of the hyperfine interactions of different molecular structures were performed applying Kohn-Sham density functional theory and using a B3LYP

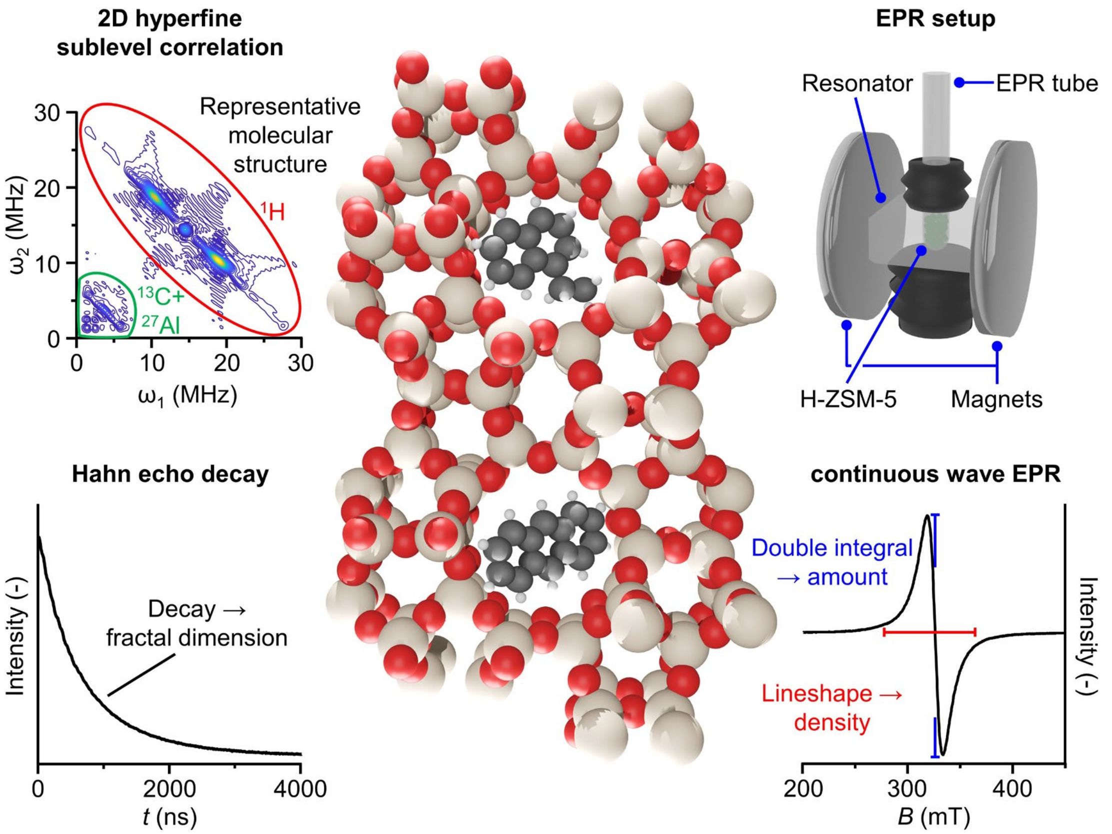

Supplementary Fig. 34. Multi-technique EPR approach used to study the density, spatial distribution, and nature of the coke deposited in the pores of H-ZSM-5 during the conversion of CH_3X to hydrocarbons (shown schematically). Electron delocalization is favored over aromatic carbonaceous species, which are paramagnetic and thus susceptible to microwave and static magnetic fields that can be varied in a typical EPR setup. Accordingly, these fields can be varied to probe distinct features of the paramagnetic species within coke. By conducting continuous wave EPR, which varies the magnetic field at constant microwave frequency, a signal can be obtained, whose double integral and lineshape gives quantitative information on the amount and density of paramagnetic coke. Complementary analysis of the Hahn echo decay obtained by microwave pulsing permits estimation of the fractal dimension of the carbon deposits, indicating if these grow with a preferentially one-, two-, or three-dimensional spatial distribution. Finally, by conducting 2D hyperfine sublevel correlation spectroscopy, entailing a sequence of microwave pulses with different length and phases, the interaction of an unpaired electron with its surrounding nuclear spins can be probed. The resulting 2D spectra can be modeled to match the detected hyperfine coupling, allowing the assignment of a representative molecular structure.

functional with a spin-unrestricted shell and a 6-31G+(d,p) basis set in the Gaussian software. All calculated hyperfine interactions are shown in **Supplementary Table 3**. In addition, the 2D HYSCORE spectra were compared with literature data.[38,46-48,51,52,70] All the cw and 2D HYSCORE spectra were simulated using EasySpin routines in the MATLAB software.

Supplementary references

1. Haw, J. F., Song, W., Marcus, D. M. & Nicholas, J. B. The mechanism of methanol to hydrocarbon catalysis. *Acc. Chem. Res.* **36**, 317-326 (2003).
2. Stöcker, M. Methanol-to-hydrocarbons: catalytic materials and their behaviour. *Micropor. Mespor. Mater.* **29**, 3-48 (1999).
3. Sun, X., Mueller, S., Liu, Y., Shi, H., Haller, G. L., Sanchez-Sanchez, M., van Veen, A. C. & Lercher, J. A. On reaction pathways in the conversion of methanol to hydrocarbons on HZSM-5. *J. Catal.* **317**, 185-197 (2014).
4. Olsbye, U., Svelle, S., Bjørgen, M., Beato, P., Janssens, T. V., Joensen, F., Bordiga, S. & Lillerud, K. P. Conversion of methanol to hydrocarbons: how zeolite cavity and pore size controls product selectivity. *Angew. Chem. Int. Ed.* **51**, 5810-5831 (2012).
5. Chang, C. D. & Silvestri, A. J. The conversion of methanol and other O-compounds to hydrocarbons over zeolite catalysts. *J. Catal.* **47**, 249-259 (1977).
6. Chen, N. Y. & Reagan, W. J. Evidence of autocatalysis in methanol to hydrocarbon reactions over zeolite catalysts. *J. Catal.* **59**, 123-129 (1979).
7. Song, W., Marcus, D. M., Fu, H., Ehresmann, J. O. & Haw, J. F. An oft-studied reaction that may never have been: Direct catalytic conversion of methanol or dimethyl ether to hydrocarbons on the solid acids HZSM-5 or HSAPO-34. *J. Am. Chem. Soc.* **124**, 3844-3845 (2002).
8. Lesthaeghe, D., Van Speybroeck, V., Marin, G. B. & Waroquier, M. Understanding the failure of direct C-C coupling in the zeolite-catalyzed methanol-to-olefin process. *Angew. Chem.* **118**, 1746-1751 (2006)
9. Svelle, S., Joensen, F., Nerlov, J., Olsbye, U., Lillerud, K. P., Kolboe, S. & Bjørgen, M. Conversion of methanol into hydrocarbons over zeolite H-ZSM-5: Ethene formation is mechanistically separated from the formation of higher alkenes. *J. Am. Chem. Soc.* **128**, 14770-14771 (2006).
10. Marcus, D. M., McLachlan, K. A., Wildman, M. A., Ehresmann, J. O., Kletnieks, P. W. & Haw, J. F. Experimental evidence from H/D exchange studies for the failure of direct C-C coupling mechanisms in the methanol-to-olefin process catalyzed by HSAPO-34. *Angew. Chem. Int. Ed.* **45**, 3133-3136 (2006).
11. Olsbye, U., Svelle, S., Bjørgen, M., Beato, P., Janssens, T. V., Joensen, F., Bordiga, S. & Lillerud, K. P. Conversion of methanol to hydrocarbons: how zeolite cavity and pore size controls product selectivity. *Angew. Chem. Int. Ed.* **51**, 5810-5831 (2012).
12. Sun, X., Mueller, S., Shi, H., Haller, G. L., Sanchez-Sanchez, M., van Veen, A. C. & Lercher, J. A. On the impact of co-feeding aromatics and olefins for the methanol-to-olefins reaction on HZSM-5. *J. Catal.* **314**, 21-31 (2014).
13. Mole, T., Whiteside, J. A. & Seddon, D. Aromatic co-catalysis of methanol conversion over zeolite catalysts. *J. Catal.* **82**, 261-266 (1983).

14. Mole, T., Bett, G. & Seddon, D. Conversion of methanol to hydrocarbons over ZSM-5 zeolite: An examination of the role of aromatic hydrocarbons using 13carbon-and deuterium-labeled feeds. *J. Catal.* **84**, 435-445 (1983).
15. Wang, C., Xu, J., Qi, G., Gong, Y., Wang, W., Gao, P., Wang, Q., Feng, N., Liu, X. & Deng, F. Methylbenzene hydrocarbon pool in methanol-to-olefins conversion over zeolite H-ZSM-5. *J. Catal.* **332**, 127-137 (2015).
16. Bjørgen, M., Joensen, F., Lillerud, K. P., Olsbye, U. & Svelle, S. The mechanisms of ethene and propene formation from methanol over high silica H-ZSM-5 and H-beta. *Catal. Today* **142**, 90-97 (2009).
17. Ilias, S. & Bhan, A. Mechanism of the catalytic conversion of methanol to hydrocarbons. *ACS Catal.* **3**, 18-31 (2013).
18. Marcus, D. M., Hayman, M. J., Blau, Y. M., Guenther, D. R., Ehresmann, J. O., Kletnieks, P. W. & Haw, J. F. Mechanistically significant details of the H/D exchange reactions of propene over acidic zeolite catalysts. *Angew. Chem. Int. Ed.* **45**, 1933-1935 (2006).
19. Bjørgen, M., Bonino, F., Kolboe, S., Lillerud, K. P., Zecchina, A. & Bordiga, S. Spectroscopic evidence for a persistent benzenium cation in zeolite H-beta. *J. Am. Chem. Soc.* **125**, 15863-15868 (2003).
20. Chua, Y. T., Stair, P. C., Nicholas, J. B., Song, W. & Haw, J. F. UV Raman spectrum of 1,3-dimethylcyclopentenyl cation adsorbed in zeolite H-MFI. *J. Am. Chem. Soc.* **125**, 866-867 (2003).
21. Yarulina, I., Chowdhury, A. D., Meirer, F., Weckhuysen, B. M. & Gascon, J. Recent trends and fundamental insights in the methanol-to-hydrocarbons process. *Nat. Catal.* **1**, 398-411 (2018).
22. Chowdhury, A. D. & Gascon, J. The curious case of ketene in zeolite chemistry and catalysis. *Angew. Chem. Int. Ed.* **57**, 14982-14985 (2018).
23. Chang, C. D., Hellring, S. D. & Pearson, J. A. On the existence and role of free radicals in methanol conversion to hydrocarbons over HZSM-5: I. Inhibition by NO. *J. Catal.* **115**, 282-285 (1989).
24. Clarke, J. K., Darcy, R., Hegarty, B. F., O'Donoghue, E., Amir-Ebrahimi, V. & Rooney, J. J. Free radicals in dimethyl ether on H-ZSM-5 zeolite. A novel dimension of heterogeneous catalysis. *Chem. Commun.* **5**, 425-426 (1986).
25. Howe, R. F., Gibson, E. K., Catlow, C. R. A., Hameed, A., McGregor, J., Collier, P., Parker, S. F. & Lennon, D. An assessment of hydrocarbon species in the methanol-to-hydrocarbon reaction over a ZSM-5 catalyst. *Faraday Discuss.* **197**, 447-471 (2017).
26. Zhang, W., Zhang, M., Xu, S., Gao, S., Wei, Y. & Liu, Z. Methylcyclopentenyl cations linking initial stage and highly efficient stage in methanol-to-hydrocarbon process. *ACS Catal.* **10**, 4510-4516 (2020).
27. Minova, I. B., Matam, S. K., Greenaway, A., Catlow, C. R. A., Frogley, M. D., Cinque, G., Wright, P. A. & Howe, R. F. Elementary steps in the formation of hydrocarbons from surface methoxy groups in HZSM-5 seen by synchrotron infrared microspectroscopy. *ACS Catal.* **9**, 6564-6570 (2019).

28. Chowdhury, A. D., Paioni, A. L., Houben, K., Whiting, G. T., Baldus, M. & Weckhuysen, B. M. Bridging the gap between the direct and hydrocarbon pool mechanisms of the methanol-to-hydrocarbons process. *Angew. Chem. Int. Ed.* **57**, 8095-8099 (2018).
29. Liu, Y., Kirchberger, F. M., Müller, S., Eder, M., Tonigold, M., Sanchez-Sanchez, M. & Lercher, J. A. Critical role of formaldehyde during methanol conversion to hydrocarbons. *Nat. Commun.* **10**, 1462 (2019).
30. Magnoux, P., Roger, P., Canaff, C., Fouche, V., Gnep, N. S. & Guisnet, M. New technique for the characterization of carbonaceous compounds responsible for zeolite deactivation. *Stud. Surf. Sci. Catal.* **34**, 317-330 (1987).
31. Lee, S. & Choi, M. Unveiling coke formation mechanism in MFI zeolites during methanol-to-hydrocarbons conversion. *J. Catal.* **375**, 183-192 (2019).
32. Mores, D., Kornatowski, J., Olsbye, U. & Wekhuysen, B. M. Coke formation during the methanol-to-olefin conversion: in situ microspectroscopy on individual H-ZSM-5 crystals with different Brønsted acidity. *Chem. Eur. J.* **17**, 2874-2884 (2011).
33. An, H., Zhang, F., Guan, Z., Liu, X., Fan, F. & Li, C. Investigating the coke formation mechanism of H-ZSM-5 during methanol dehydration using operando UV–Raman spectroscopy. *ACS Catal.* **8**, 9207-9215 (2018).
34. Chua, Y. T. & Stair, P. C. An ultraviolet Raman spectroscopic study of coke formation in methanol to hydrocarbons conversion over zeolite H-MFI. *J. Catal.* **213**, 39-46 (2003).
35. Meinhold, R. H. & Bibby, D. M. ^{13}C CPMAS NMR study of coke formation on HZSM-5. *Zeolites* **10**, 121-130 (1990).
36. Echevskii, G. V., Kalinina, N. G., Anufrienko, V. F. & Poluboyarov V. A. ESR studies of coke formation on zeolite catalysts for methanol conversion. *React. Kinet. Catal, Lett.* **33**, 305-310 (1987).
37. Bauer, F. & Karge, H. G. Characterization of coke on zeolites. In: Karge, H. G., Weitkamp, J. (Eds.) Characterization II. Molecular Sieves, Vol. 5. Springer, Berlin, Heidelberg (2006).
38. Suwardiyanto, Howe, R. F., Gibson, E. K., Catlow, C. R. A., Hameed, A., McGregor, J., Collier, P., Parkerbg, S. F. & Lennon, D. An assessment of hydrocarbon species in the methanol-to-hydrocarbon reaction over a ZSM-5 catalyst. *Faraday Discuss.* **197**, 447 (2017).
39. Tayeb, K. B. et al. Ethanol transformation into higher hydrocarbons over HZSM-5 zeolite: Direct detection of radical species by in situ EPR spectroscopy. *Catal. Commun.* **27**, 119-123 (2012).
40. Mores, D., Kornatowski, J., Olsbye, U. & Weckhuysen, B. M. Coke formation during the methanol-to-olefin conversion: in situ microspectroscopy on individual H-ZSM-5 crystals with different Brønsted acidity. *Chem. Eur. J.* **17**, 2874-2884 (2011).
41. Chua, Y. T. & Stair, P. C. An ultraviolet Raman spectroscopic study of coke formation in methanol to hydrocarbons conversion over zeolite H-MFI. *J. Catal.* **213**, 39-46 (2003).
42. Lin, R., Amrute, A. P. & Perez-Ramirez, J. Halogen-mediated conversion of hydrocarbons to commodities. *Chem. Rev.* **117**, 4182-4247 (2017).

43. Sun, Y., Campbell, S. M., Lunsford, J. H., Lewis, G. E., Palke, D. A. L. E. & Tau, L. M. The catalytic conversion of methyl chloride to ethylene and propylene over phosphorus-modified Mg-ZSM-5 zeolites. *J. Catal.* **143**, 32-44 (1993).
44. Murray, D. K., Chang, J. W. & Haw, J. F. Conversion of methyl halides to hydrocarbons on basic zeolites: A discovery by in situ NMR. *J. Am. Chem. Soc.* **115**, 4732-4741 (1993).
45. Bukhtiyarova, M. V. & Echevskii, G. V. Coke formation on zeolites Y and their deactivation model. *Pet. Chem.* **60**, 532-539 (2020).
46. Pradhan, A. R. et al. EPR and NMR studies of coke induced selectivation over H–ZSM-5 zeolite during ethylbenzene disproportionation reaction. *J. Catal.* **184**, 29-38 (1999).
47. Pinard, L. et al. Growth mechanism of coke on HBEA zeolite during ethanol transformation. *J. Catal.* **299**, 284-297 (2013).
48. Tayeba, K. B. et al. The radical internal coke structure as a fingerprint of the zeolite framework, *Microporous Mesoporous Mater.* **289**, 109617 (2019).
49. Kittel, C. & Abrahams, E. Dipolar broadening of magnetic resonance lines in magnetically diluted crystals. *Phys. Rev.* **90**, 238-239 (1953).
50. Binet, L., Gourier, D., Derenne, S., Robert, F. & Ciofini, I. Occurence of abundant diradicaloid moieties in the insoluble organic matter from the Orgueil and Murchison meteorites: a fingerprint of its extraterrestrial origin? *Geochim. Cosmochim. Acta* **68**, 881-891 (2004).
51. Spencer, N. A., Ditzel, E. J., Hargreaves, J. S. J. & Sproules, S. Radicals in carbonaceous residue deposited on mordenite from methanol. *J. Mater. Chem. A* **4**, 7036-7044 (2016).
52. Ikoma, T., Ito, O., Tero-Kubota, S. & Akiyama, K. HYSCORE study on coal radicals. *Energ. Fuels* **12**, 1363-1368 (1998).
53. Emeis, C. A. Determination of integrated molar extinction coefficients for infrared absorption bands of pyridine adsorbed on solid acid catalysts. *J. Catal.* **141**, 347-354 (1993).
54. Bodi, A., Sztáray, B., Baer, T., Johnson, M. & Gerber, T. Data acquisition schemes for continuous two-particle time-of-flight coincidence experiments. *Rev. Sci. Instrum.* **78**, 84102 (2007).
55. Sztáray, B. & Baer, T. Suppression of hot electrons in threshold photoelectron photoion coincidence spectroscopy using velocity focusing optics. *Rev. Sci. Instrum.* **74**, 3763-3768 (2003).
56. Paunović, V., Hemberger, P., Bodi, A., López, N. & Pérez-Ramírez, J. Evidence of radical chemistry in catalytic methane oxybromination. *Nat. Catal.* **1**, 363-370 (2018).
57. Carlier, J. & Botter, R. Photoelectron spectra of ethylene and of the six deuterated derivatives. *J. Electron Spectrosc. Relat. Phenomena* **17**, 91-99 (1979).
58. Yencha, A. J., Siggel-King, M. R. F., King, G. C., Malins, A. E. R. & Eypper, M. Threshold photoelectron spectroscopy of acetaldehyde and acrolein. *J. Electron Spectrosc.* **187**, 65-71 (2013).
59. Butler, J. J., Holland, D. M. P., Parr, A. C. & Stockbauer, R. A threshold photoelectron-photoion coincidence spectrometric study of dimethyl ether (CH_3OCH_3). *Int. J. Mass Spectrom.* **58**, 1-14 (1984).

60. Felsmann, D. *et al.* Progress in fixed-photon-energy time-efficient double imaging photoelectron/photoion coincidence measurements in quantitative flame analysis. *Z. Phys. Chem.* **230** (2016).
61. Pieper, J. *et al.* Isomer identification in flames with double-imaging photoelectron/photoion coincidence spectroscopy (i^2PEPICO) using measured and calculated reference photoelectron spectra. *Z. Phys. Chem.* **232**, 153-187 (2018).
62. McCabe, M. N., Hemberger, P., Reusch, E., Bodi, A. & Bouwman, J. Off the beaten path: almost clean formation of indene from the ortho-benzyne + allyl reaction. *J. Phys. Chem. Lett.* **11**, 2859-2863 (2020).
63. Mayer, P. M., Blanchet, V. & Joblin, C. Threshold photoelectron study of naphthalene, anthracene, pyrene, 1,2-dihydronaphthalene, and 9,10-dihydroanthracene. *J. Chem. Phys.* **134**, 244312 (2011).
64. Worley, S. D. & Webb, T. R. The electronic structure of transition-metal carbonyl complexes of norbornadiene and mesitylene. *J. Organomet. Chem.* **192**, 139-145 (1980).
65. Shudo, K., Kobayashi, T. & Utsunomiya, C. Photoelectron spectral studies on the interaction of three-membered rings with aryl groups. *Tetrahedron* **33**, 1721-1724 (1977).
66. Frisch, M. J. *et al.* Gaussian 16 v. revision C. 01 (Gaussian, Inc., Wallingford CT, 2016).
67. Gozem, S. & Krylov, A. I. The ezSpectra suite: An easy-to-use toolkit for spectroscopy modeling v. WIRES CMS, e1546 (Los Angeles, USA, 2021).
68. Hemberger, P., van Bokhoven, J. A., Pérez-Ramírez, J. & Bodi, A. New analytical tools for advanced mechanistic studies in catalysis: photoionization and photoelectron photoion coincidence spectroscopy. *Catal. Sci. Technol.* **10**, 1975-1990 (2020).
69. Zichittella, G., Polyhach, Y., Tschaggelar, R., Jeschke, G. & Pérez-Ramírez, J. Quantification of redox sites during catalytic propane oxychlorination by operando EPR spectroscopy. *Angew. Chem. Int. Ed.* **60**, 3596-3602 (2021).
70. Madeira, F. F. *et al.* Radical species detection and their nature evolution with catalyst deactivation in the ethanol-to-hydrocarbon reaction over HZSM-5 zeolite. *ACS Catal.* **1**, 417-424 (2011).
